# Proteomic profiling of proteins associated with the rejuvenation of *Sequoia sempervirens *(D. Don) Endl

**DOI:** 10.1186/1477-5956-8-64

**Published:** 2010-12-10

**Authors:** Ing-Feng Chang, Peng-Jen Chen, Chin-Hui Shen, Tsung-Ju Hsieh, Ya-Wen Hsu, Bau-Lian Huang, Ching-I Kuo, Yu-Ting Chen, Hsiu-An Chu, Kai-Wun Yeh, Li-Chun Huang

**Affiliations:** 1 Institute of Plant Biology, National Taiwan University, Taipei 106, Taiwan; 2 Institute of Plant and Microbial Biology, Academia Sinica, Taipei, Taiwan; 3 Institute of Genomics and Bioinformatics, National Chung Hsing University, Taichung, Taiwan

## Abstract

**Background:**

Restoration of rooting competence is important for rejuvenation in *Sequoia sempervirens *(D. Don) Endl and is achieved by repeatedly grafting *Sequoia *shoots after 16 and 30 years of cultivation *in vitro*.

**Results:**

Mass spectrometry-based proteomic analysis revealed three proteins that differentially accumulated in different rejuvenation stages, including oxygen-evolving enhancer protein 2 (OEE2), glycine-rich RNA-binding protein (RNP), and a thaumatin-like protein. OEE2 was found to be phosphorylated and a phosphopeptide (YEDNFDGNSNVSVMVpTPpTDK) was identified. Specifically, the protein levels of OEE2 increased as a result of grafting and displayed a higher abundance in plants during the juvenile and rejuvenated stages. Additionally, *SsOEE2 *displayed the highest expression levels in *Sequoia *shoots during the juvenile stage and less expression during the adult stage. The expression levels also steadily increased during grafting.

**Conclusion:**

Our results indicate a positive correlation between the gene and protein expression patterns of *SsOEE2 *and the rejuvenation process, suggesting that this gene is involved in the rejuvenation of *Sequoia sempervirens*.

## Background

Plant maturation involves sequential developmental stages or phases that can be categorized as embryo, juvenile, transitional, and adult. The development of reproductively mature adult plants usually begins with a strictly vegetative juvenile phase. Maturation or a phase change is completed within weeks among annuals but can proceed for several years among perennials. In trees, the process is frequently accompanied by ancillary morphological and physiological traits, most commonly a loss of competence for adventitious rooting and a loss of overall vigor. Because a plant's developmental phase is determined in its shoot apical meristems, reversing the phase of the meristems should result in the emergence of rejuvenated shoots [1]. Indeed, new growths with reversed phases have been obtained by applying gibberellin [2] and cytokinin [3,4], continuously subculturing shoots, especially in cytokinin-containing media [5-7], and repeatedly grafting shoot apices from mature trees onto juvenile rootstocks *in vivo *[8,9] and *in vitro *[10-15].

*Sequoia sempervirens *(D. Don) Endl, a coastal redwood, can be rejuvenated through repeatedly grafting its adult-phase shoot tips onto juvenile rootstocks *in vitro *[16], which can result without phytohormonal supplements. Adventitious rooting is notably depressed and essentially absent in tissues of adult trees. The stability of the rejuvenated state by restoring rooting competence and other juvenile characteristics can be retained *in vitro *even after 30 years. The reversion has been associated with distinctive leaf proteins, including clearly apparent changes in iso-esterase and iso-peroxidase [17], different tyrosine phosphorylation patterns, and higher total protein phosphorylation in juvenile shoots [18]. Juvenile and rejuvenated shoots also released more ethylene [19], had higher total nitrogen content, and were more active in photosynthesis and respiration [20]. This diversity of differences in physiology between juvenile and adult *Sequoia *shoots reflect the complexity of the developmental phase change.

In 1991, a 16 kDa protein was found to be only produced in juvenile or rejuvenated meristems [21]. In 1996, a 28 kDa protein was found to be expressed in greater amounts in juvenile shoot tips than mature shoot tips [22]. Moreover, Gil et al. (2003) identified a cDNA clone termed *Quercus robur *crown preferentially expressed (*QRCPE*) that was differentially expressed in juvenile-like and mature shoots in *Quercus robur*. *QRCPE *appeared to be a cell-wall protein [23]. However, the proteomic differences in adult, juvenile, and rejuvenated tree species are not well known. Using a proteomics approach, candidate regulatory components of the rejuvenation of tree species may be identified. In the present study, *Sequoia *was utilized as our rejuvenation system, and gel-based proteomic analysis was performed. Three proteins with differential abundance in adult, juvenile, and rejuvenated *Sequoia *were identified by mass spectrometry (MS), including oxygen-evolving enhancer protein 2 (OEE2), glycine-rich RNA-binding protein (RNP) and a thaumatin-like protein (TLP). Results from the gene expression and protein accumulation pattern indicated a positive correlation between OEE2 and the rejuvenation stages, suggesting that OEE2 may be involved in the rejuvenation of *Sequoia*.

## Results

### The restored rooting competence of repeatedly grafted Sequoia shoots is retained after 30 years of culture in vitro

**Figure 1:**
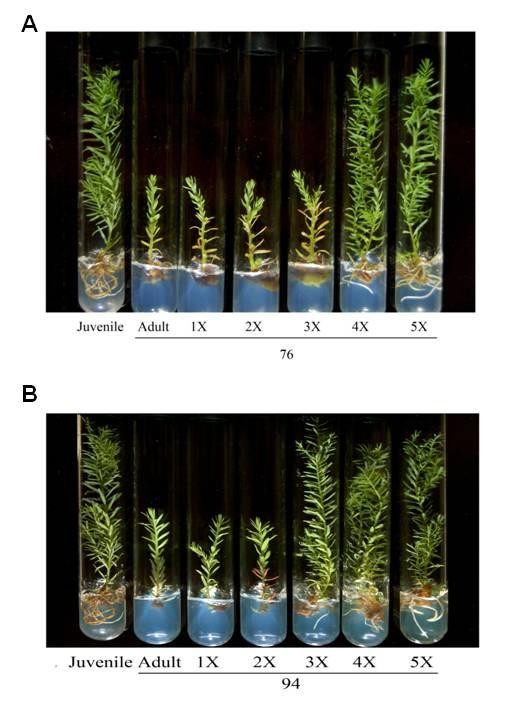
**The restoration of rooting competence in *Sequoia *shoots established in (A) 1976 and (B) 1994**. Left to right: juvenile, adult, and adults with one, two, three, four, or five grafts.

*Sequoia *shoot cultures initiated in 1976 and 1994 had retained their hallmark characteristics. Those in the adult phase were rootless, had enervated shoots, and grew very slowly compared with juvenile phase cultures (Figure 1). These developmental stage-specific samples included adult cultured shoots established in 1976 (Adult76) and 1994 (Adult94) and juvenile and rejuvenated (Rejuvenated) shoots. However, rooting was substantially restored among adult shoots that had been grafted four times onto juvenile rootstocks in 1976 and after three times in 1994.

### Two-dimensional gel electrophoresis of proteins

Mass spectrometry (MS)-based proteomic analysis has been widely used to identify differentially expressed proteins at different stages of plant development [24]. Huang et al. (1992) used two-dimensional gel electrophoresis and discovered pattern differences among proteins extracted from adult, juvenile, and rejuvenated shoot cultures, in which the rejuvenated cultures were obtained by repeatedly grafting adult onto juvenile stock [16]. However, the proteins were not identified. Thus, the present study identified some of the differentially expressed proteins.

**Figure 2:**
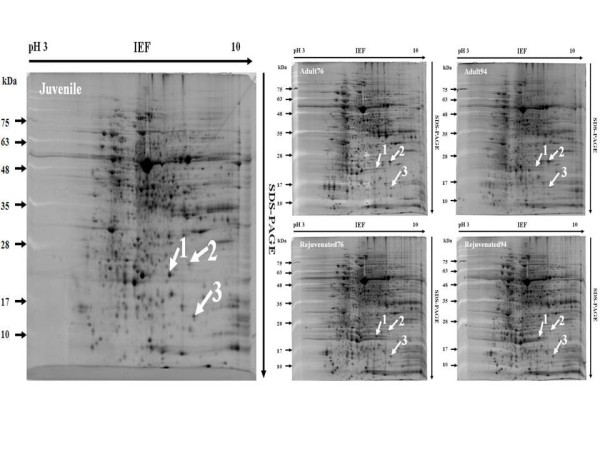
**Two-dimensional gel electrophoresis of total proteins of *Sequoia *at different stages**. Juvenile, juvenile shoots; Adult76 and Adult94, adult shoots established in 1976 and 1994, respectively; Rejuvented76 and Rejuvented94, rejuvenated plants from adult shoots established in 1976 and 1994, respectively. Spot numbers are labeled with white arrows pointing to each protein spot.

Total proteins were isolated from A cultured shoots established in 1976 (Adult76) and 1994 (Adult94) and juvenile and rejuvenated cultured shoots (Rejuvenated76 and Rejuvenated94). The protein extracts were subjected to two-dimensional gel electrophoresis with at least three biological replicates analyzed for each type of tissue. The protein gel images are shown in Figure 2. At least three distinctive protein spots with differential intensity were detected. The relative intensity of each protein spot was quantified using SameSpots software (Progenesis, USA).

### Identification of Sequoia proteins exhibiting up- or down-regulated protein abundance during rejuvenation

**Figure 3:**
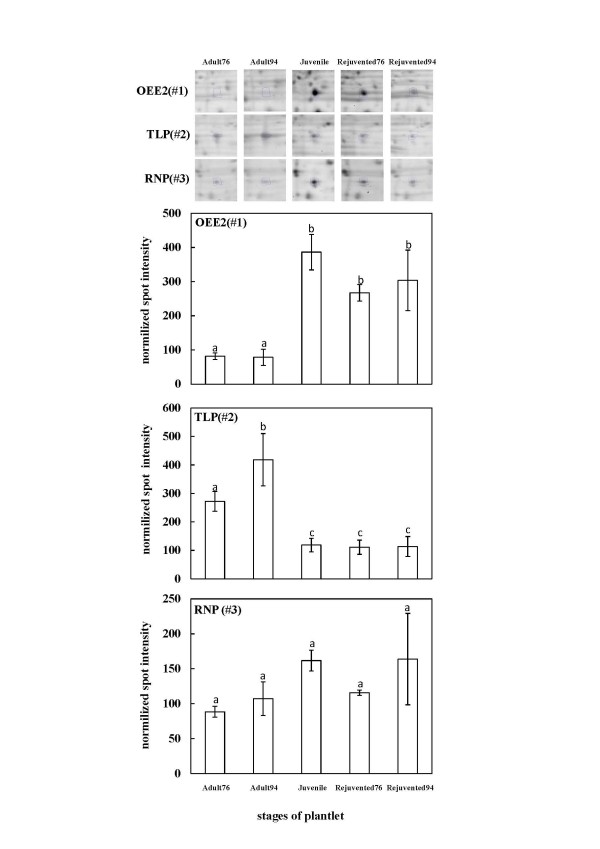
**Diagram and quantification of OEE2, TLP, and RNP in different stages**. (A) Juvenile, juvenile shoots; Aduult76 and Adult94, adult shoots established in 1976 and 1994, respectively; Rejuvenated76 and Rejuvenated94, rejuvenated plants from adult shoots established in 1976 and 1994, respectively. Identified spots are demarcated by the blue line in each gel. (B) Means of the samples were analyzed by ANOVA. Bars having different letters are significantly different (*p *< 0.05, ANOVA followed by Duncan's multiple range test).

Three protein spots that showed differential accumulation (labeled 1, 2, and 3) in each gel were excised and subjected to in-gel digestion followed by MS (Figure 2). Proteins were identified by a database search using in-house Mascot software (Table 1). Protein identification was validated using Scaffold software to provide confidence interval percentages (Additional file 1). Oxygen-evolving enhancer protein2 (OEE2), glycine-rich RNA binding protein (RNP) and thaumatin-like protein (TLP) were consistently identified in the three biological replicates. Specifically, two identified proteins, RNP and OEE2, showed increased protein abundance during the rejuvenation stage (Figure 3), whereas TLP showed decreased protein abundance during the rejuvenation stage (Figure 3). In addition to spots 1, 2, and 3, three other proteins from other spots were also identified. The proteins included glyoxysomal malate dehydrogenase, class I chitinase and a LRR-repeat protein (Additional file 2). However, these proteins were not consistently identified in all five protein gel samples.

**Table 1:** Identification of proteins differentially accumulated in different rejuvenation cultures of Sequoia sempervirens.

Spot Number	Protein Name	PS^a^	pI	Mass	Entry mass	Theoretical mass	Peptide Score	Peptide Sequence
1	Oxygen-evolving enhancer protein 2	2017	6.02	27705	848.1996	848.3512	16	TADGDEGGK
	(N = 3)				1188.3712	1188.6139	49	EVEYPGQVLR
					1227.3166	1227.5772	54	FVESAASSFNVA
					1243.3977	1242.6608	32	QYYTLSVLTR
					1254.4093	1254.6568	71	HQLISATVSDGK
					1294.3918	1294.6193	89	AYGEAANVFGAPK
					1356.4217	1355.6721	27	KFVESAASSFNVA
					1477.4212	1477.6725	84	NTDFITYSGEGFK
					1605.4551	1605.7675	56	KNTDFITYSGEGFK
					2084.6029	2084.9975	90	TADGDEGGKHQLISATVSDGK
					2246.5382	2246.9638	75	YEDNFDGNSNVSV**M**VTPTDK^b^
					2390.6106	2390.9015	56	YEDNFDGNSNVSVMVpTPpTDK^c^
					2374.6248	2375.0587	77	YEDNFDGNSNVSV**M**VTPTDKK^b^
					2688.8683	2689.3195	110	TASEGGFDTNAVATAALLESGNPVVNGK

2	Thaumatin-like protein	833	8.97	24687	1056.2335	1056.4295	83	TG**C**SFDASGR^d^
	(N = 3)				1271.4018	1271.5605	62	GQ**C**PQAYSYAK^d^
					1764.3805	1763.7309	31	DDATSTFT**C**PSGTNYK^d^
					1863.5394	1863.9070	89	IT**C**LSDINSK**C**PSELK^d^

3	Glycine-rich RNA-binding protein 2	1155	5.18	18530	963.2489	963.4661	76	ASAEIEFR + N-acetyl
	(N = 3)				1031.3243	1031.5360	37	IVSDRETGR
					1129.4603	1129.5840	54	NITVNQAQSR
					1165.3100	1165.5225	43	SGGGGYGGGGRER
					1486.3225	1486.6186	114	YGGGSGGYGGGAGGGGGSR
					1699.6421	1699.8601	46	ELDGRNITVNQAQSR
					1761.5227	1761.8574	72	SLHDAFSPFGEVLESK
					2542.5518	2543.0655	18	GFGFVTFSDEQA**MM**DAIEA**M**NGK^b^
					3112.7218	3113.3416	59	GFGFVTFSDEQA**MM**DAIEA**M**NGKELDGR^b^

a: Mascot protein score; b: **M**: oxidized methionine; c: p: Threonine phosphorylation; d: **C**: carbamidomethyl modified cysteine.

**Figure 4:**
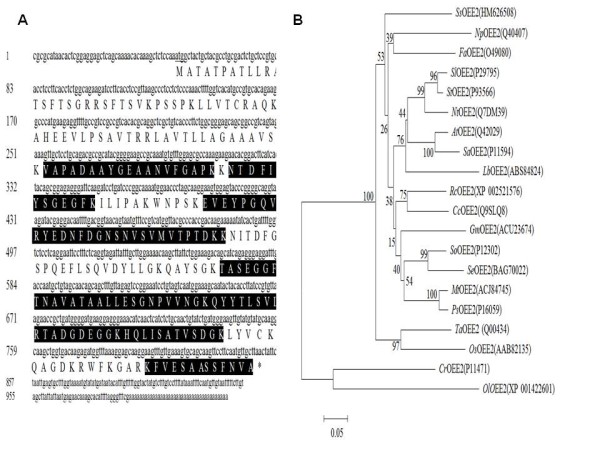
**Sequence coverage and phylogenetic tree of oxygen-evolving enhancer protein 2**. (A) Lowercase and capital letters represent the cDNA and amino acid sequence, respectively. Highlighted amino acids indicate the coverage fragments with LC-MS/MS. (B) At, *Arabidopsis thaliana*; Cc, *Cucumis sativus*; Cr, *Chlamydomonas reinhardtii*; Fa, *Fritillaria agrestis*; Gm, *Glycine max*; Lb, *Limonium bicolor*; Mt, *Medicago truncatula*; Nt, *Nicotiana tabacum*; Np, *Narcissus pseudonarcissus*; Ol, *Ostreococcus lucimarinus*; Ps, *Pisum sativum*; Rc, *Ricinus communis*; Sa, *Sinapis alba*; Ta, *Triticum aestivum*; Se, *Salicornia europaea*; Sl, *Solanum lycopersicum*;So, *Spinacia oleracea*; Ss, *Sequoia sempivirens*; St, *Solanum tuberosum*; Os, *Oryza sativa Indica*.

**Figure 5:**
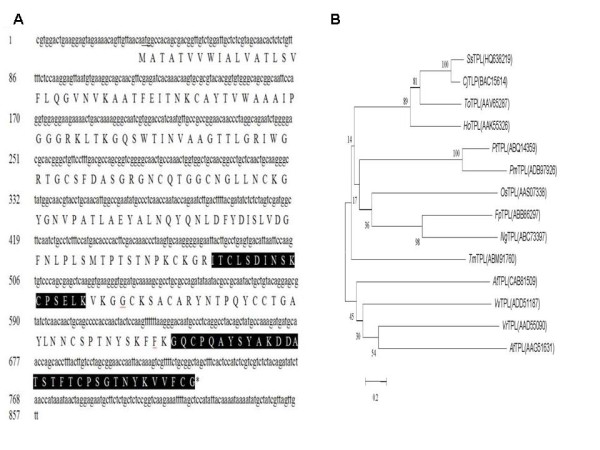
**Sequence coverage and phylogenetic tree of thaumatin-like protein**. (A) Lowercase and capital letters represent the cDNA and amino acid sequence, respectively. Highlighted amino acids indicate the coverage fragments with LC-MS/MS. (B) Cj, *Cryptomeria japonica*; Fp, *Ficus pumila var. awkeotsang *;Ho, *Hordeum vulgare*; Ng, *Nepenthes gracilis*; Os, *Oryza sativa Japonica *group; Pm, *Pinus monticola*; Pt, *Pinus taeda*; To, *Thuja occidentalis*;Tm, Taxus × media; Vr, *Vitis riparia *; Vv, *Vitis berlandieri *x *Vitis riparia*.

**Figure 6:**
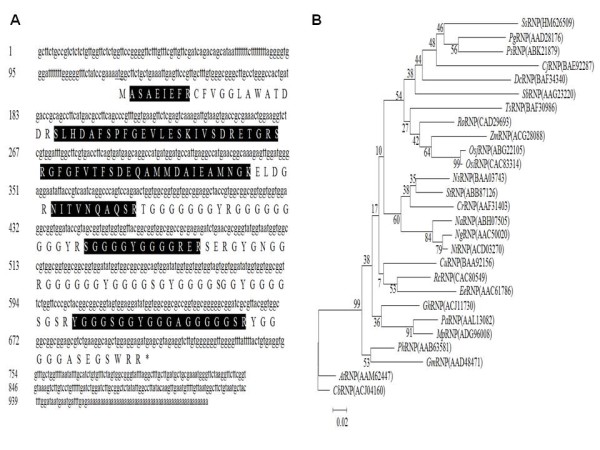
**Sequence coverage and phylogenetic tree of glycine-rich RNA binding protein**. (A) Lowercase and capital letters indicate the cDNA and amino acid sequence, respectively. Highlighted amino acids indicate the coverage fragments with LC-MS/MS. (B) At, *Arabidopsis thaliana*; Cb, *Chorispora bungeana*;Cj, *Cryptomeria japonica*; Cu, *Citrus unshiu*; Cr, *Catharanthus roseus*; Dc, *Dianthus caryophyllus*; Ee, *Euphorbia esula*; Gh, *Gossypium hirsutum*; Gm, *Glycine max*; Mp, *Malus prunifolia*; Na, *Nicotiana attenuata*; Ng, *Nicotiana glutinosa*; Ns, *Nicotiana sylvestris*; Nt, *Nicotiana tabacum*; Osi, *Oryza sativa Indica*; Osj, *Oryza sativa Japonica *Pa, *Prunus avium*; Pg, *Picea glauca*; Ph, *Pelargonium *× *hortorum*; Ps, *Picea sitchensis*; Rc, *Ricinus communis*; Ro, *Rumex obtusifolius*; Sb, *Sorghum bicolor*; Ss, *Sequoia sempivirens*;St, *Solanum tuberosum*; Ta, *Triticum aestivum*; Zm, *Zea mays*.

Our detailed studies were confined to OEE2, RNP and TLP. The deduced amino acid sequences of OEE2, RNP and TLP were shown in Figure 4A,F, Figure 5 and Figure 6A, respectively. These sequences were entered into the database, and an additional database search was performed with in-house Mascot software. More peptides were identified, and the sequence coverage was greatly increased (Table 1 Figure 4 Figure 5 Figure 6). OEE2 was identified in spot 1, with a predicted molecular mass of 27.7 kD. However, the apparent molecular mass of OEE2 on the protein gel was approximately 21 kD, which was much smaller than expected. Additionally, the *N*-terminus peptide fragments of OEE2 were not discovered in this study (Table 1). Altogether, the results suggest that the identified OEE2 is a chloroplast form without the transit peptide. The transit peptide of OEE2 could be cleaved by a protease before translocation into the chloroplast.

**Figure 7:**
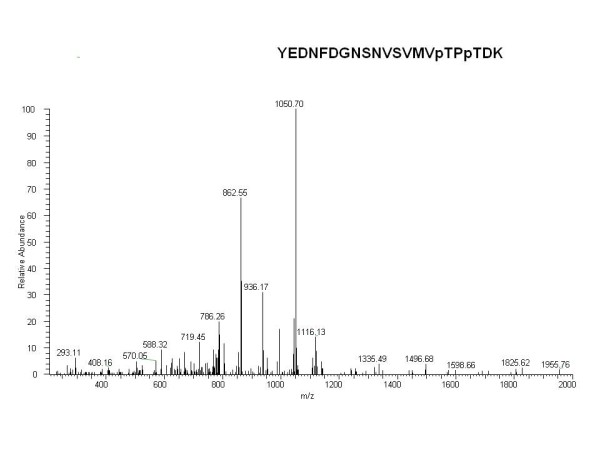
**MS/MS fragmentation pattern of the phosphopeptide of SsOEE2**. The phosphopeptide (YEDNFDGNSNVSVMVpTPpTDK) included two phosphorylation sites, labeled with *p *next to the site.

Without including the *Sequoia *OEE2 protein sequence in the database, only two OEE2 peptides were identified (Table 1). A Mascot protein score of 2017 was obtained when including *Sequoia *OEE2 in the database. Thirteen OEE2 peptides, including a phosphopeptide, were identified. The protein coverage was 52%. The phosphopeptide of OEE2 was doubly phosphorylated (YEDNFDGNSNVSVMVpTPpTDK), and the MS/MS spectrum of the phosphopeptide is shown in Figure 7. This phosphopeptide was validated by Scaffold, with a confidence interval > 95% (Additional file 1).

RNP was identified in spot 3. Without including the RNP protein sequence in the database, only one peptide was identified (Table 1). The Mascot protein score was 67, and the protein coverage was 11%. The Mascot protein score was 1155 when including the *Sequoia *RNP protein sequence in the database. Fourteen peptides were identified, and the protein coverage was 61%. TLP was identified in spot 2. The Mascot protein score was 833, and three peptides were identified.

### Protein levels of OEE2, RNP, and TLP in repeatedly grafted Sequoia shoots

All of the above three proteins were consistently identified in the three biological replicates. OEE2 protein abundance was found to be up-regulated in Rejuvenated76 and Rejuvenated94. The protein level in the adult specimen (Adult76 and Adult94) was lower than the juvenile and rejuvenated samples (Figure 3). Adult76 and Adult94 were significantly different from Juvenile, Rejuvenated76 and Rejuvenated94 (*p *< 0.05; one-way analysis of variance [ANOVA] followed by Duncan's multiple range test for statistical analysis of means) (Additional file 3 &4). The protein abundance of OEE2 through the steps of repeated grafting showed a positive correlation between rejuvenation and protein abundance in *Sequoia *shoot cultures initiated in both 1976 and 1994 (Figure 3).

By contrast, the protein abundance of RNP was found to be up-regulated in Rejuvenated94 but not Rejuvenated76. The protein level of RNP in the adult plantlet (Adult76 and Adult94) was lower than the Juvenile and Rejuvenated94 samples (Figure 3). However, Adult76 and Adult94 were not significantly different from Juvenile, Rejuvenated76 and Rejuvenated94 (*p *< 0.05; one-way ANOVA followed by Duncan's multiple range test for statistical analysis of means) (Additional file 3, 4). Therefore, the protein abundance of RNP through the steps of repeated grafting showed no correlation between rejuvenation and protein abundance in *Sequoia *shoot cultures initiated in 1976 or 1994 (Figure 3).

TLP abundance was found to be down-regulated in Rejuvenated76 and Rejuvenated94. The protein level in the adult specimen (Adult76 and Adult94) was higher than the Juvenile and Rejuvenated samples (Figure 3). Adult76 and Adult94 were significantly different from Juvenile, Rejuvenated76 and Rejuvenated94 (*p *< 0.05; one-way ANOVA followed by Duncan's multiple range test for statistical analysis of means) (Additional file 3, 4). The protein abundance of TLP through the steps of repeated grafting showed a reverse correlation between rejuvenation and protein abundance in *Sequoia *shoot cultures initiated in both 1976 and 1994 (Figure 3).

### Phylogenetic analysis of OEE2, TLP and RNP

The full-length cDNAs of the *SsOEE2*, *SsTLP*, and *SsRNP *genes were cloned from *Sequoia *shoots *in vitro *and further analyzed. The cDNA length of *SsOEE2 *was 981 bps and encoded a predicted 27.7 kDa protein. The isoelectric point of *SsOEE2 *was 9.18. *SsOEE2 *contained a signal peptide for chloroplast in the *N*-terminal. Phylogenetic analysis of OEE2 by the neighbor-joining method revealed that the *Sequoia sempervirens *protein had 49-71% homology in the amino acid sequence with the protein of other species (Figure 4B). The peptide sequence of OEE2, among gymnosperms, was conserved and displayed diversity from those of algae and other angiosperms. The cDNA length of *SsTLP *was 896 bps and encoded a predicted 24.7 kDa protein. The isoelectric point of TLP was 8.62. It contained a secretory signal peptide in the *N*-terminal. The phylogenetic analysis of TLP, based on calculations by the neighbor-joining method, revealed that *Sequoia sempervirens *had 43-71% homology in the amino acid sequence with the other species (Figure 5B). The peptide sequence of TLP was conserved among gymnosperms and angiosperms.

The cDNA length of *SsRNP *was 1123 bps and encoded a predicted 18.5 kDa protein. The isoelectric point of RNP was 8.05 and contained a nuclear localization signal in the *C*-terminal. The phylogenetic analysis of RNP, based on calculations by the neighbor-joining method, revealed that *Sequoia sempervirens *shared 49-79% homology in the amino acid sequence with the other species (Figure 6B). The peptide sequence of RNP, among gymnosperms, was conserved and displayed diversity from those of angiosperms.

### Gene expression patterns of up- or down-regulated genes in repeatedly grafted Sequoia shoots

**Figure 8:**
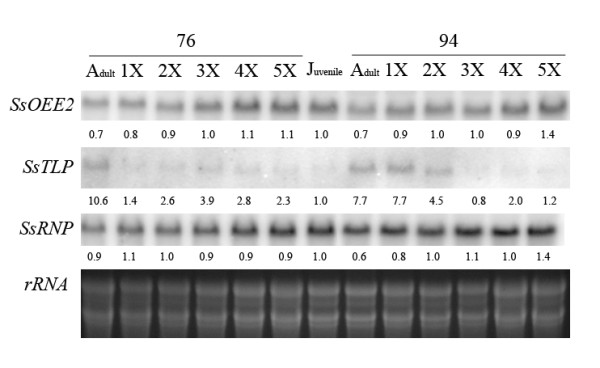
**The expression level of *SsOEE2*, *SsRNP *and *SsTLP *in *Sequoia *shoot during repeated grafting**. 76 and 94 are the *Sequoia *shooting established in 1976 and 1994 respectively. Adult: adult shoot, Juvenile: juvenile shoot 1X~5X: repeated grafting. The expression intensity of *SsOEE2 *, *SsRNP *and *SsTLP *were normalized with total *rRNA *acting as loading control and scoring by comparing to juvenile stage.

To further explore the role of up- or down-regulated genes in phase changes, the expression patterns of *SsOEE2*, *SsRNP*, and *SsTLP *in the two adult shoot cultures, established in 1976 and 1994, respectively, were monitored following repeated grafting (Figure 8). Both *SsOEE2 *and *SsRNP *displayed higher expression levels in the juvenile phase and a lower expression level in the adult phase (Figure 8). Furthermore, their expression levels also steadily increased with the number of repeated grafts. Specifically, a higher expression level of *SsOEE2 *was present in the *Sequoia *shoots during the juvenile and rejuvenated stages, which is consistent with the protein accumulation pattern (Figure 3). However, the expression level of *SsRNP *displayed a similar pattern at different stages in *Sequoia *shoots initiated in 1976 but increased slightly with repeated grafting initiated in 1994.

By contrast, the expression level of *SsTLP *in adult shoots was initially high but progressively decreased with repeated grafting. The expression pattern of *SsTLP *was opposite to *SsOEE2 *and decreased with repeated grafting (Figure 8). The highest expression level of *SsTLP *was present in *Sequoia *shoots during the adult stage and significantly decreased with one-time *Sequoia *grafting initiated in 1976 and the third *Sequoia *grafting initiated in 1994.

## Discussion

Plant maturation involves a sequence of developmental stages or phases that can be categorized as embryo, juvenile, transitional and adult. Aging in perennials is very complex, and no consensus has been achieved in the general concepts related to this topic [25]. A juvenile phase occurs in all woody plants, which lasts up to 30-40 years in certain forest trees, during which flowering does not occur. The physiology and molecular biology aspects are then considered when listing biochemical markers of maturation in woody plants. These markers occur as part of primary and secondary metabolism (e.g., mineral and carbon nutrition, growth regulators, polyamines, phenolic compounds, and peroxidase activity) and gene expression (e.g., nucleic acids, transcription and proteic synthesis) [25-28].

The transition leading to adulthood is accompanied by changes in morphological characteristics, such as a loss of adventitious rooting, modification of leaf morphology, an enervated growth rate, and a variation in phytohormone levels [29-31]. The process of rejuvenation of the adult shoot apex or inflorescence reversion was identical in *planta *and well characterized by research that showed some of the key genes involved in the reversion of determinate floral meristem to indeterminate shoot meristem [32,33]. However, most species are less prone to maturational stage reversion because signals from the leaf are less ephemeral even when its initiation is dependent on environmental cues. Several plants undergo rejuvenation when exposed to optimal stimuli, such as gibberellins [34], cytokinin [35], ABA [36], and grafting[16]. Grafting is known to cause phase reversion in woody perennials (e.g., citrus [37], cupressus [38], eucalyptus [38], hedera [39], passiflora [40], pseudotsuga [41], and avocado [14].

The factors triggering maturation and phase transition and how this process is regulated in terms of changes in gene expression, cellular signaling, and metabolism are well known. Unfortunately, most of the established knowledge on phase transition is based on annual *Arabidopsis *or rice. Much less genetic evidence has been obtained regarding rejuvenation in perennials. We focused on *Sequoia *proteins and genes that displayed an identical pattern at different developmental stages, and advances in proteomics has helped unravel some of the particular features of rejuvenation in *Sequoia*.

### Identification of OEE2 protein

OEE2 is a core component of the PSII complex in the chloroplast in plants [42]. Evidence has shown that OEE2 in higher plants can only associate with the PSII core complex through OEE1 and OEE3. The oxygen-evolving capacity of PSII decreased to as low as 5-10% in the absence of OEE2 [42]. Based on the literature, the accumulation of OEE2 can be regulated by plant hormones. The abundance of OEE2 increased in the cytokinin-treated moss *Physcomitrella patens *[43]. The accumulation of OEE2 can also be regulated by abiotic stress. Up-regulated OEE protein levels have also been detected in *E. elongatum *and Norway spruce during drought stress [44,45] and in rice undergoing salt stress and metal treatment [46]. Furthermore, the accumulation of OEE2 can be regulated by development. The photosynthetic rate and expression level of *OEE *increased in leaves of maize which roots were colonized by *Trichoderma virens *[47].

A decrease in photosynthesis with increased aging has been shown to be a significant feature of several trees [27]. Higher rates of photosynthesis and chlorophyll *a/b *ratios were present during the juvenile and rejuvenate stages, but quantum efficiencies of PSII indicated equivalent effectiveness in the juvenile, rejuvenate, and adult stages. Recent studies have demonstrated RNA interference (RNAi) in PsbP protein, which has greater homology with *SsOEE2*, with similar results in tobacco [48]. Therefore, the increasing expression level of *SsOEE2 *is a critical event for restoring the net photosynthesis rate. Furthermore, the photosynthesis rate and expression level of *OEE *were increased in maize leaves when the roots were colonized by *Trichoderma virens *[47]. Moreover, the plant in the adult stage is much shorter in the juvenile stage (Figure 1). With PsbP protein knockout in tobacco, the transgenic plant phenotype displayed dramatic growth retardation and also decreases in the chlorophyll *a/b *ratio and oxygen evolution [49]. This indicated the effect of roots on sink activity and in directing carbon partitioning toward the roots and promoting their development. Therefore, this indicated that active OEE2 is related to nutrient recomposition associated with restoration of rooting and foliar morphology, which were other significant features of *Sequoia *rejuvenation.

The protein accumulation and gene expression level of *SsOEE2 *increased through successive grafts in the present study (Figure 3 Figure 8). A positive correlation was found between rejuvenation and protein and transcript abundance of the *SsOEE2 *gene. This suggested that *SsOEE2 *may be involved in *Sequoia *rejuvenation. Specifically, based on the molecular mass, the OEE2 identified in this study appeared to be localized in the chloroplast. Altogether, these results suggest that the chloroplast protein may be involved in *Sequoia *rejuvenation. Because only plants possess chloroplast, and the fact that rejuvenation found only in plants implicates chloroplast may be one of the regulatory components in plant rejuvenation. In addition, expression of a chlorophyll a/b binding gene was also found to be greater in developing juvenile than in mature foliage [50,51]. It is possible that chloroplast proteins are a key of rejuvenation process in plants. Whether photosynthesis efficiency is altered in *Sequoia *rejuvenation requires further studies.

### Identification of RNP protein

Plant RNP is a class of genes with diverse functions, and the functions of many of these genes are still unknown. The expression of *RNP *genes is regulated by abiotic stress (e.g., cold, wounds, and hormones such as auxin and abscisic acid [52]. In *Arabidopsis*, *AtGRP7 *has been widely studied. *AtGRP7 *has been reported to promote phase transition in *Arabidopsis *and is recognized as a novel autonomous pathway component [53]. However, transgenic plants overexpressing *AtGRP7 *and wildtypes using the ATH1 GeneChip did not show significant changes in *FCA *or *FY *under a long-day photoperiod. *FLC *level was lowered in *AtGRP7OX *plants. This indicated that RNP may play a multifunctional role in different phase transitions in *planta*.

Many types of RNA-binding proteins (RBPs) play diverse roles in regulating RNA metabolism in a variety of cellular processes [54]. While analyzing the cellular localization of two rice OsRZ2-GFP and OsRZ3-GFP fusion proteins using a confocal microscope, strong GFP signals were observed in the nuclei of roots of *OsRZ2*- and *OsRZ3*-expressing *Arabidopsis *plants [55]. Moreover, the growth of roots in *OsRZ2-*overexpressing grp7 plants was higher than wildtype and grp7 plants, demonstrating that RNPs influence root growth in *Arabidopsis *plants [56,57]. The restoration of rooting competence is an obvious correlative characteristic of *Sequoia *rejuvenation (Figure 1). In Figure 6*SsRNP *displayed greater homology in the amino acid sequence with *AtGRP7 *and demonstrated a potential role RNPs in root growth.

Although the expression level of *SsRNP *in the steps of repeated grafting in the present study suggested no correlation between rejuvenation and protein/transcript abundance of the *SsRNP *gene in *Sequoia *shoot cultures initiated in 1976 or 1994, *SsRNP *still appeared to play a possible role in the restoration of rooting competence during rejuvenation. A previous study showed that OEE2 can be phosphorylated by a RNP-mediated wall-associated kinase [58]. Additionally, a phosphopeptide of OEE2 was identified in this study (Table 1). Whether the phosphorylation of OEE2 is involved in the rejuvenation of *Sequoia *and whether RNP and OEE2 are coordinated during rejuvenation require further studies.

Glycine-rich protein (GRP) has been shown to be abundant in the xylem cell wall and participate in wood formation [59-63]. GRPS were also shown to play an important role in cold adaptation in a biosphere [57,60,62,64]. Therefore, a higher expression level of GRP was present in the later wood, consisting of cells with smaller lumens and thicker cell walls during the chill season [59,60]. Glycine-rich RNA binding proteins (RNP) belong to the Class IV GRPs and are involved in stress adaptation [53,54,65,66]. The expression level of *SsRNP *in *Sequoia *shoot cultures initiated in 1976 displayed a similar expression level during the adult, juvenile, and rejuvenile stages but exhibited slightly increased levels during the juvenile and rejuvenile stages compared with the adult stage in shoots initiated in 1994 (Figure 8).

### Identification of thaumatin-like protein

Thaumatin-like proteins (TLP) are a class of superfamily proteins participating in several biological processing [67]. The expression level of *SsTLP *was higher in the adult stage and decreased with successive grafts. The expression level showed an opposite pattern with *SsOEE2*. TLP is well known to belong to pathogenesis-related (PR) protein family 5 (PR5) [68]. Most TLPs have strong antifungal activity [69-71], but some TLPs have little or no antifungal activity [72,73]. *SsTLP *displayed high homology with two TLPs--*Cryj 3.1 *and *Cryj 3.2 *isolated from *Cryptomeria japonica *[74]. Several TLPs isolated from *Cryptomeria japonica *have different expression patterns in plant tissue, with a higher expression level of *Cryj 3.1 *in the reproductive organ. Furthermore, *gSN-TLP *isolated from elderberry trees (*Sambucus nigra *L.) also displayed higher expression levels in older leaves compared with younger leaves [75]. These studies indicate that TLPs could be an adult trait and worthy of future investigations of their function in rejuvenation.

## Conclusions

In the present study, three proteins differentially accumulated during different rejuvenation stages in *Sequoia sempervirens*. These included OEE2, TLP and RNP. The protein abundance and transcript abundance of a phosphoprotein SsOEE2 were consistently lower in *Sequoia *shoots during the adult stage but increased with repeated grafting. This correlation between rejuvenation and protein and transcript abundance of the genes suggested that *SsOEE2 *is associated with rejuvenation, and may be involved in *Sequoia sempervirens *rejuvenation.

## Materials and methods

### Tissue culture

Continuously cultured *Sequioa sempervirens *(D. Don) Endl. shoots from freshly germinated seedlings served as the juvenile rootstocks, and those from mature trees provided the adult shoot meristems. The continuous cultures were initiated from terminals of juvenile and adult shoots and from scion-growth remnants following regrafting of shoot tips. One centimeter long terminals were subcultured at 6 week intervals in nutrient media containing Murashige and Skoog salts [76], 3% sucrose, 0.2% gelrite, 555 μM myo-inositol, 3 μM thiamine HCl, 2.4 μM pyridoxine HCl, 4.1 μM nicotinic acid, and 26.6 μM glycine. Grafting was performed by inserting the obliquely cut base of a 1.5 cm long shoot terminal into a longitudinal incision made in a rooted 1 cm tall juvenile stem segment. Regrafting was performed at 8 week intervals. Shoot cultures and grafted plants were maintained at 27°C with 16 h daily exposure to 22.5 μmol m^-2 ^s^-1 ^cool-white fluorescent light.

### Plant materials

*Sequoia *shoots at different developmental stages or phases were cultured as previously described [16]. The adult shoot stocks were established by culturing shoot tips excised in 1976 and 1994. Cultures of juvenile shoot cuttings maintained as shoot stocks were established using fresh *in vitro *germinated seeding. Rejuvenated shoots were obtained by grafting the shoot tips five times from the two adult *Sequoia *shoots by grafting the adult shoot tips onto juvenile rootstocks. After grafting, shoots were severed and maintained in stocks by subculturing 2-3 cm terminal sections every 2 months in MS-based medium [76].

### Protein extraction

This method was modified from a previous study [77]. Two grams of the plant sample were ground with a mortar and pestle in liquid nitrogen. 0.15-0.2 g of the sample was transferred to 10 Eppendorff centrifuge tubes. Each tube was washed with a 10-fold volume of cold 10% trichloroacetic acid/acetone containing, successively, 0.07% 2-mercaptoethanol, cold 80% methanol containing 0.1 M ammonium acetate and 0.07% 2-mercaptoethanol, and cold 80% acetone containing 0.07% 2-mercaptoethanol and cold 100% acetone. After each wash, all tubes were centrifuged at 16,000 × *g *at 4°C for 5 min, and the supernatant was discarded. When all washes were finished, the pellets were air dried at room temperature for at least 10 min, and 0.8 mL 0.1 g^-1 ^of a starting sample of 1:1 phenol/sodium dodecyl sulfate (SDS) buffer was added, mixed thoroughly, incubated for 10 min, and centrifuged at 16,000 × *g *at 4°C for 10 min. The phenol phase was pipetted into a 50 ml falcon tube. At least 3-5 volumes of cold methanol containing 0.1 M ammonium acetate and 0.07% 2-mercaptoethanol were added. The tube was stored at -20°C overnight. The falcon tube was centrifuged at 3,000 × *g *at 4°C for 10 min the next day. The precipitates were washed twice with cold methanol containing 0.07% 2-mercaptoethanol and twice with 80% cold acetone containing 0.07% 2-mercaptoethanol. Following each washing step, the falcon tube was centrifuged at 3,000 × *g *at 4°C for 10 min. Finally, the pellet was resuspended with 16 mL cold 100% acetone and aliquoted into eight 2.0 mL Eppendorff centrifuge tubes. These tubes were then centrifuged at 16,000 × *g *at 4°C for 5 min, and the supernatant was discarded. The pellet was air dried at room temperature for at least 10 min and dissolved in 200 μL rehydration buffer (6 M urea, 2 M thiourea, 0.5% triton X-100, and a trace of bromophenol blue), and the tubes were stored at -80°C overnight.

### Isoelectric focusing and two-dimensional gel electrophoresis

A 1.5 mL Eppendorff centrifuge tube was prepared. An appropriate volume of protein sample containing 800 μg of protein was mixed with 3.8 mg dithiothreitol (DTT) and 0.5 μL ampholyte in the tube. Rehydration buffer was added to achieve a final volume of 500 μL. The protein sample was loaded onto the focusing tray and covered with a 17 cm, pH 3-10, immobilized pH gradient (IPG) strip (linear). Active rehydration was performed on a Bio-Rad Protean IEF cell under 50 voltage for 14 h. Isoelectric focusing was performed on the same machine using the following program: 250 V, linear ramp for 20 min; 10,000 V, linear ramp for 3 h; and 10,000 V for a total of 60,000 Vh. Strips were stored at -80°C overnight. The strips were thawed, and equilibration began with SDS equilibration buffer (50 mM Tris-HCl, pH 8.8, 6 M urea, 30% glycerol, 2% SDS, and a trace of bromophenol blue) the following day. The strips were kept in equilibration buffer 1 (SDS equilibration buffer containing 1% DTT) and shaken for 15-20 min. The strips were moved into equilibration buffer 2 (SDS equilibration buffer containing 2.5% iodoacetamide) for 15-20 min. The strips were then layered on 12% acrylamide gel and embedded in the place of agarose. Electrophoresis was performed on a Bio-Rad Protean II xi Cell (step 1: 32 mA for 30 min; step 2: 90 mA for 270 min) and stopped when the indicated blue line reached the bottom of the gel. Gels were then washed with distilled and deionized water once for 5 min and fixed with fixative buffer (50% methanol and 7% acetic acid) overnight. Gels were washed three times with ddH_2_O (10 min each), and then the stained gels were stained with Sypro Ruby for 4-5 h. Stained gel images were scanned using a GE Typhoon 9400. Gel images were compared using SameSpots (Progenesis, UK) software.

### In-gel digestion of proteins

Two-dimensional gel spots were excised with cut tips individually. Cut gels were soaked in 50 mM dithioerythreitol (DTE)/25 mM ammonium bicarbonate (pH 8.5) at 37°C for 1 h and soaked in 100 mM iodoacetamide/25 mM ammonium bicarbonate (pH 8.5) at room temperature for 1 h in the dark. Gel samples were then soaked in 50% acetonitrile/25 mM ammonium bicarbonate buffer (pH 8.5) three times (15 min each), followed by centrifugation at 10,000 × *g *for 1 min each. Gel samples were soaked with 100% acetonitrile for 5 min. After removing acetonitrile, gel samples were dried in a Speed Vac for approximately 10 min. One hundred nanograms of trypsin and 40 μL 25 mM ammonium bicarbonate were added to each gel sample and incubated at 37°C for at least 16 h. Fifty μL of 50% acetonitrile/5% trifluoroacetic acid (TFA) was added to each gel sample and sonicated for 10 min. The above step was repeated, combined peptide solutions was dried in a Speed Vac for 90 min. Dried peptide pellets were prepared for liquid chromatography-MS/MS (LC-MS/MS) using Mass Solutions Technology.

### Mass spectrometry analyses and protein identification

Peptide samples were analyzed by LC/MS/MS using Q-TOF (Waters, UK) or LTQ Velos (Thermo, US). Peak list files were generated from RAW files. Protein identification was performed using in-house Mascot software (Matrixscience, US) [78]. The mass peak list data were searched against the National Center for Biotechnology Information database using green plant taxonomy. For the Q-TOF dataset, mass tolerance was set to 0.6 D. For the LTQ dataset, mass tolerance was set to 1 D. The missed cleavage site was set to 1. To validate the identified peptides or proteins, Scaffold software (Proteome Software, US) was used to provide confidence interval percentages for the identification of peptides and proteins.

### Statistical analyses of gel spot intensity

Statistical analyses were performed using SPSS 16.0 software (SPSS, Chicago, IL, USA) to compare the protein abundance of the gel spots. Significant effects (*p *< 0.05) in the one-way ANOVA were followed by Duncan's multiple range test.

### RNA extraction

One gram of sample was ground in the mortar treated with liquid nitrogen. The sample powder was mixed with 10 mL extraction buffer (10 g polyvinylpyrrolidone, 10 g hexadecyltrimethylammonium bromide, 4.653 g ethylenediaminetetraacetic acid [EDTA], 58.44 g sodium chloride, 0.25 g spermidine, and fresh 200 μl β-mercaptoethanol). After heating the slurry at 65°C in a water bath for 10 min, delaminating was performed by 13,000 rpm centrifugation at 25°C for 15 min. The supernatant was mixed well with chloroform and isoamylalcohol twice, and the total RNA was collected after precipitation with a 1/4-fold volume of 10 M lithium chloride at 4°C overnight.

### Rapid amplification of cDNA end and cloning of gene of interest

Total RNA was dephosphorylated by alkaline phosphatase and then decapped by acid pyrophosphatase. The completed mRNA was ligated using a GeneRacer RNA oligo (Invitrogen, Carlsbad, California, USA), and cDNA was formed by reverse transcription. The cDNA was used as the template for cloning the genes of interest using the following steps: denaturation at 94°C for 2 min, followed by 20 cycles of amplification (i.e., 94°C for 30 s for denaturation, 47-66°C for 30 s for annealing, depending on the genes, 72°C for 30 s for elongation) and extension at 72°C for 10 min. For cloning OEE2, the primers were OEE25primer (5-CGCGCATAACACTCGGAGGA-3) and OEE25primer (5-CGAAACCCTAAAATGTGCTT-3). For cloning TLP, the primer are TLP5-primer:5-CGTGGACTGAAGGAGTAGAA-3 and TLP-3primer:5-AACAACTAACGATAGCATATTTTATTTTGTAA-3.For cloning RNP, the primers were RNP5primer (5-GGAGTAGAAAGACTGGAGCA-3) and RNP3primer (5-CTCAAATCATTCATTATCCA-3). The amplified DNA fragments of each candidate gene were cloned in pGEM-Teasy vector (Promega, Madison, WI, USA), and the sequences were identified. The confirmed gene sequences were deposited in GenBank with the accession number assigned.

### Northern blotting

The cDNA fragments were randomly labeled with P^32^-dCTP (Rediprime II Kit, Amersham) as the probe to hybridize the RNA-blotted membranes, and the membranes were washed following the standard protocol. The membrane was exposed to a Xuorescent plate for 12 h (Typhoon 9400, Amersham). Band intensities were normalized with respect to the amount of loaded mRNA.

## Abbreviations

DCMU: 3-(3,4-dichlorophenyl)-1,1-dimethylurea; DTE: dithioerythreitol; DTT: dithiothreitol; EDTA: ethylenediaminetetraacetic acid; GRP: glycine-rich protein; IPG: immobilized pH gradients; LC-MS/MS: liquid chromatography-mass spectrometry/mass spectrometry; OEE2: oxygen-evolving enhancer protein 2; Q-TOF: Quatropde-Time of Flight; RBP: RNA-binding protein; RNP: glycine-rich RNA-binding protein; SDS: sodium dodecyl sulfate; TFA: trifluoroacetic acid; TLP: thaumatin-like protein.

## Competing interests

The authors declare that they have no competing interests.

## Authors' contributions

IFC, TJH, and YWH performed 2DE and protein identification. IFC participated in the overall experimental design and quality control of MS data. TJH and YWH performed the protein isolation, two-dimensional gel electrophoresis, MS analyses, and protein identification. CHS and PJC performed the RACE of the genes of interest and Northern blotting. BLH, CIK, and LCH performed the sample preparation. KWY coordinated the preparation of the final manuscript. All authors read and approved the final manuscript.

## Supplementary Material

Additional file 1 **Validation of protein identification using Scaffold software**. This file provided the confidence evidence of the identification of each protein using Scaffold software. Click here for file

Additional file 2 **Identification of other proteins from two-dimensional gel**. Proteins not consistently identified among different Sequoia gel spots were listed in this table. Click here for file

Additional file 3 **Protein abundance of spots on the 2 D gel**. RAW data of protein abundance of spot 1, 2, and 3 in each Sequoia samples. Click here for file

Additional file 4 **ANOVA and Duncan's multiple range test for the protein abundance of spots on the 2 D gel**. RAW data of ANOVA and Duncan's MRT analyses carried out using SPSS software (Ver. 16.0). A: OEE2; B:RNP; C:Thaumatin-like protein. 1:Adult76; 2:Adult94; 3:Juvenile; 4:Rejuvenated76; 5:Rejuvenated94. Click here for file
