# Supplementary material for: Proteomic profiling of proteins associated with the rejuvenation of Sequoia sempervirens (D. Don) Endl
Source: Proteome Sci. 2010 Dec 10;8:64. doi: 10.1186/1477-5956-8-64 (PMC3022872; doi:10.1186/1477-5956-8-64)

## Slide 1
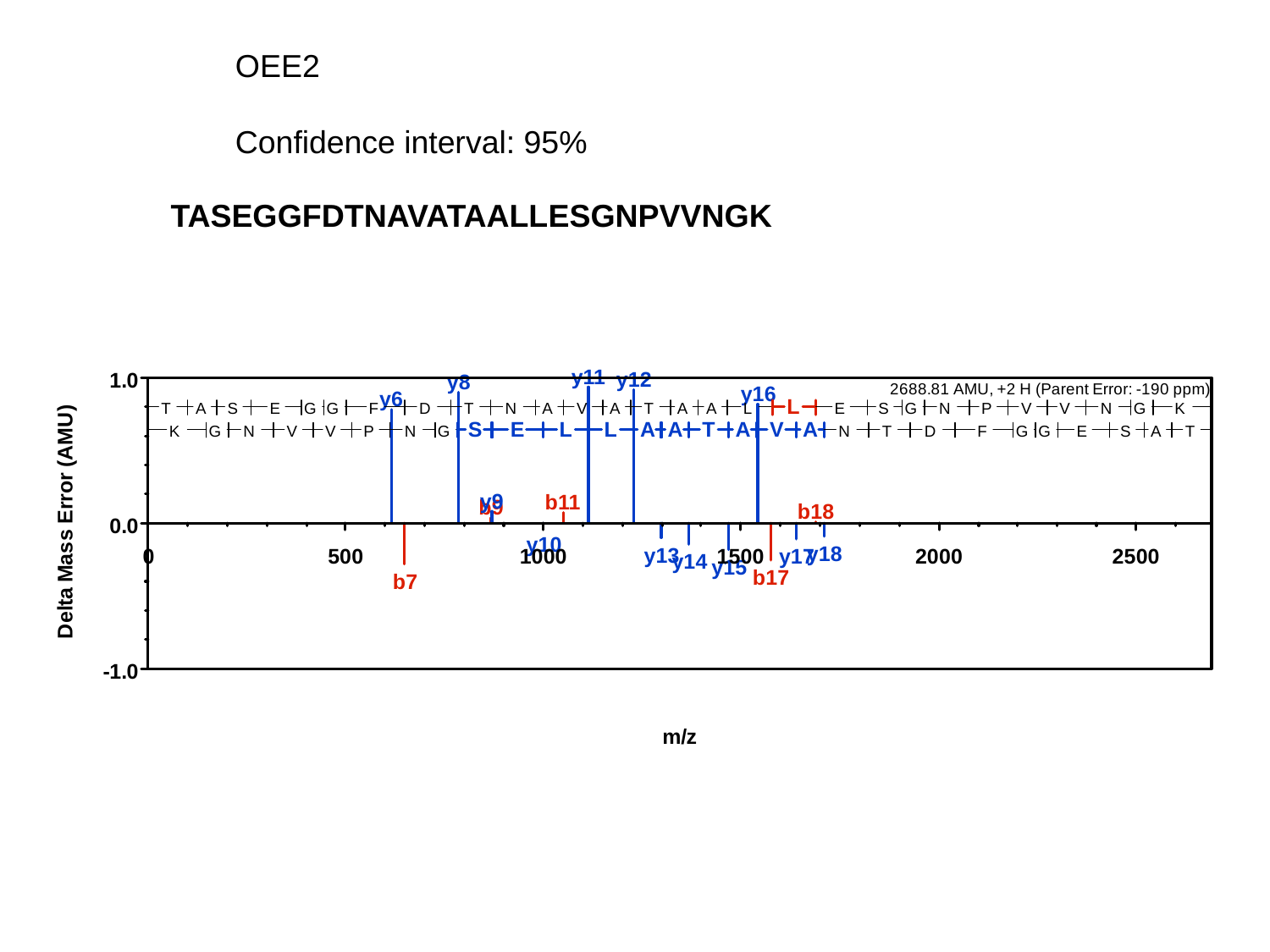

OEE2
Confidence interval: 95%
TASEGGFDTNAVATAALLESGNPVVNGK

## Slide 2
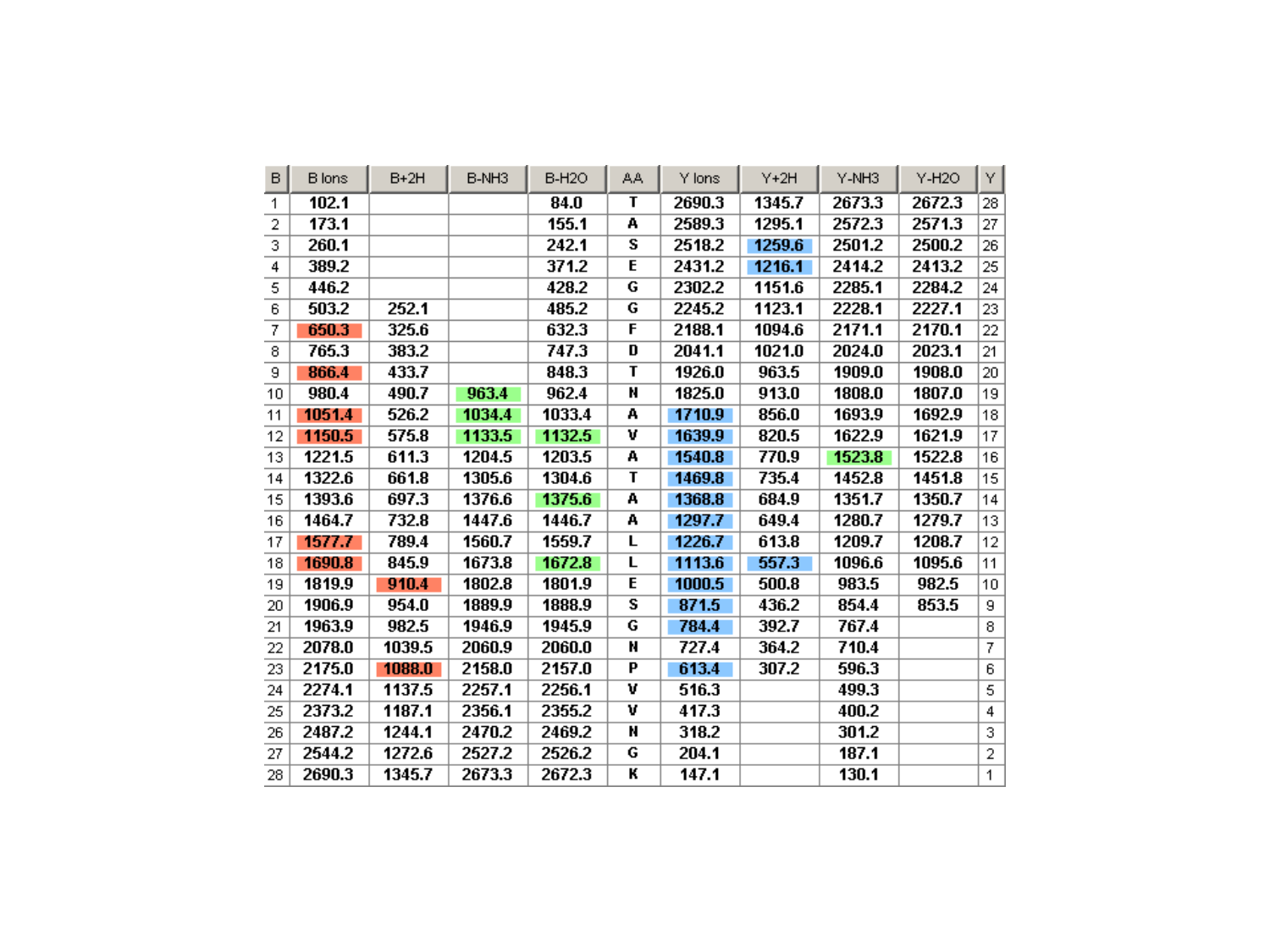

## Slide 3
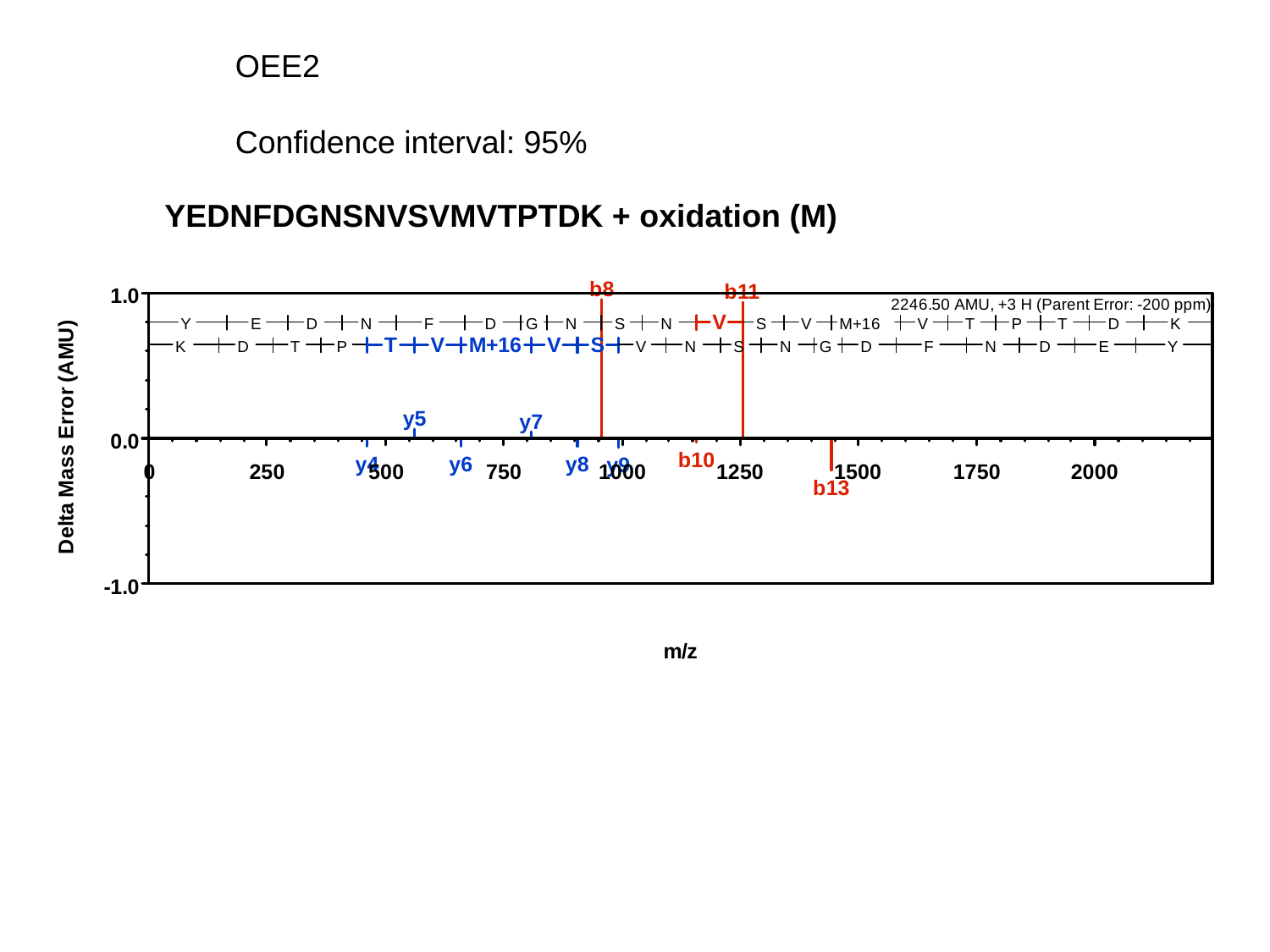

OEE2
Confidence interval: 95%
YEDNFDGNSNVSVMVTPTDK + oxidation (M)

## Slide 4
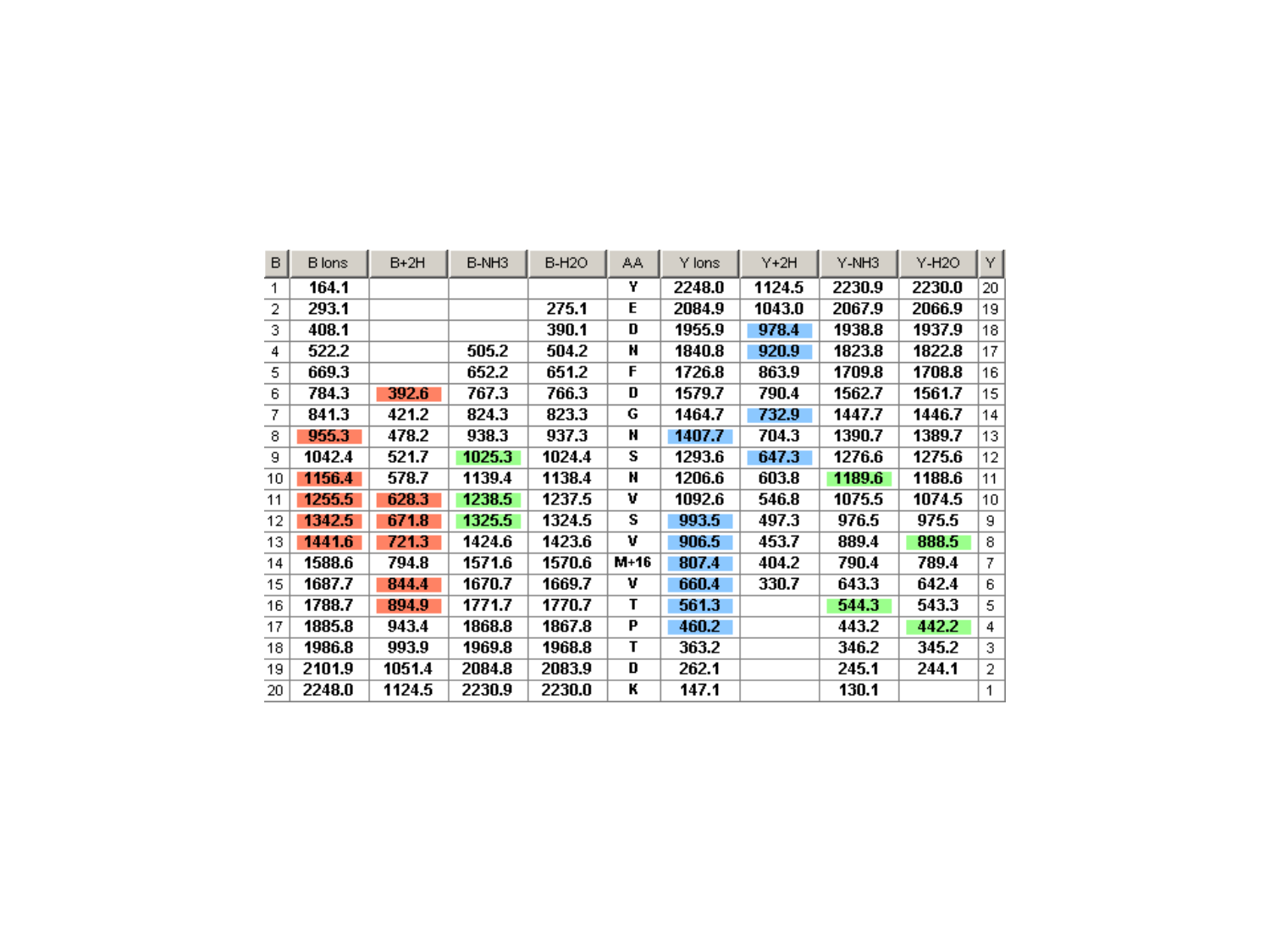

## Slide 5
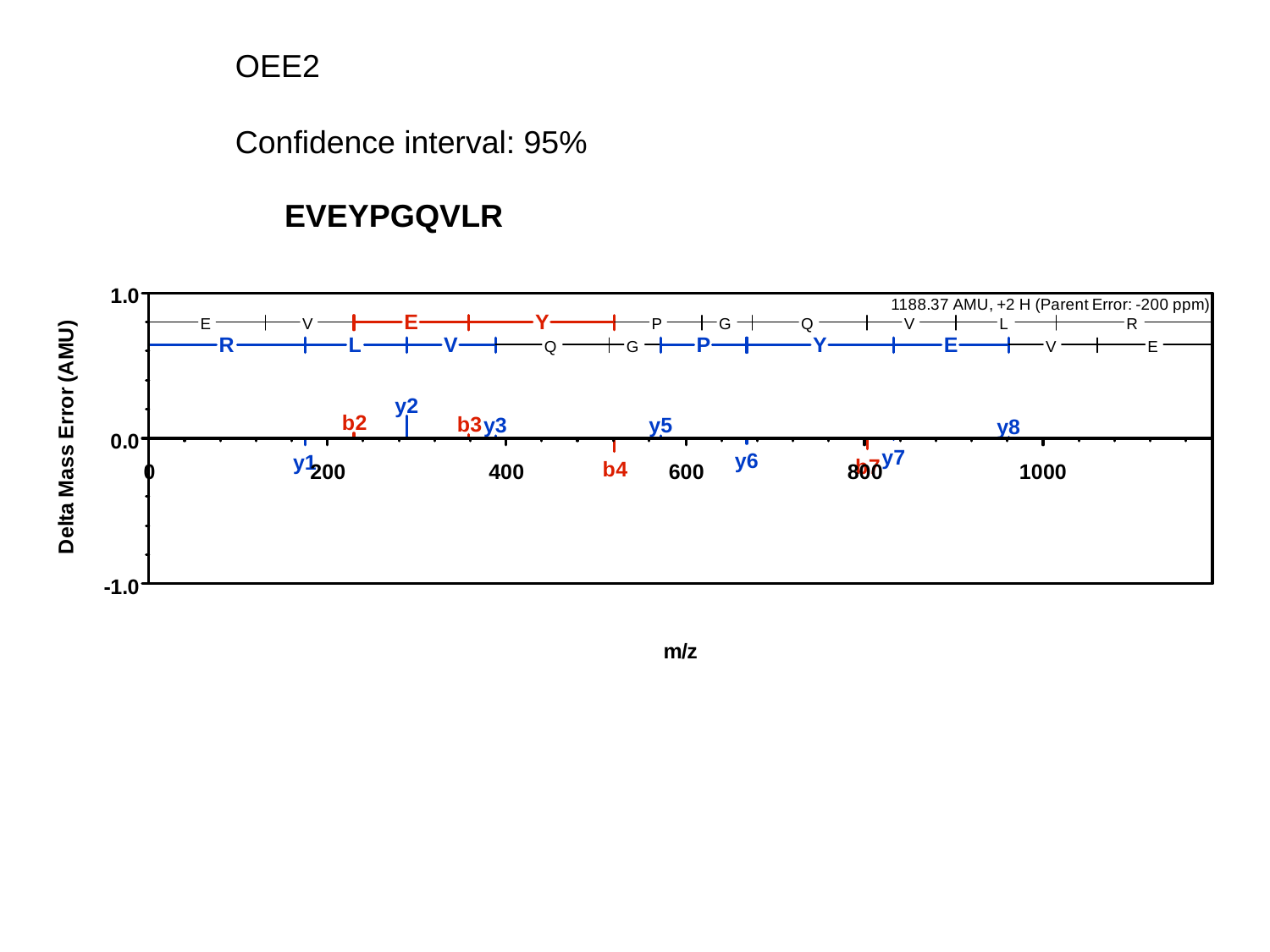

OEE2
Confidence interval: 95%
EVEYPGQVLR

## Slide 6
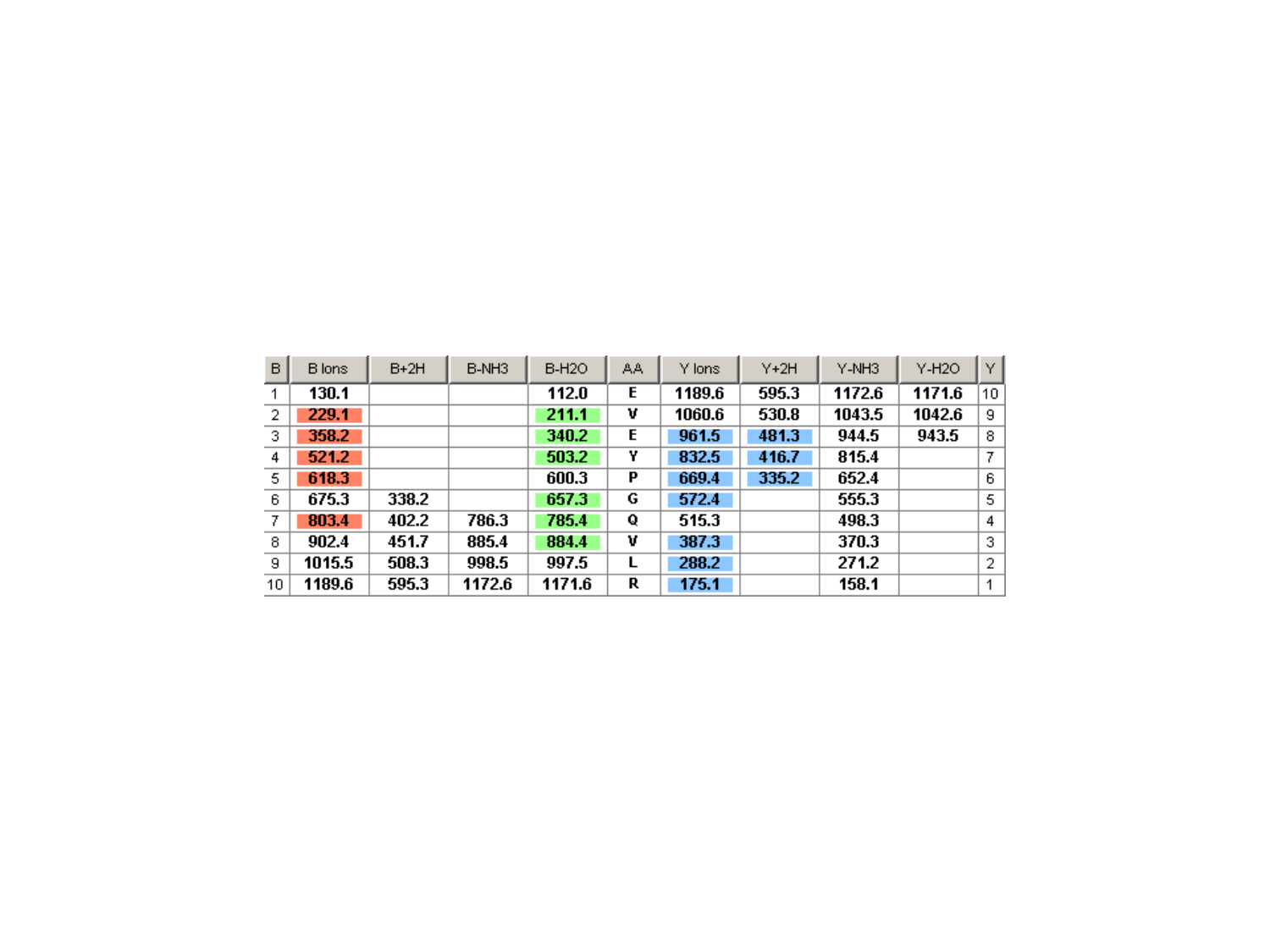

## Slide 7
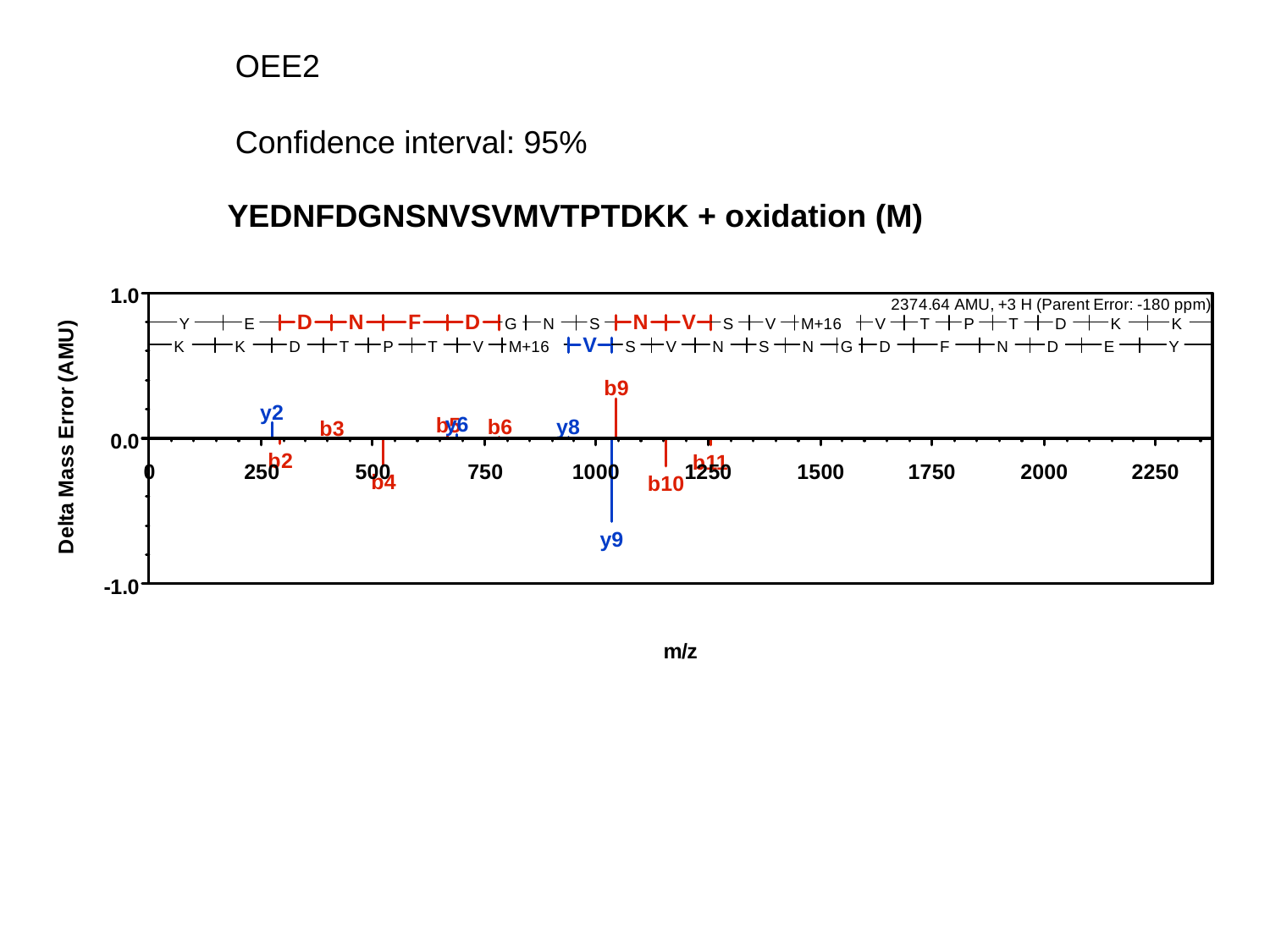

OEE2
Confidence interval: 95%
YEDNFDGNSNVSVMVTPTDKK + oxidation (M)

## Slide 8
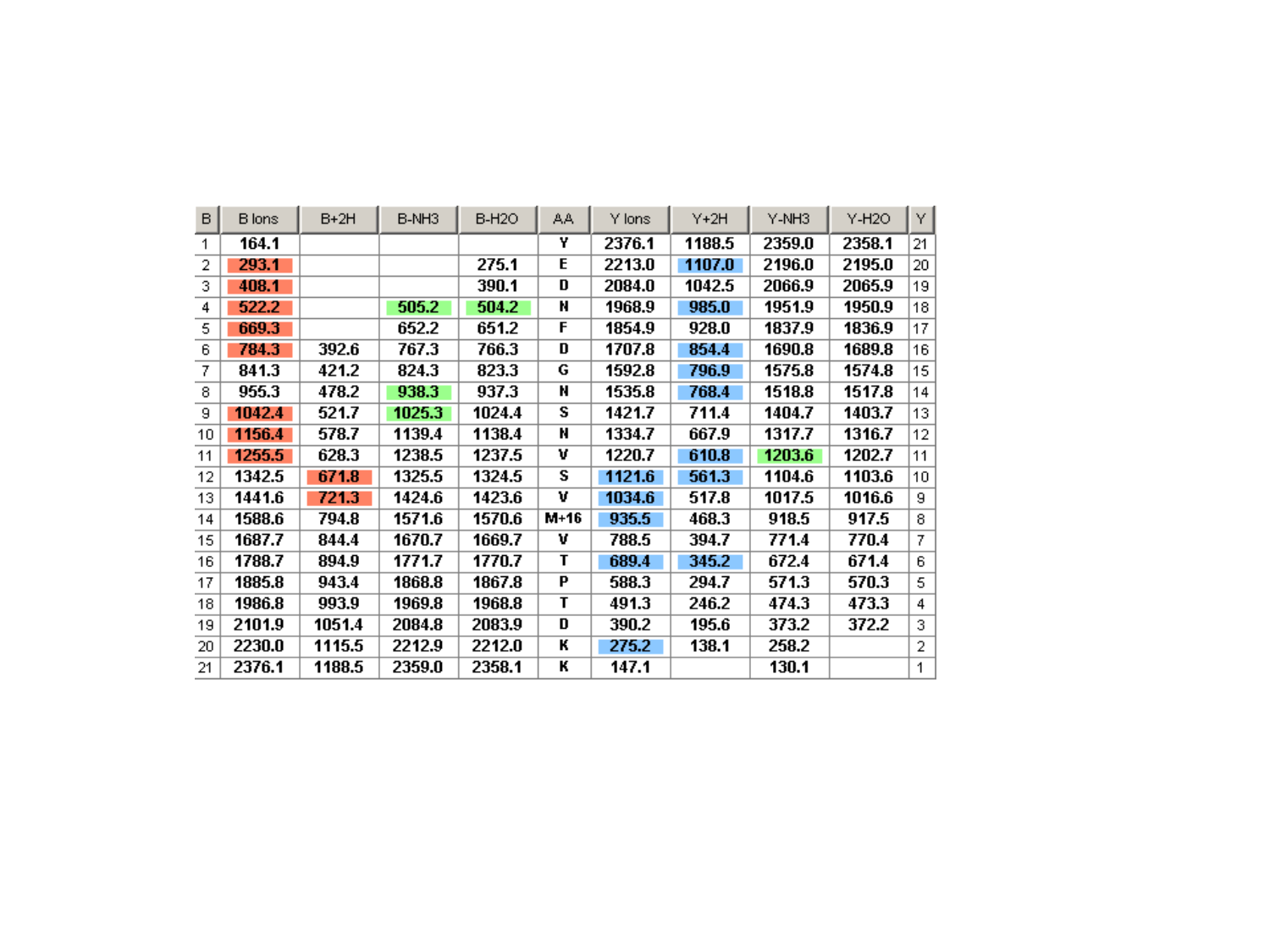

## Slide 9
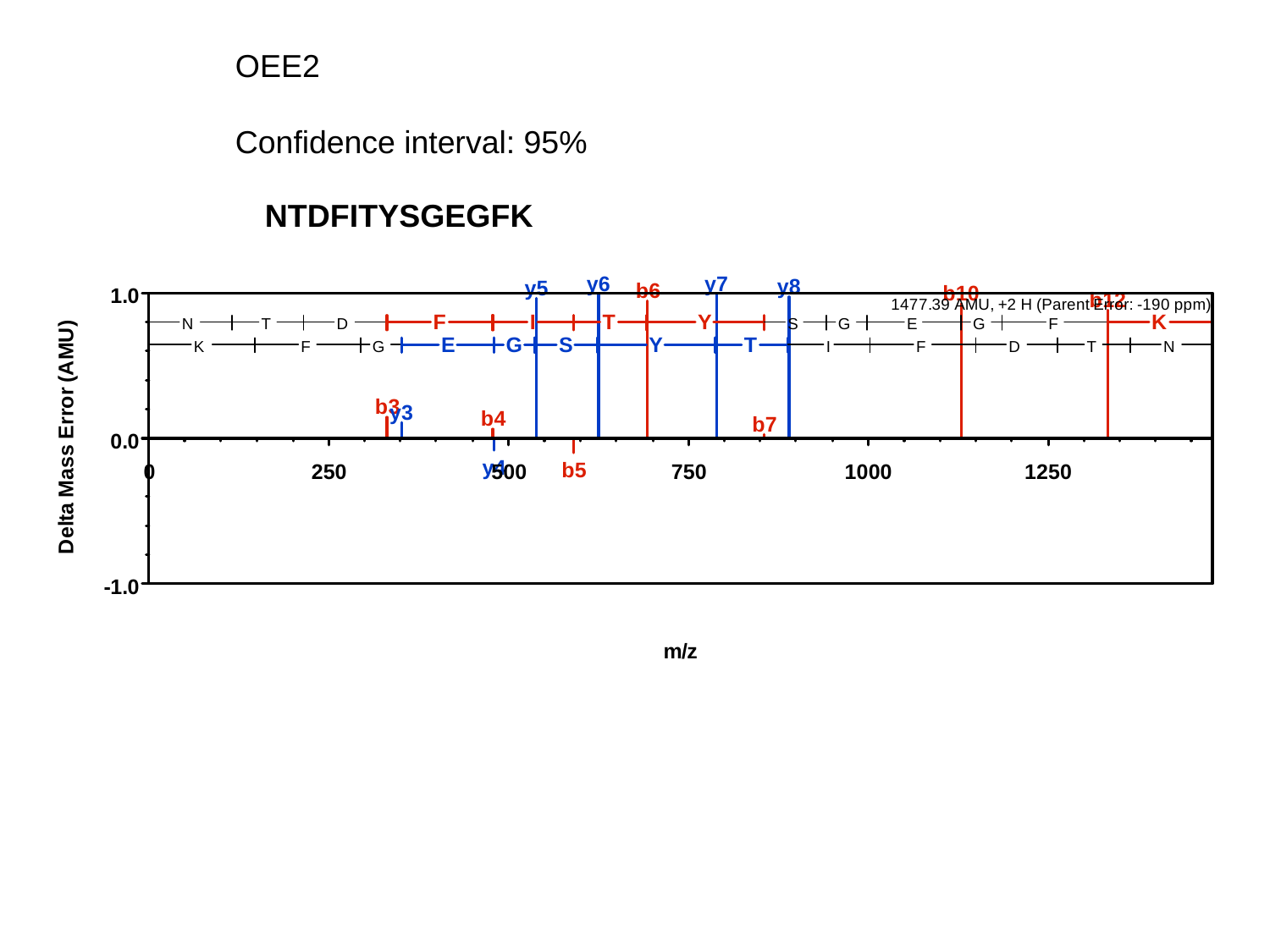

OEE2
Confidence interval: 95%
NTDFITYSGEGFK

## Slide 10
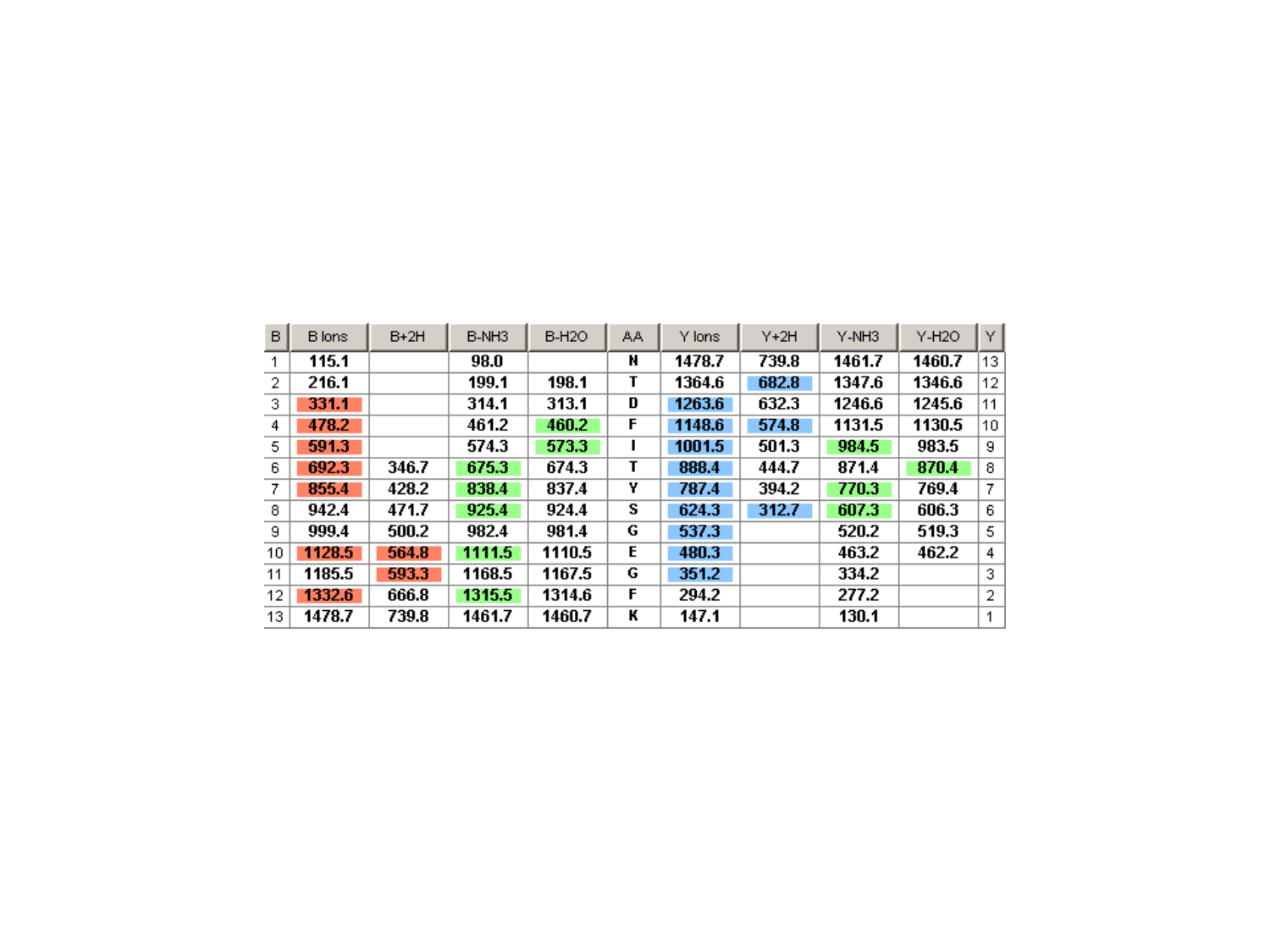

## Slide 11
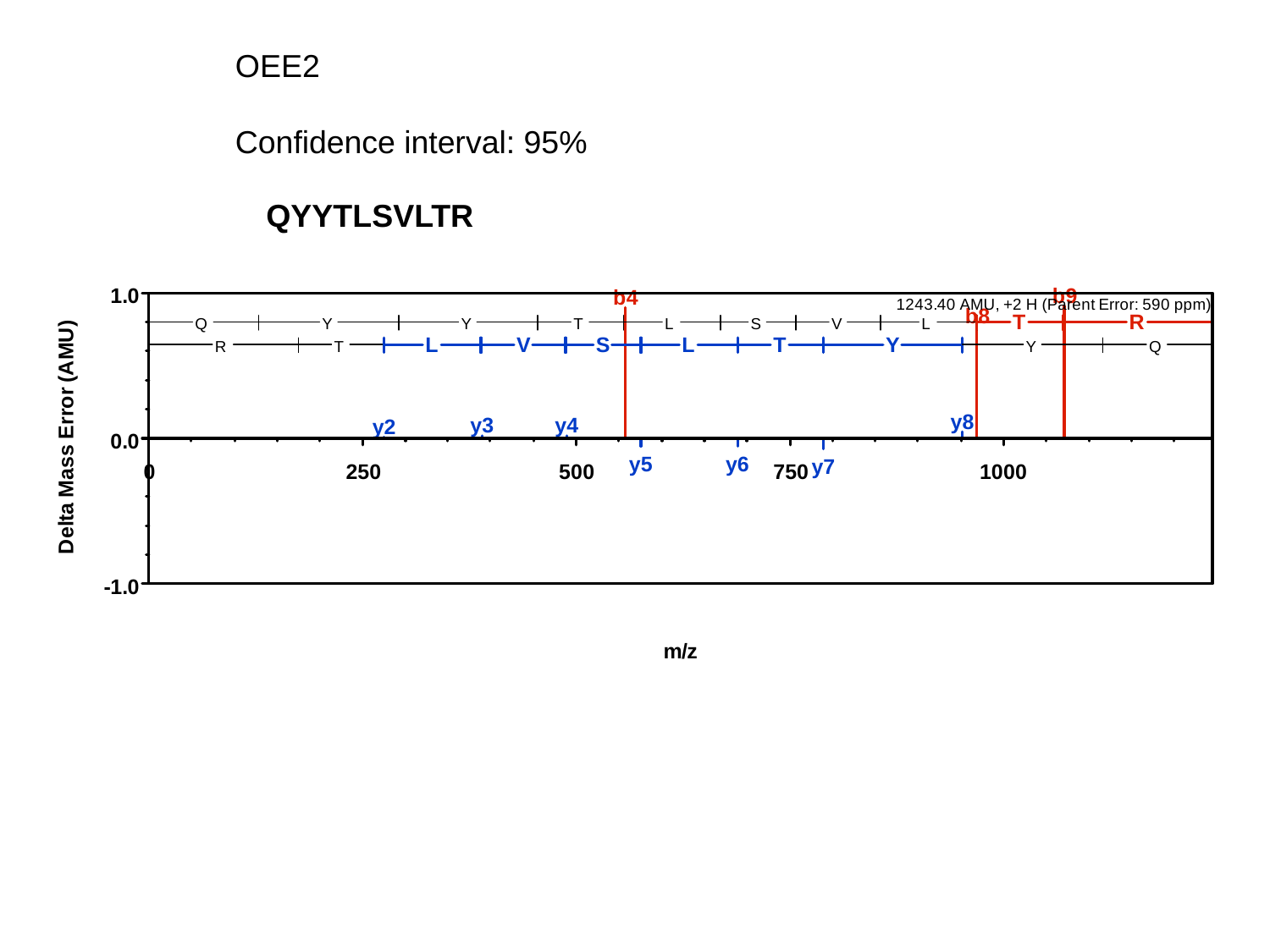

OEE2
Confidence interval: 95%
QYYTLSVLTR

## Slide 12
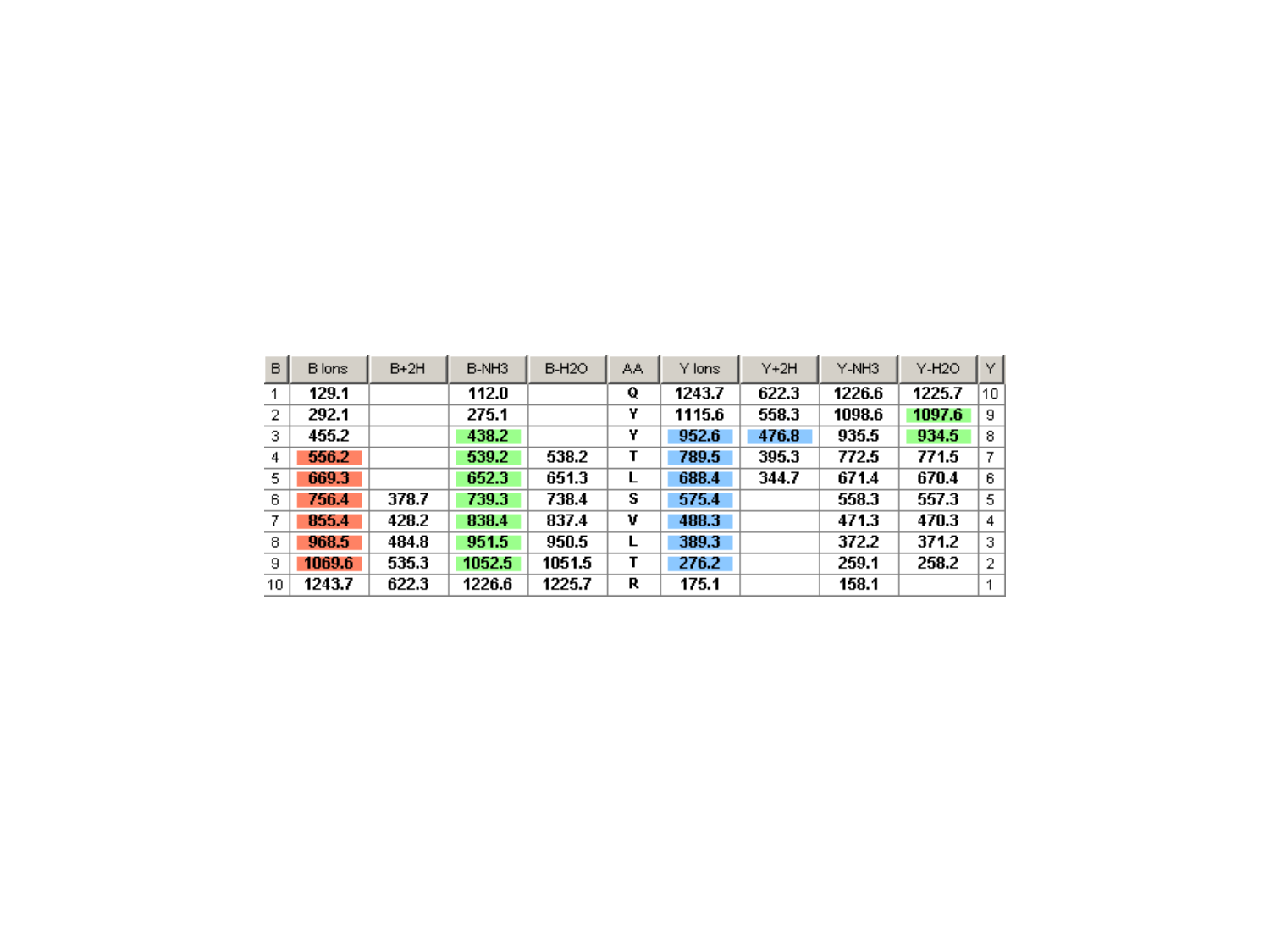

## Slide 13
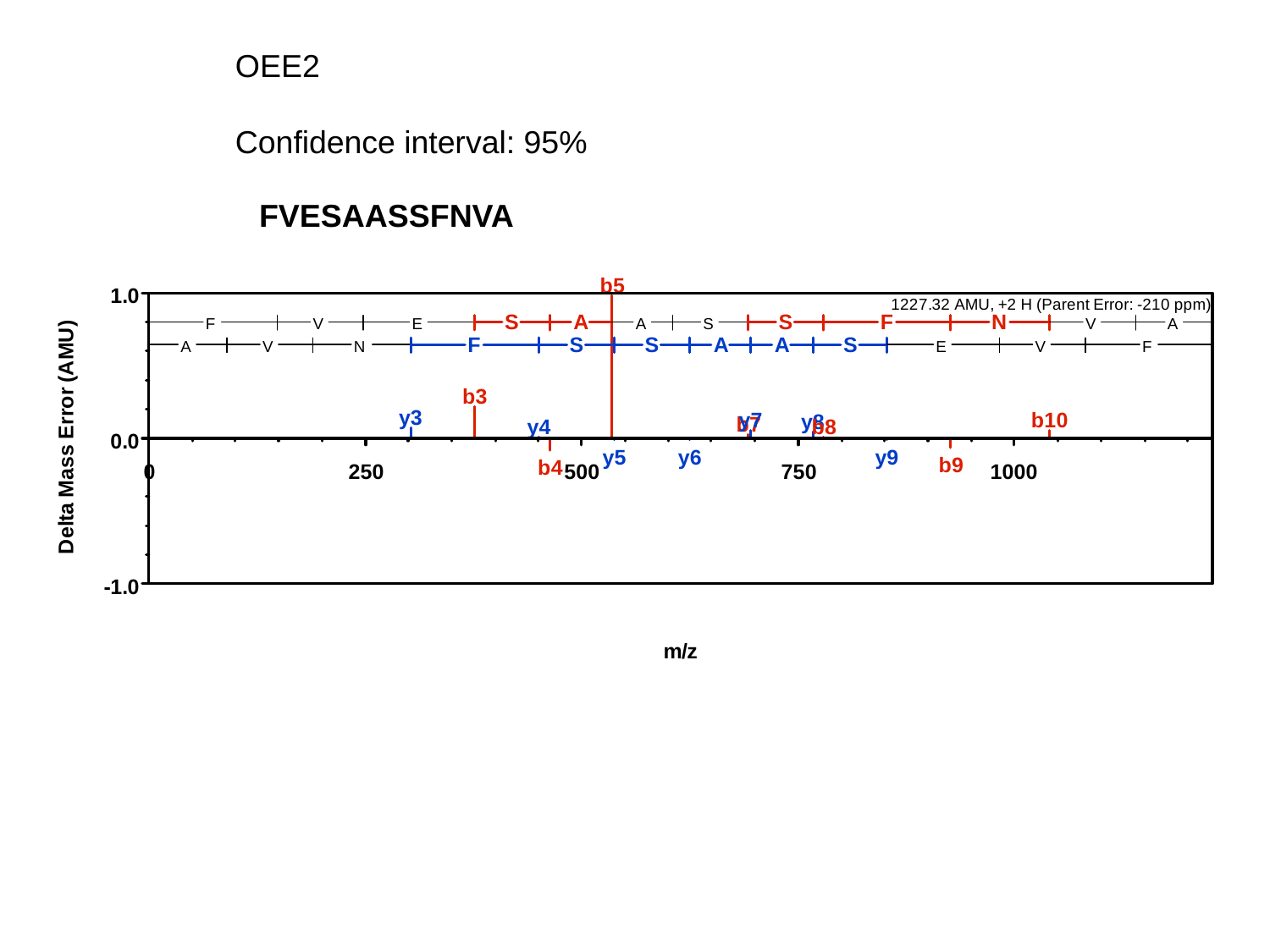

OEE2
Confidence interval: 95%
FVESAASSFNVA

## Slide 14
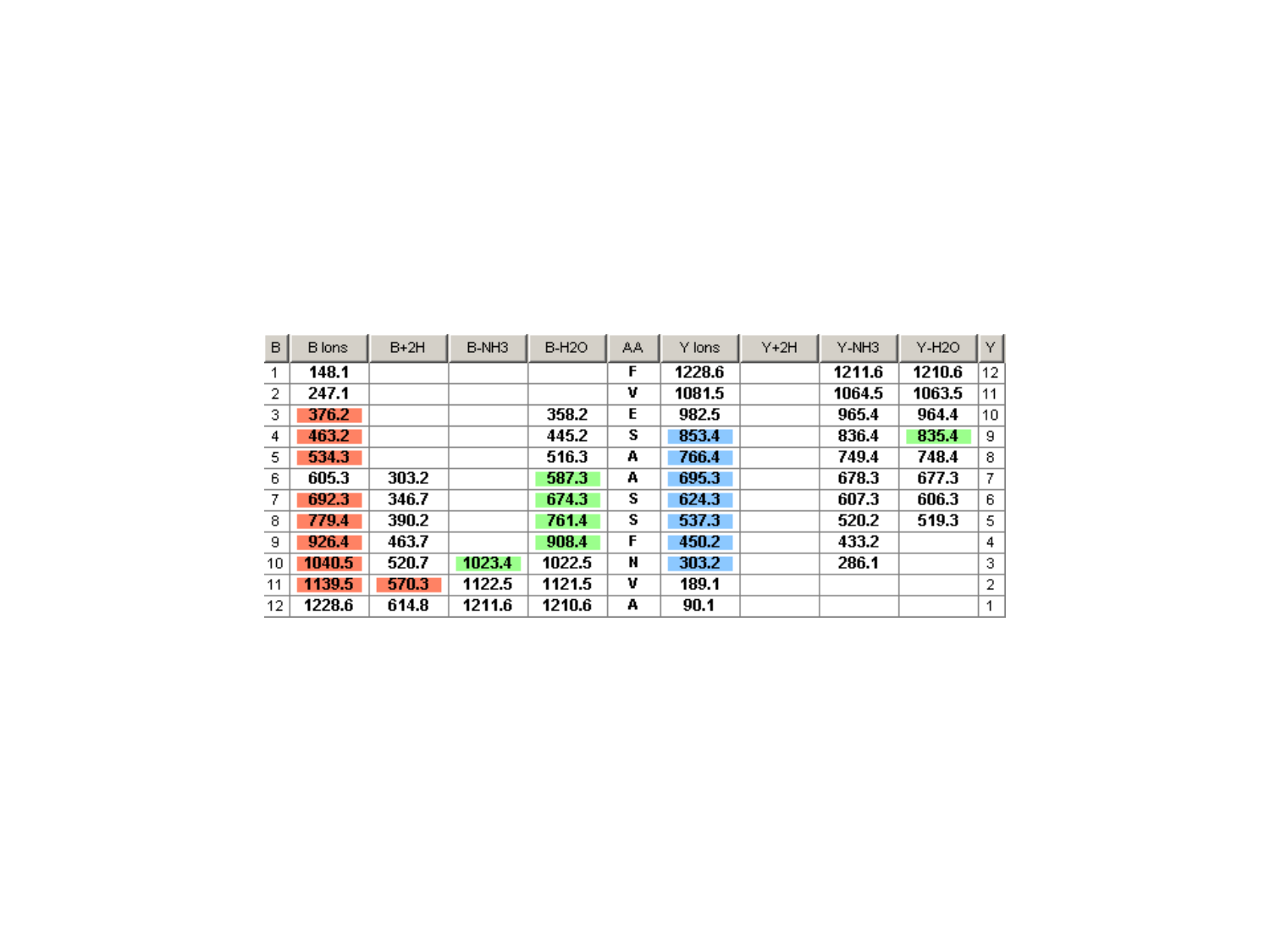

## Slide 15
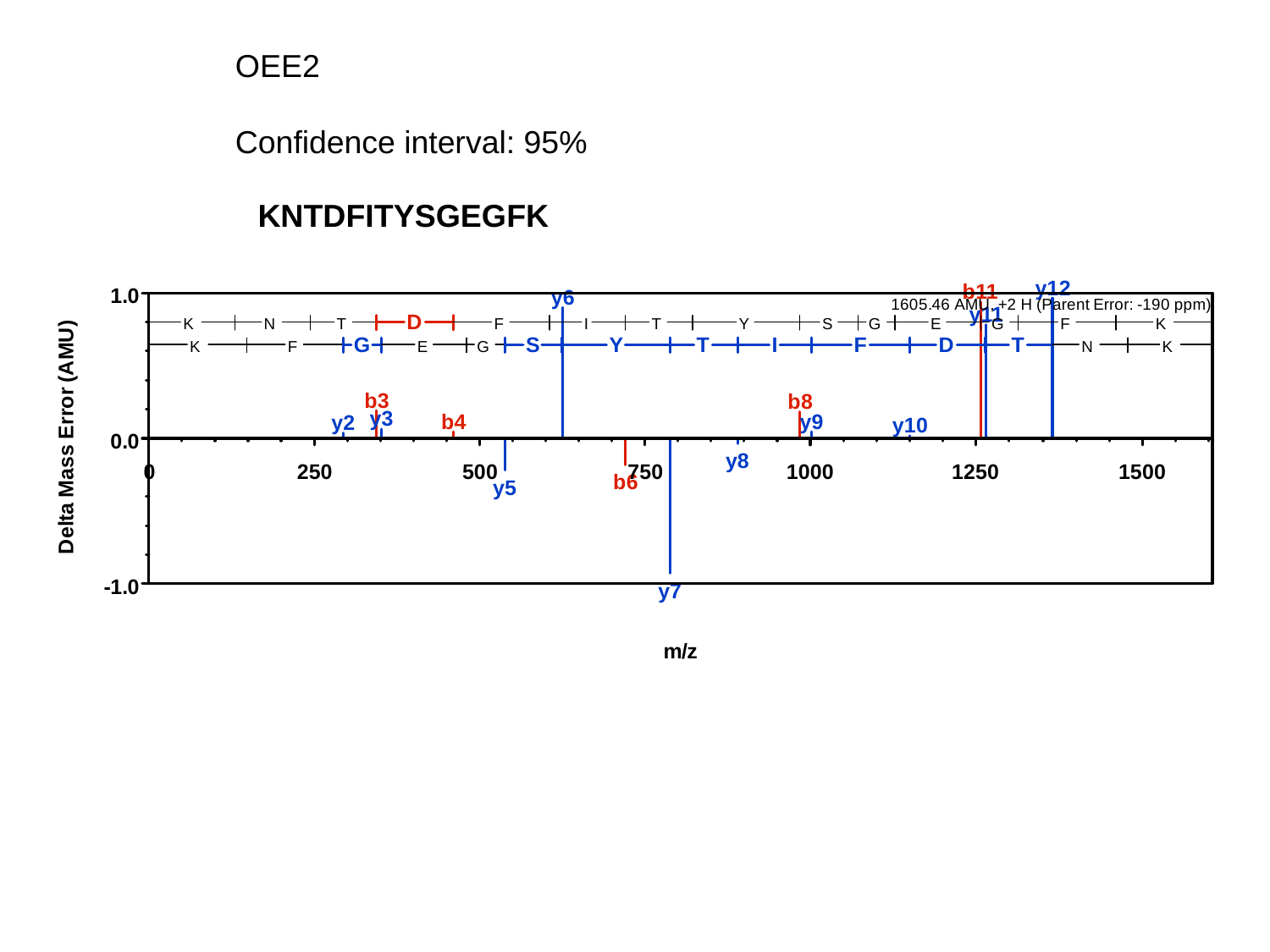

OEE2
Confidence interval: 95%
KNTDFITYSGEGFK

## Slide 16
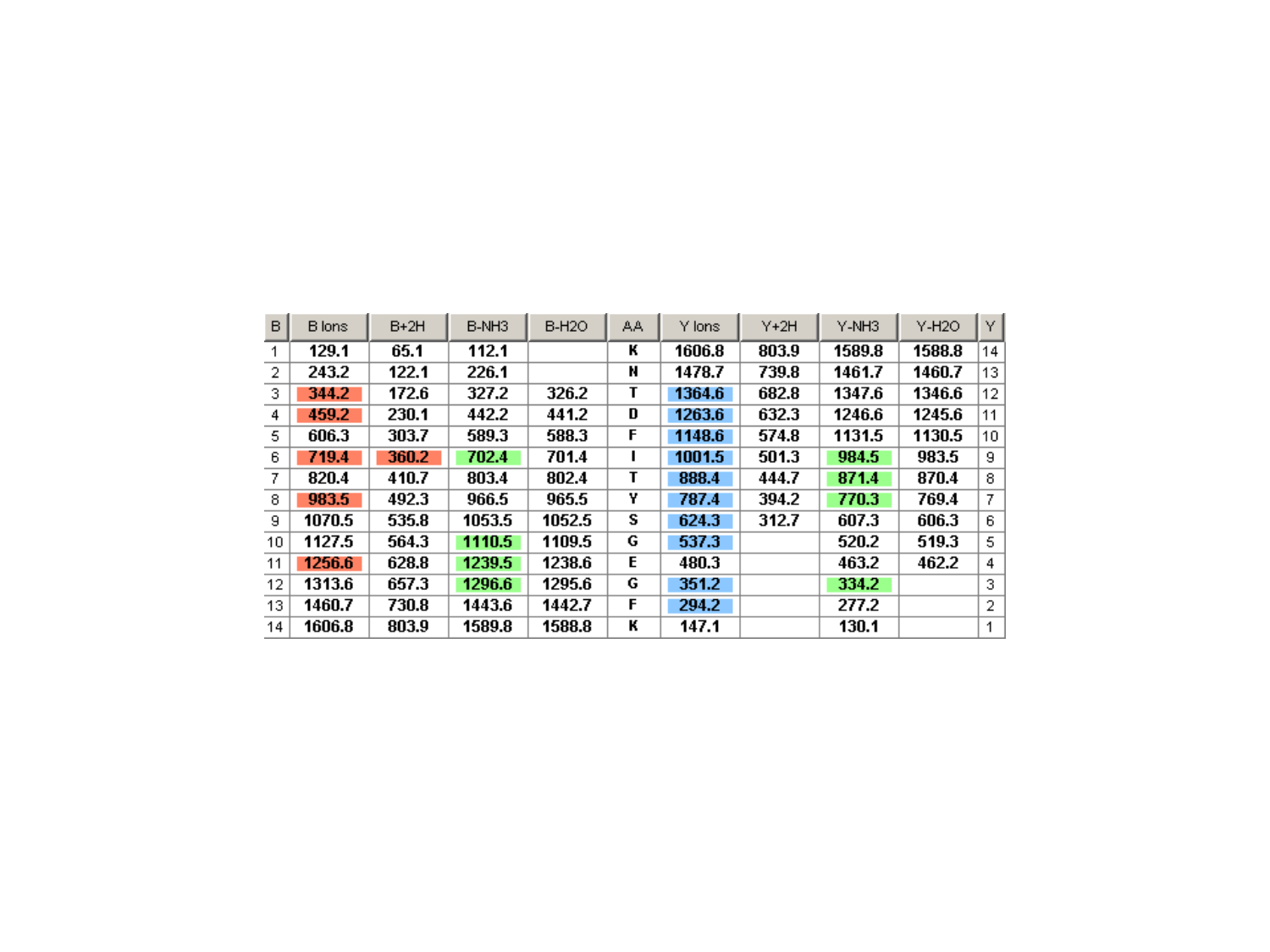

## Slide 17
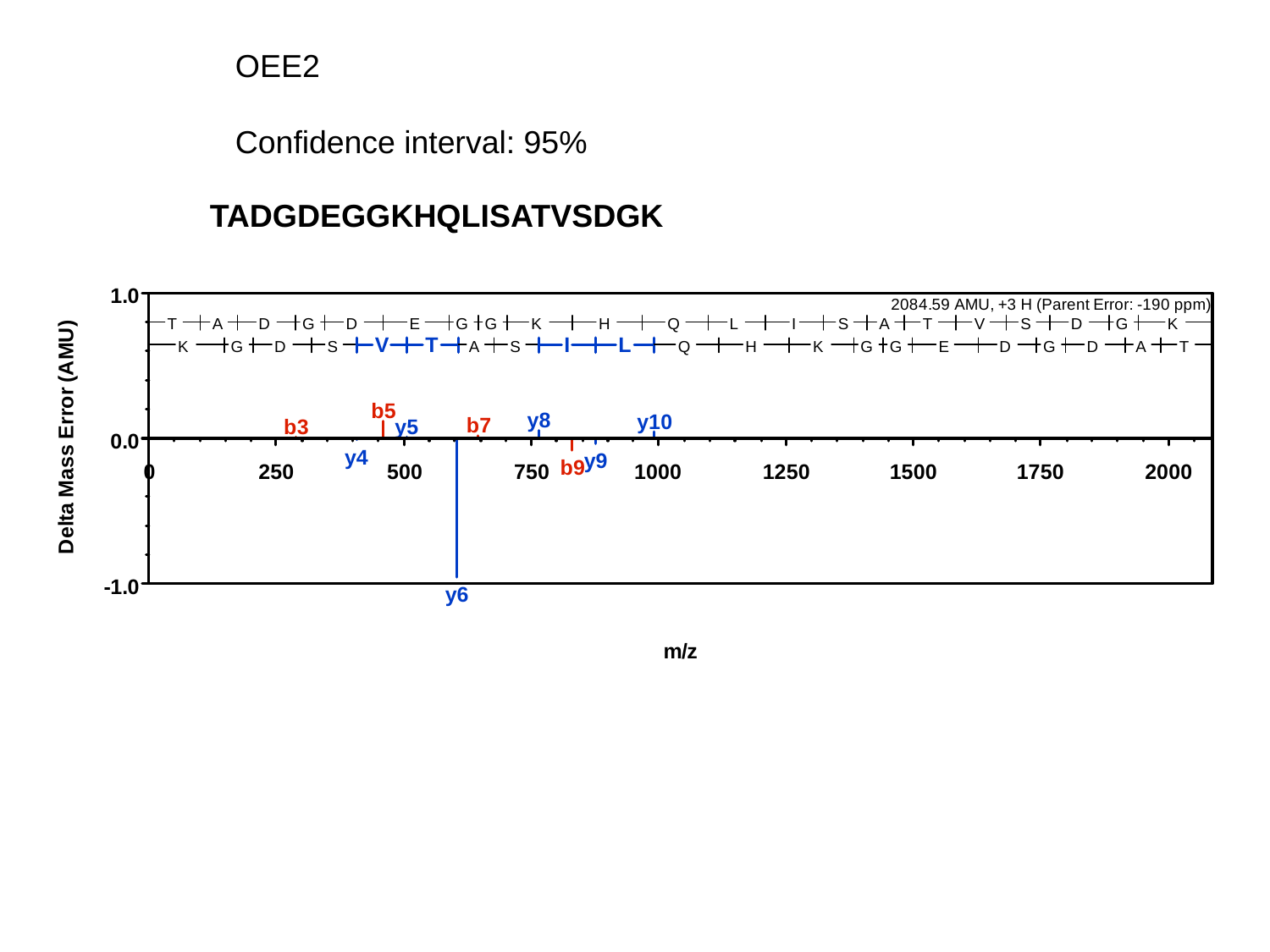

OEE2
Confidence interval: 95%
TADGDEGGKHQLISATVSDGK

## Slide 18
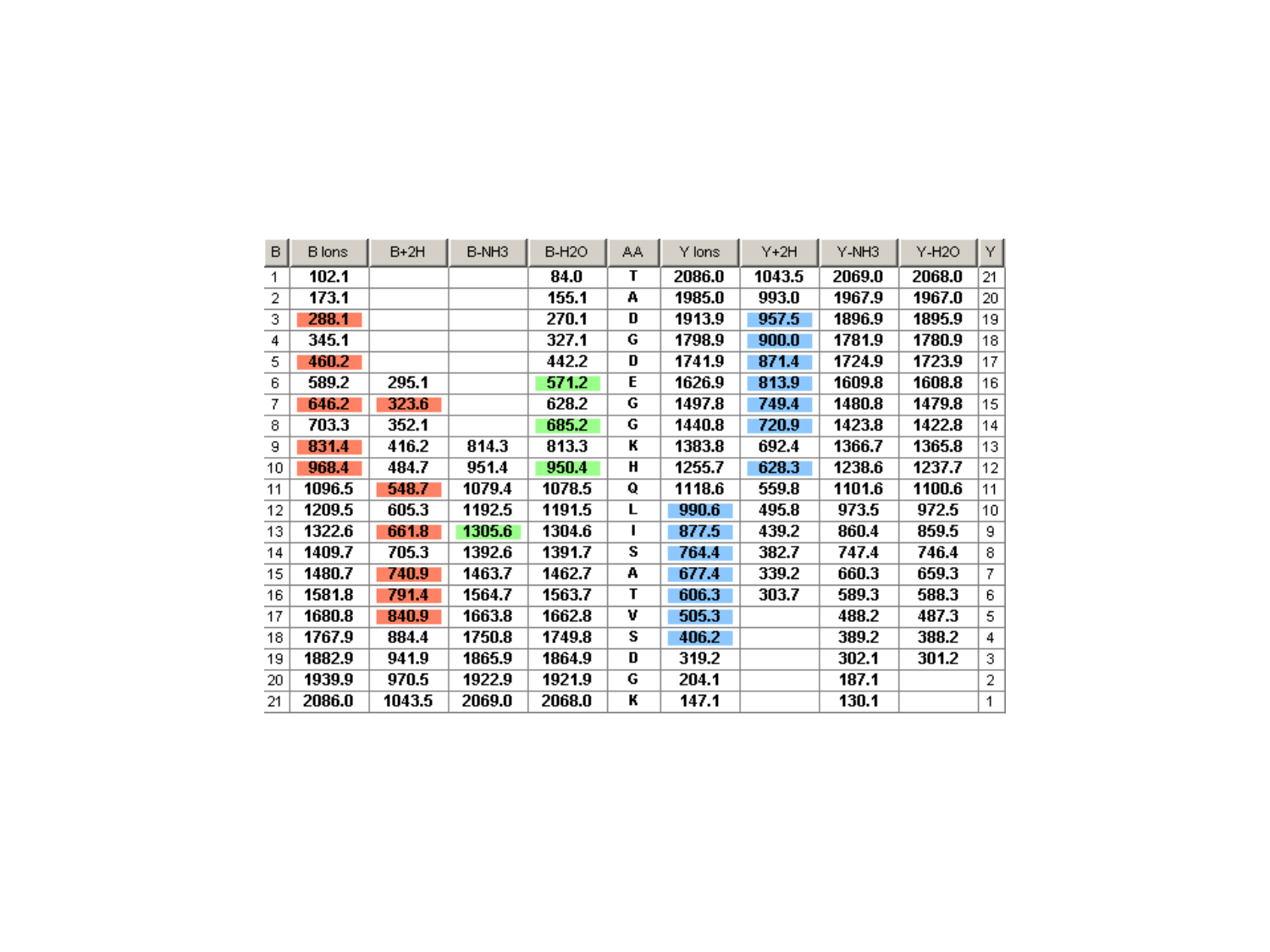

## Slide 19
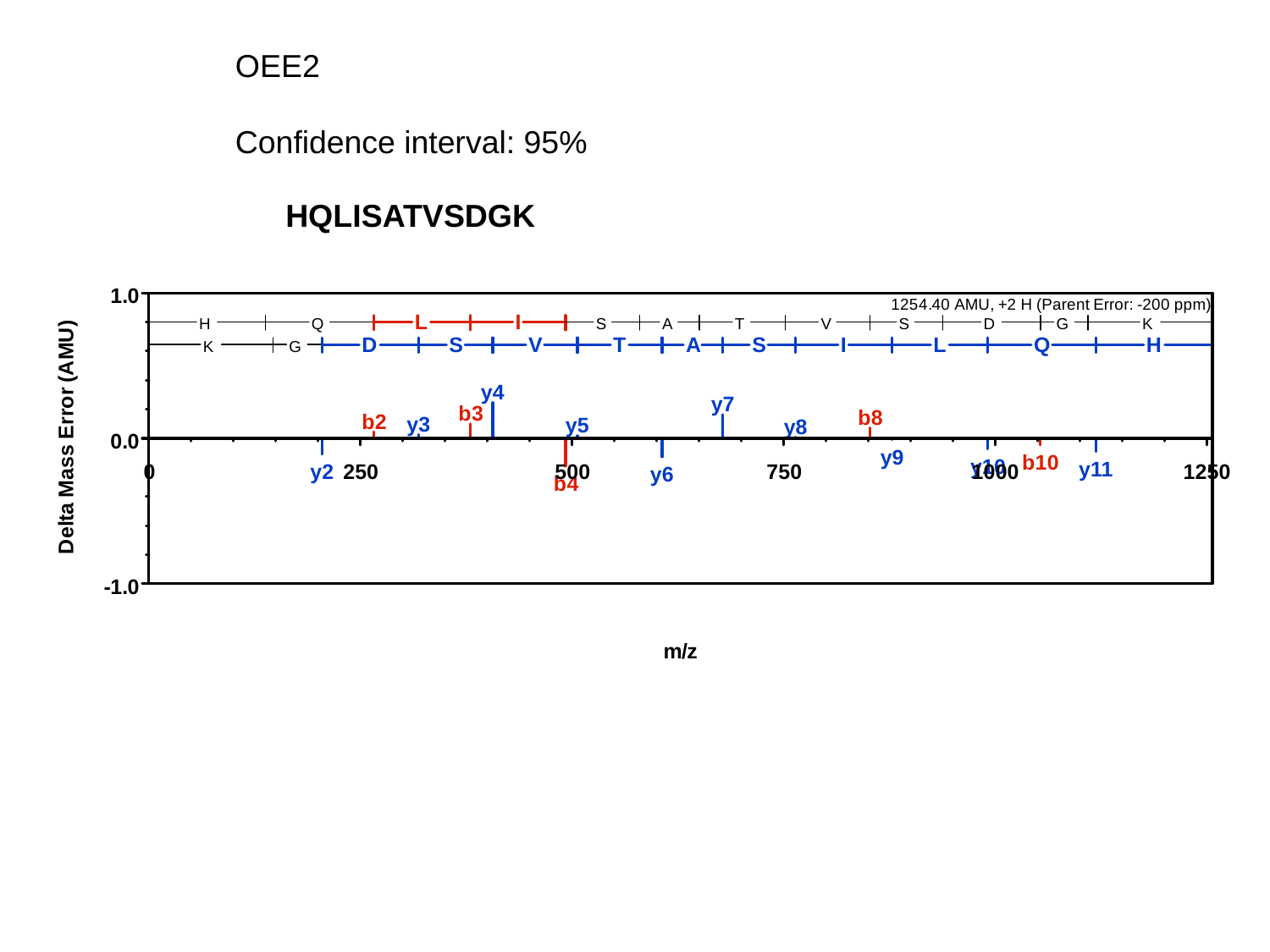

OEE2
Confidence interval: 95%
HQLISATVSDGK

## Slide 20
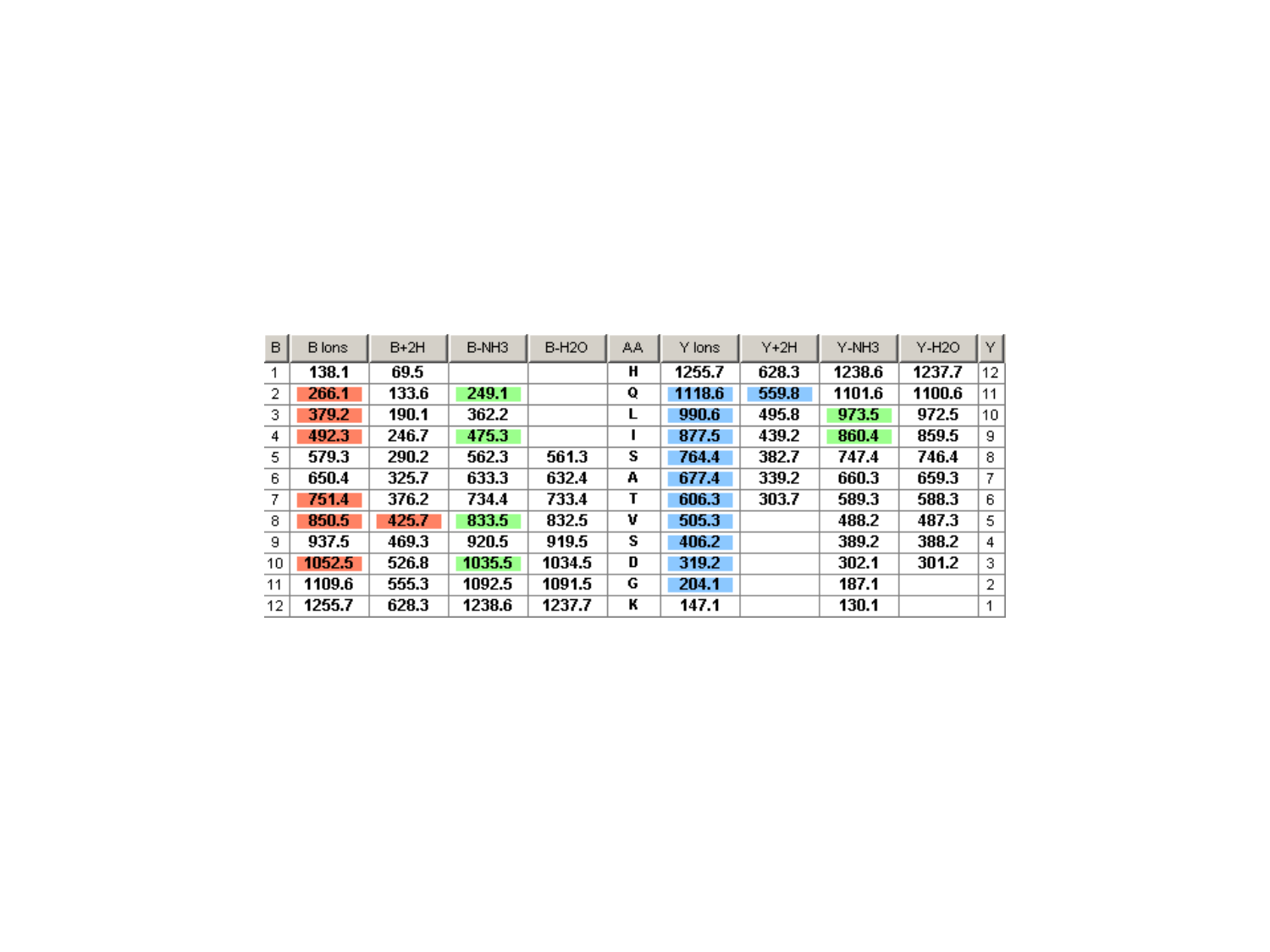

## Slide 21
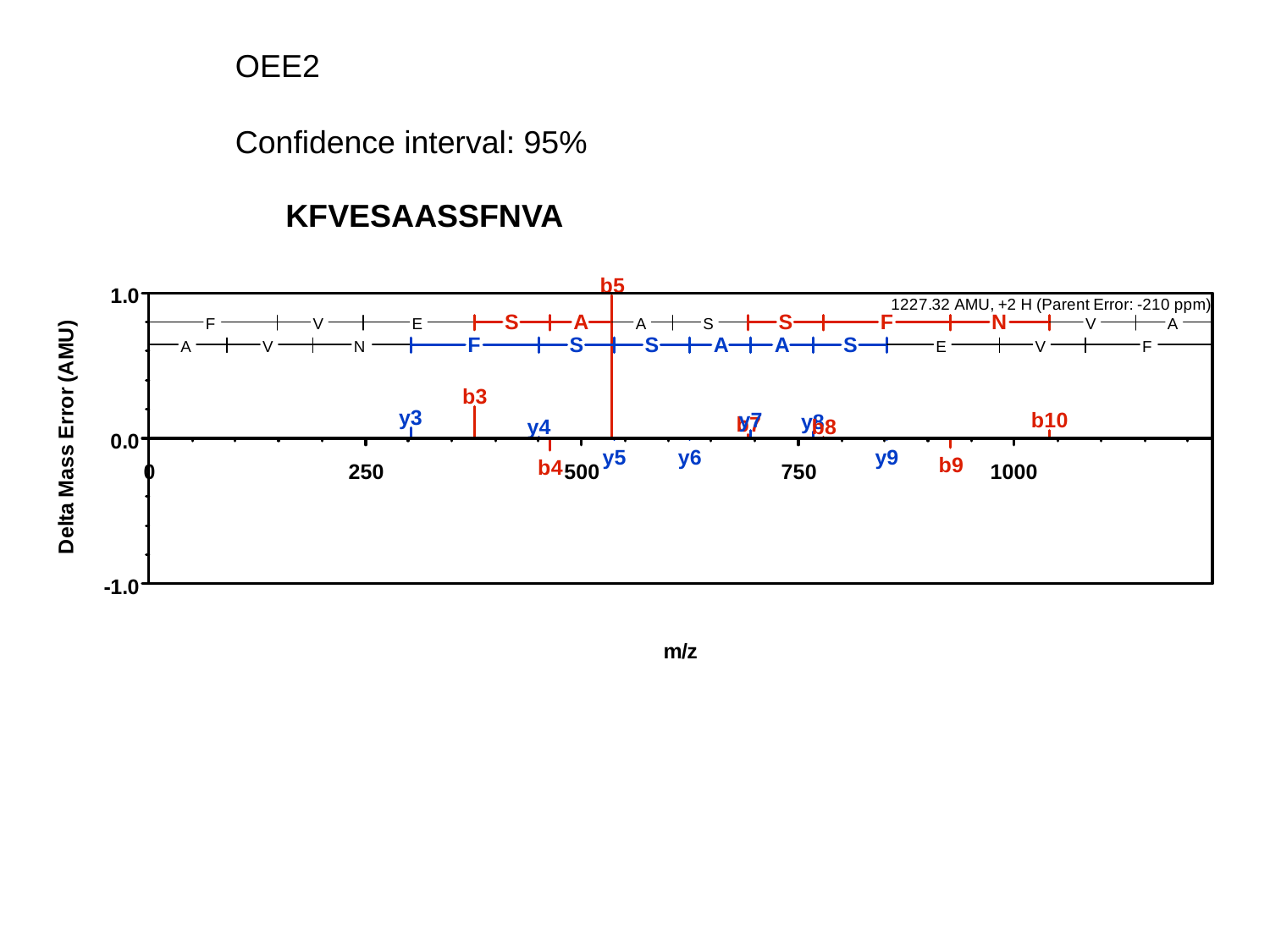

OEE2
Confidence interval: 95%
KFVESAASSFNVA

## Slide 22
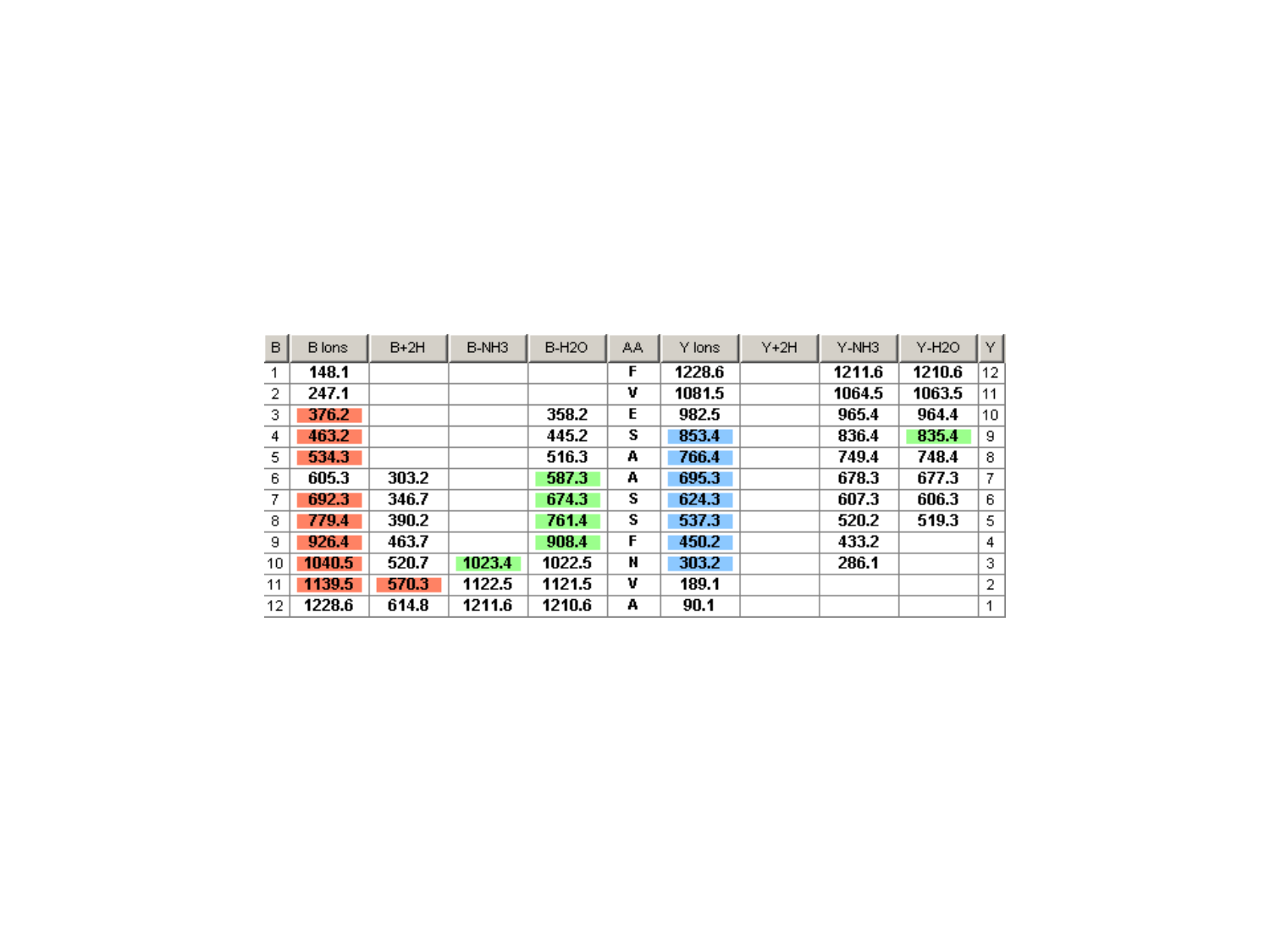

## Slide 23
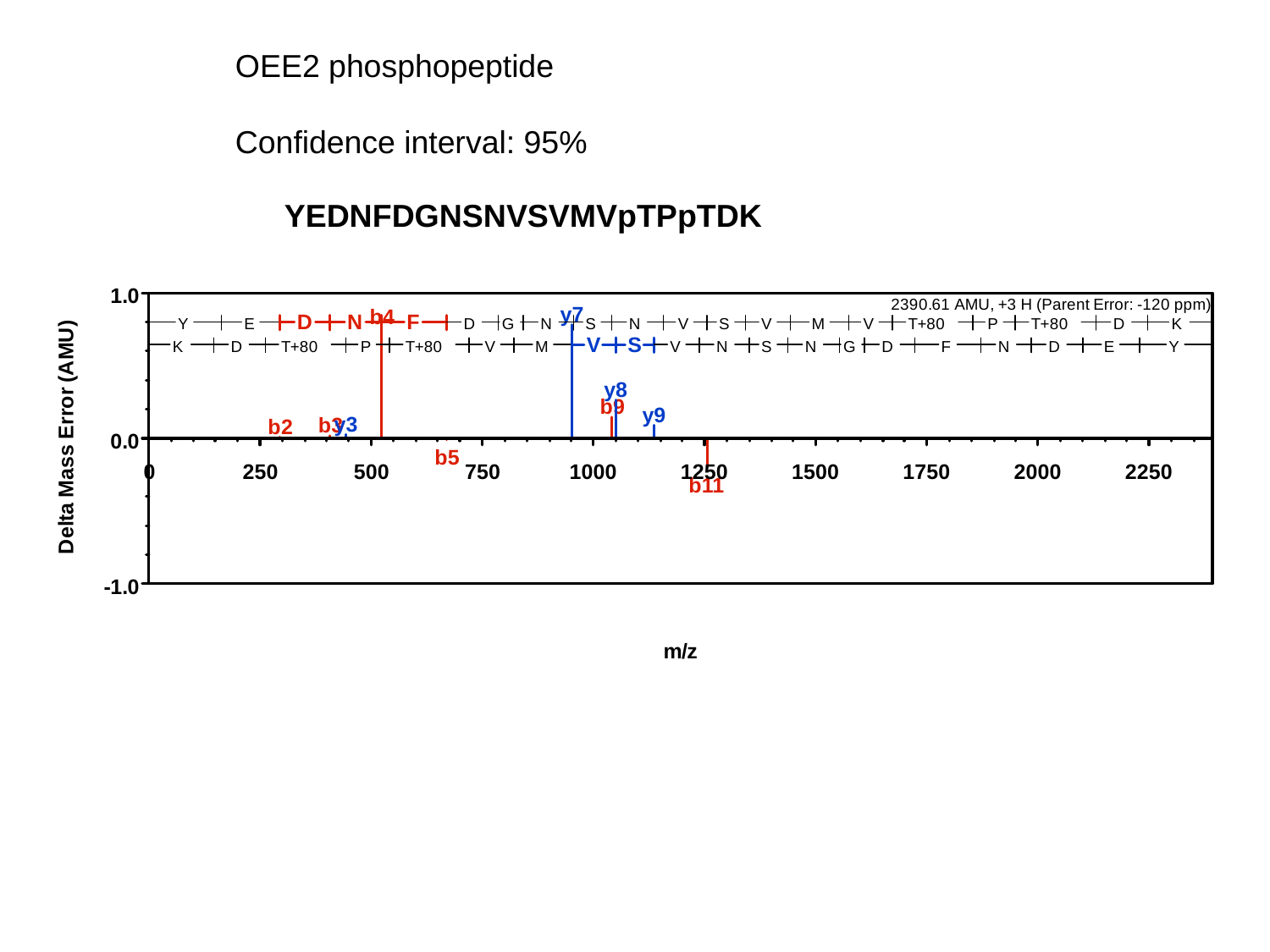

OEE2 phosphopeptide
Confidence interval: 95%
YEDNFDGNSNVSVMVpTPpTDK

## Slide 24
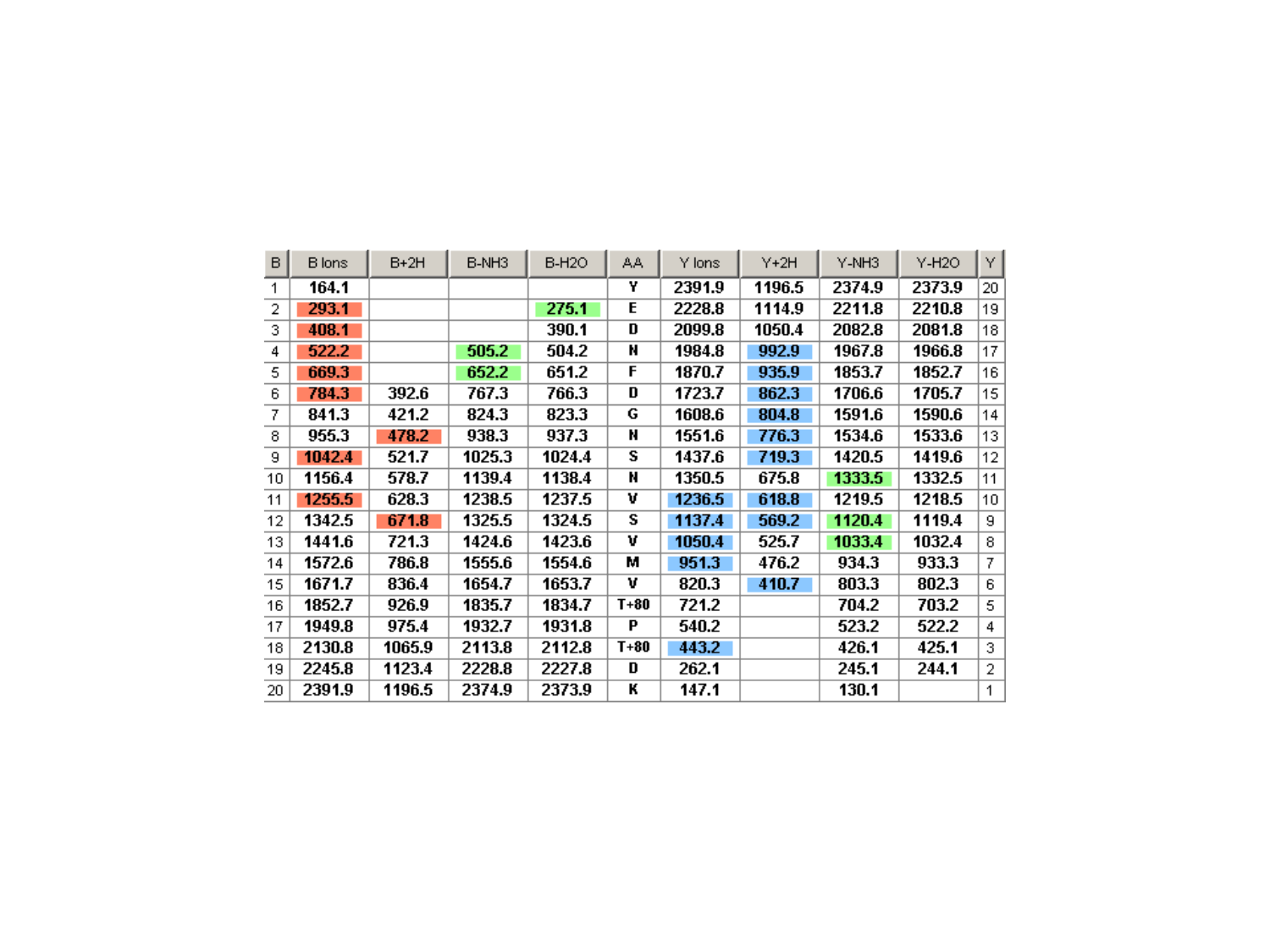

## Slide 25
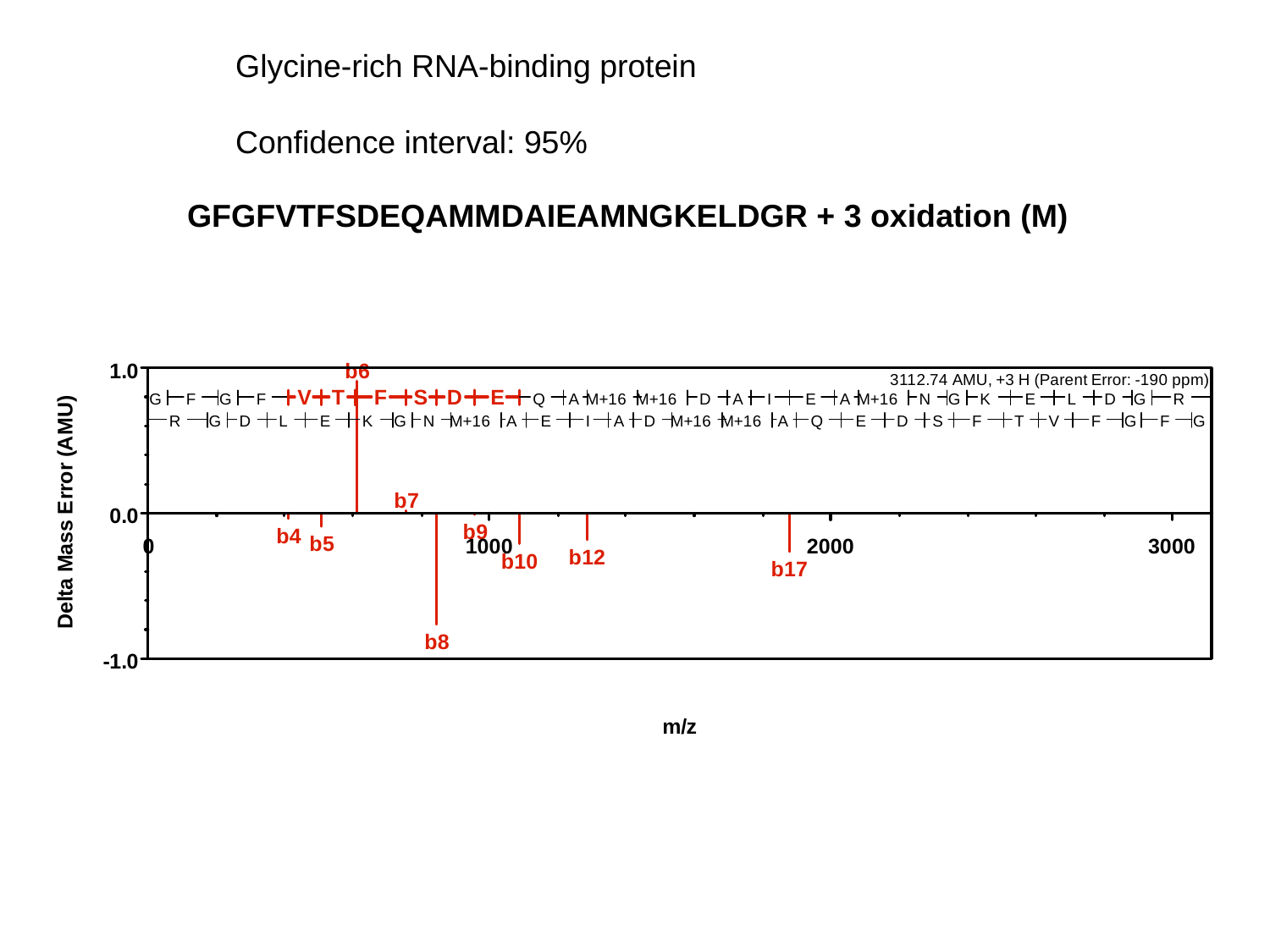

Glycine-rich RNA-binding protein
Confidence interval: 95%
GFGFVTFSDEQAMMDAIEAMNGKELDGR + 3 oxidation (M)

## Slide 26
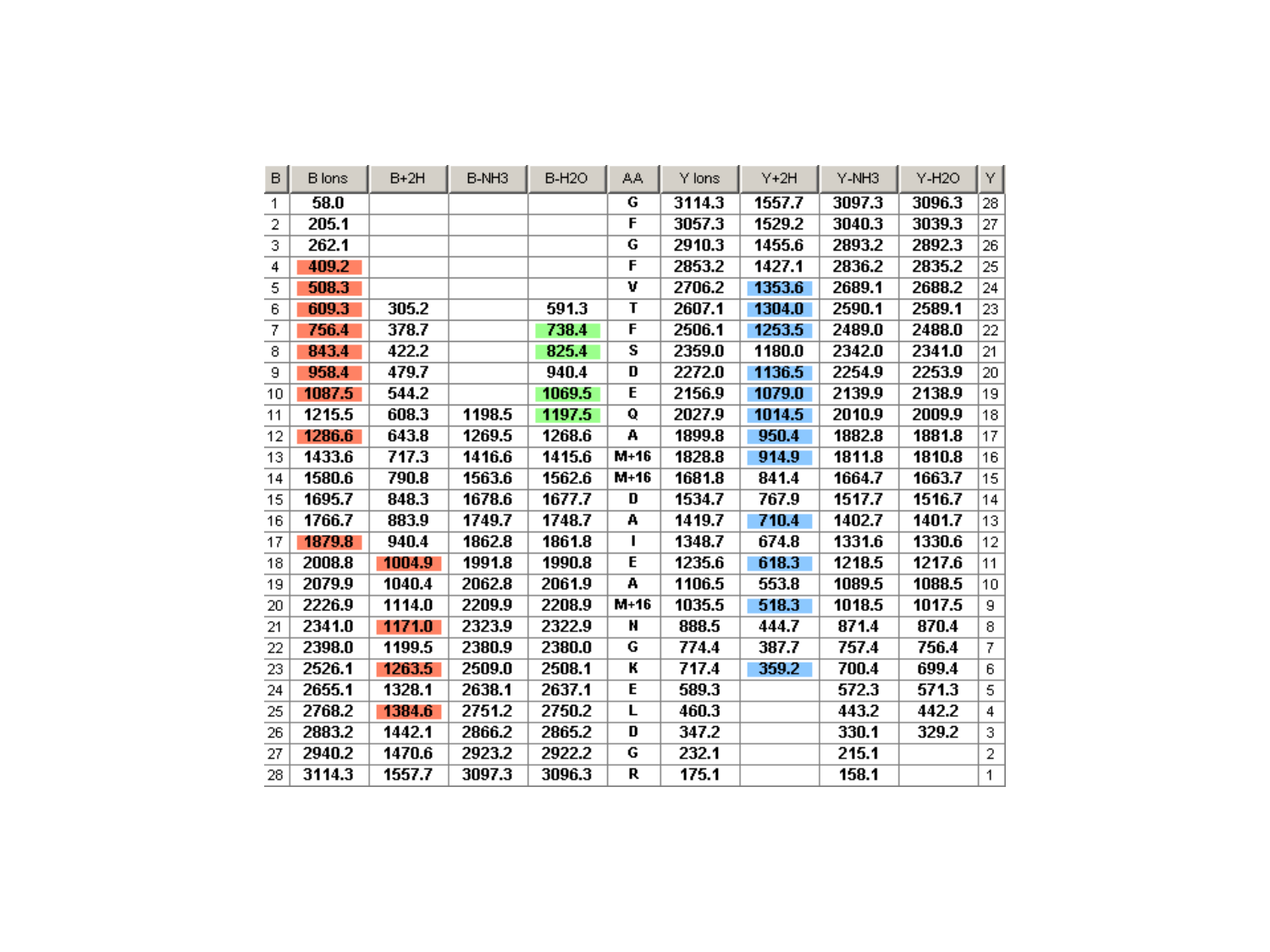

## Slide 27
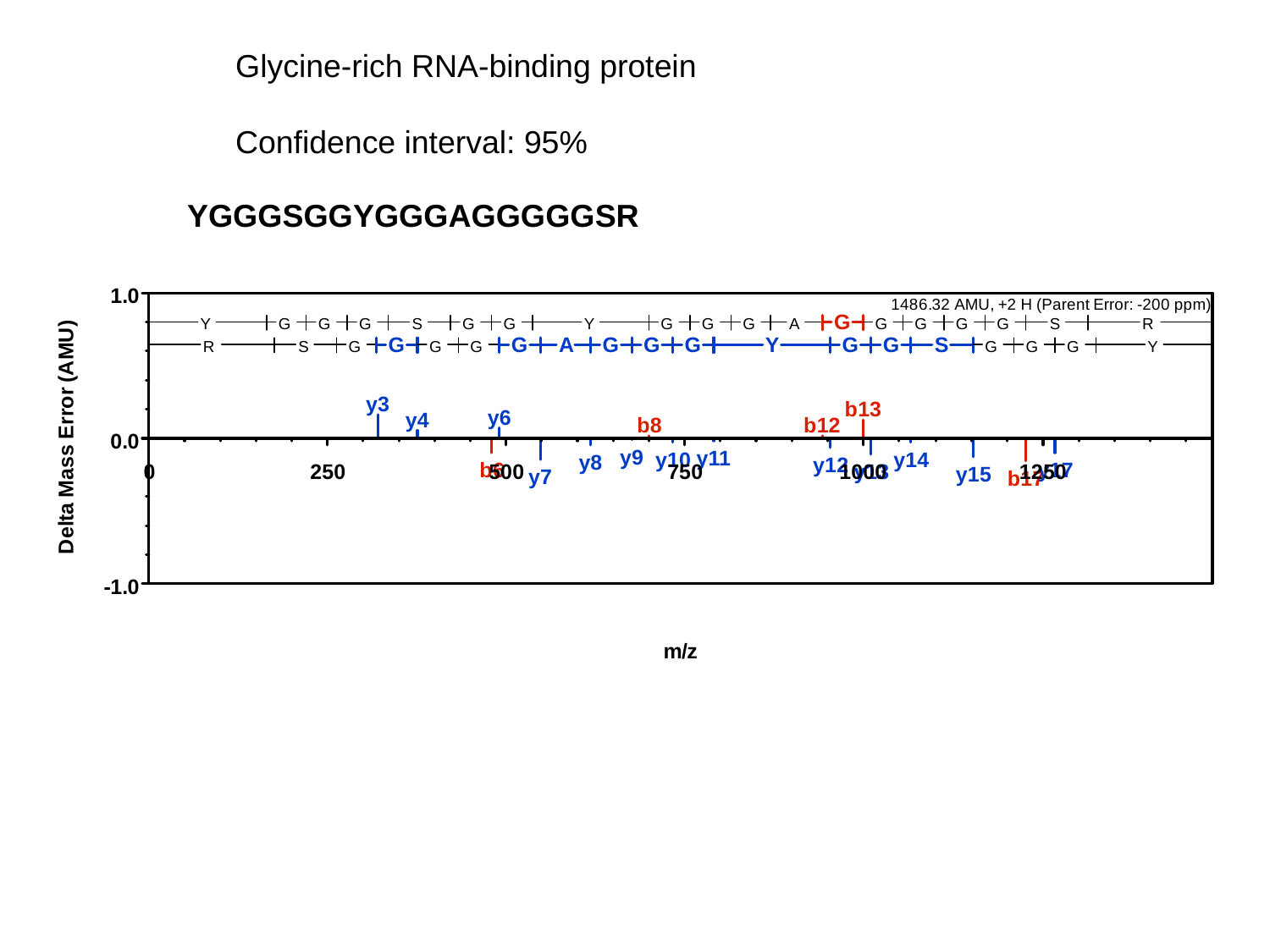

Glycine-rich RNA-binding protein
Confidence interval: 95%
YGGGSGGYGGGAGGGGGSR

## Slide 28
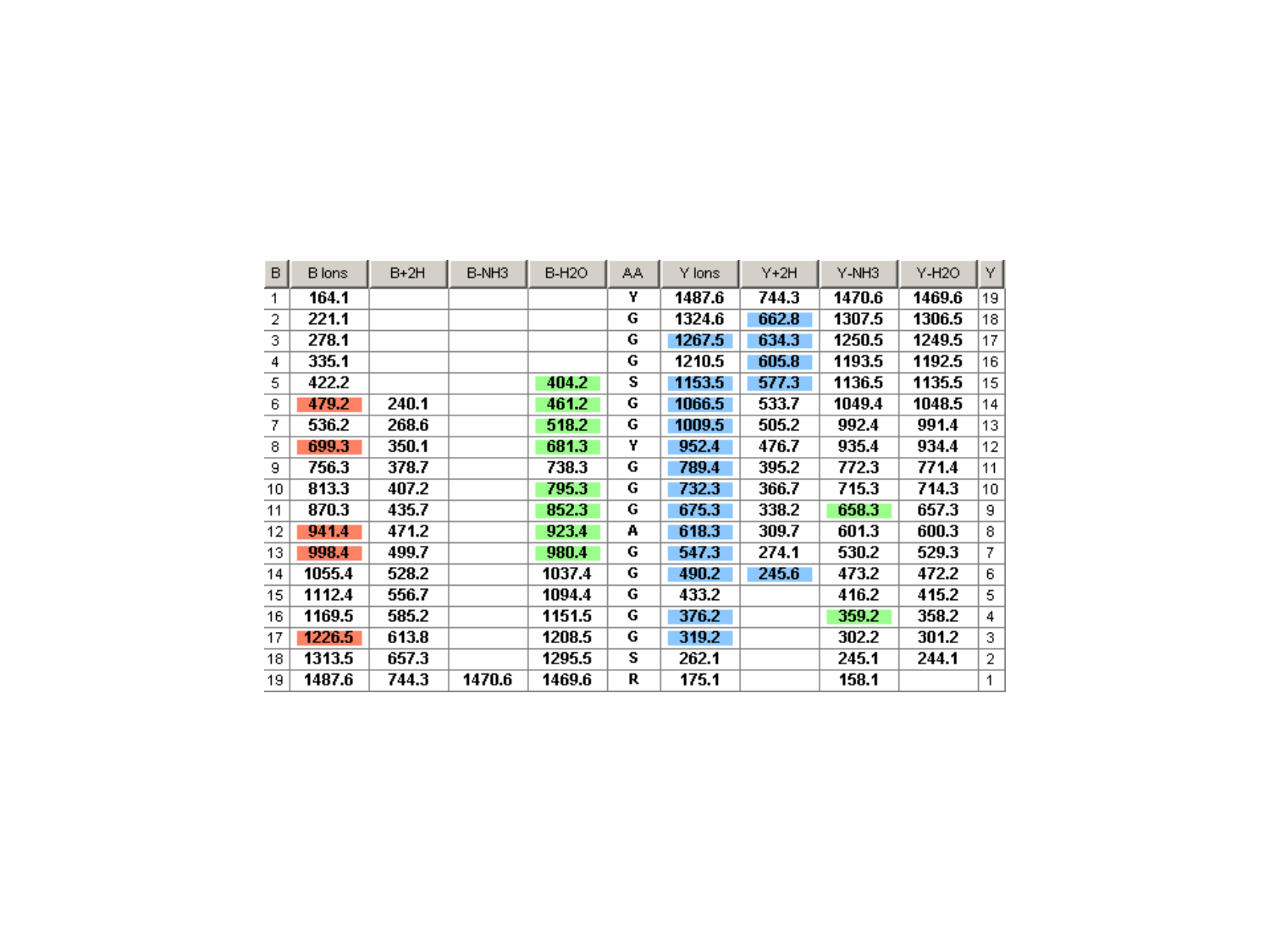

## Slide 29
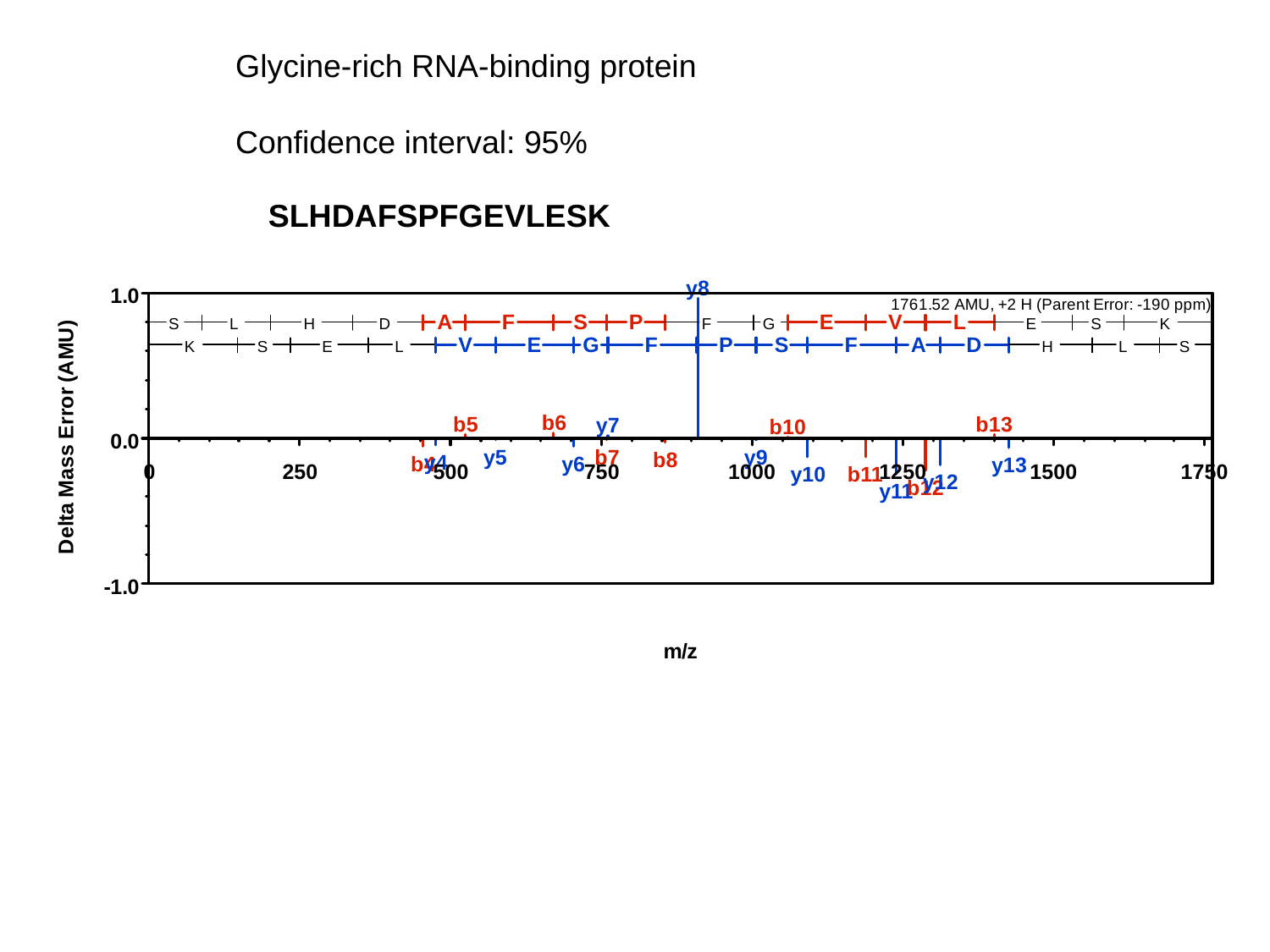

Glycine-rich RNA-binding protein
Confidence interval: 95%
SLHDAFSPFGEVLESK

## Slide 30
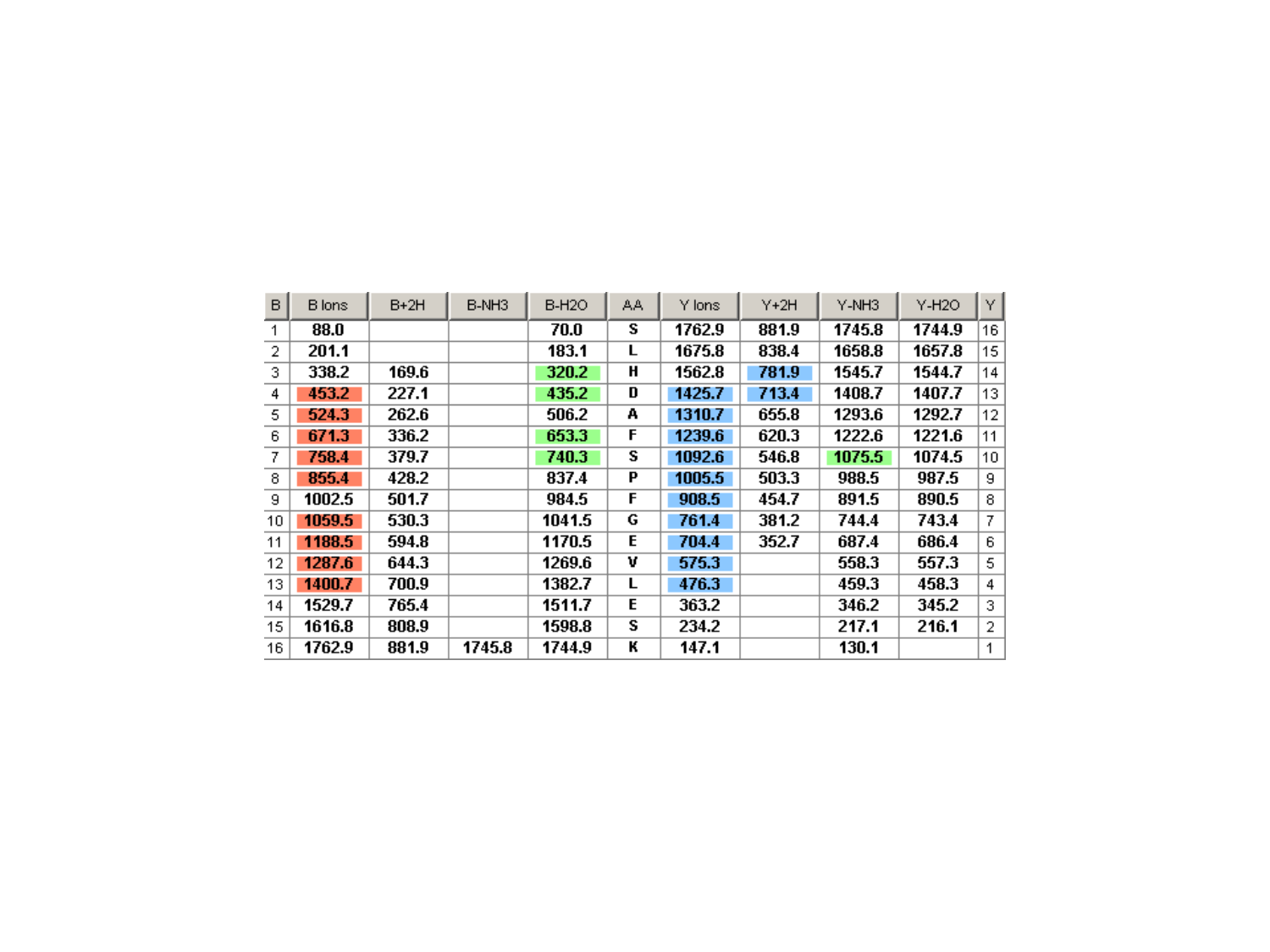

## Slide 31
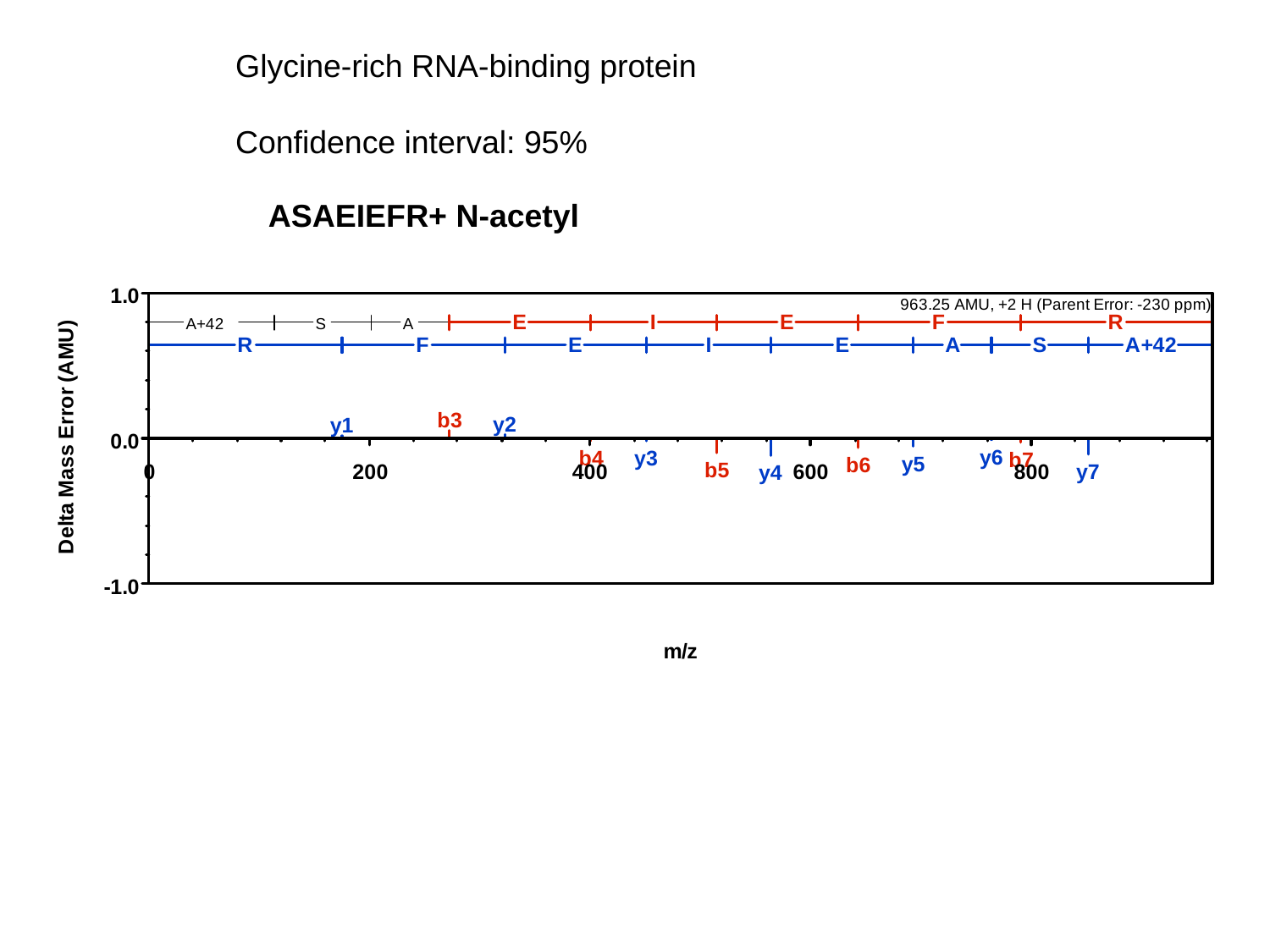

Glycine-rich RNA-binding protein
Confidence interval: 95%
ASAEIEFR+ N-acetyl

## Slide 32
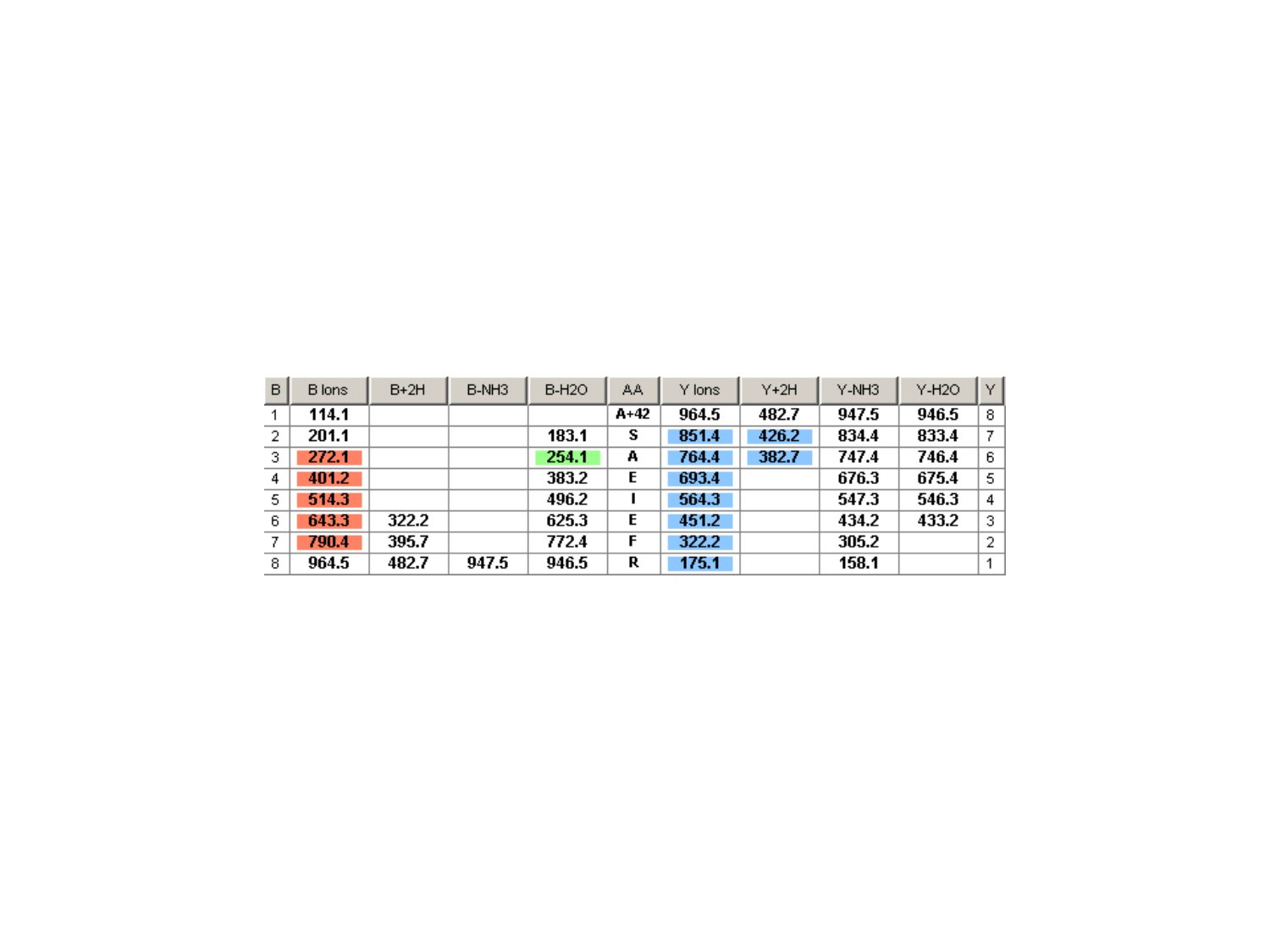

## Slide 33
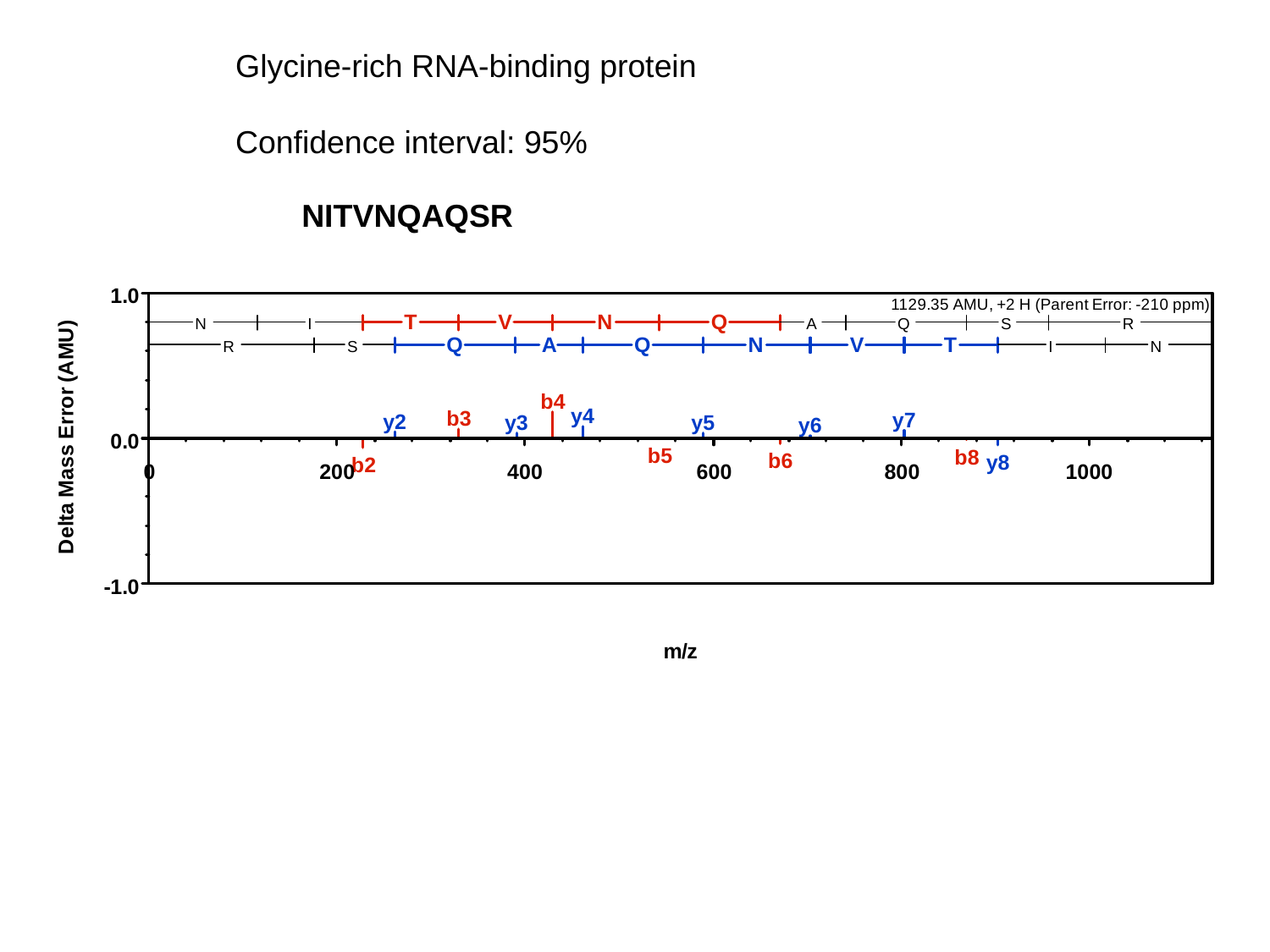

Glycine-rich RNA-binding protein
Confidence interval: 95%
NITVNQAQSR

## Slide 34
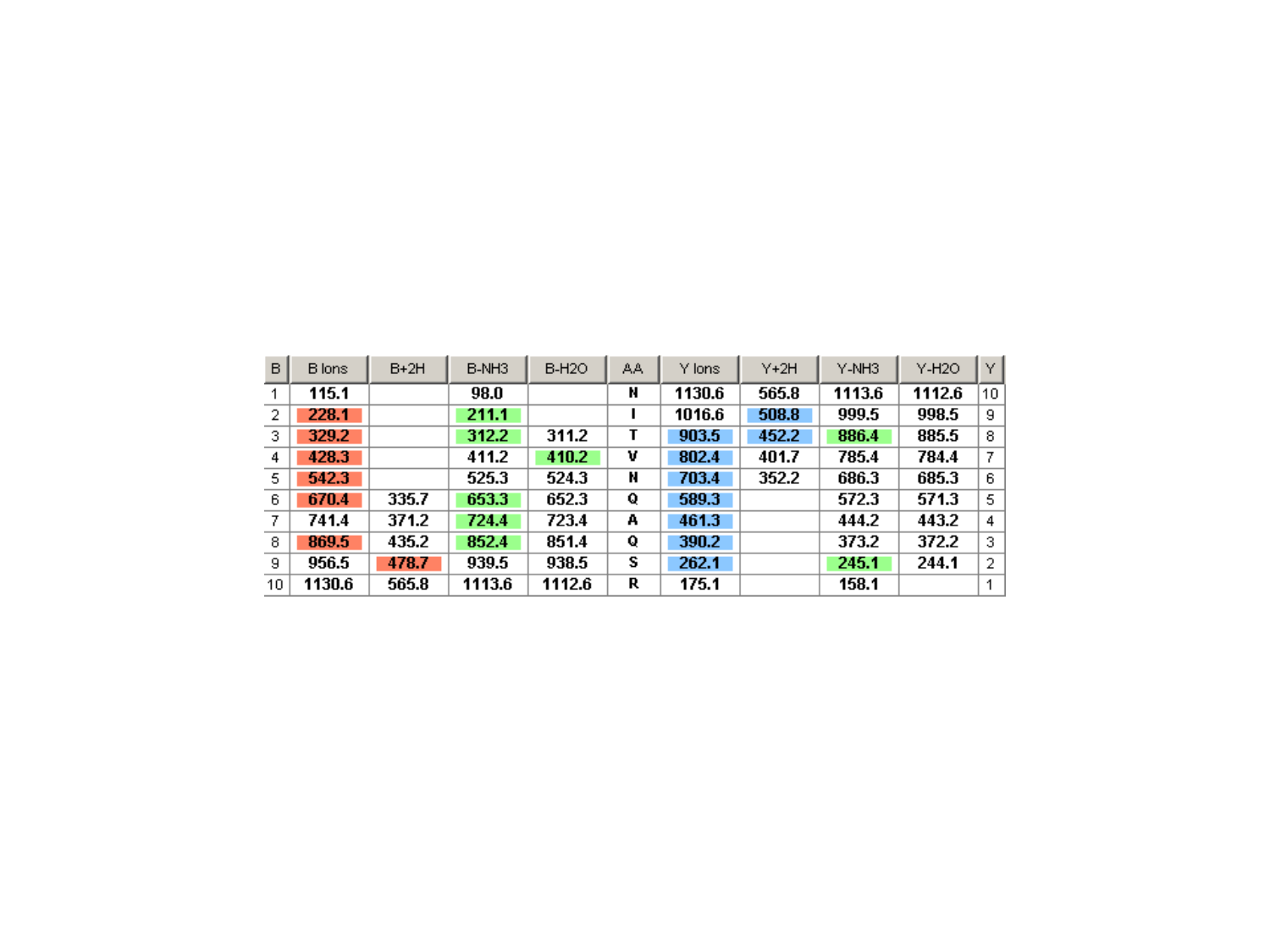

## Slide 35
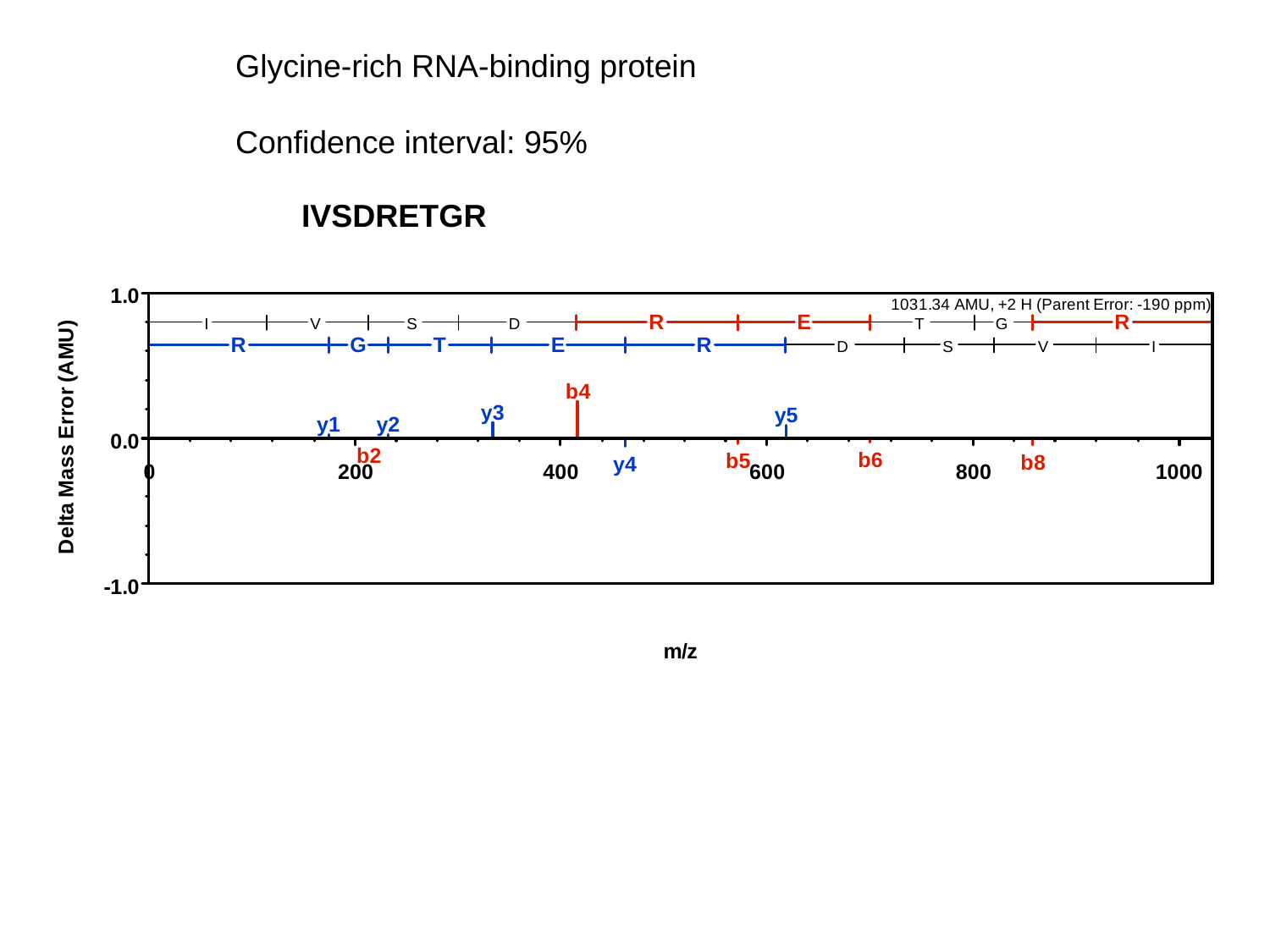

Glycine-rich RNA-binding protein
Confidence interval: 95%
IVSDRETGR

## Slide 36
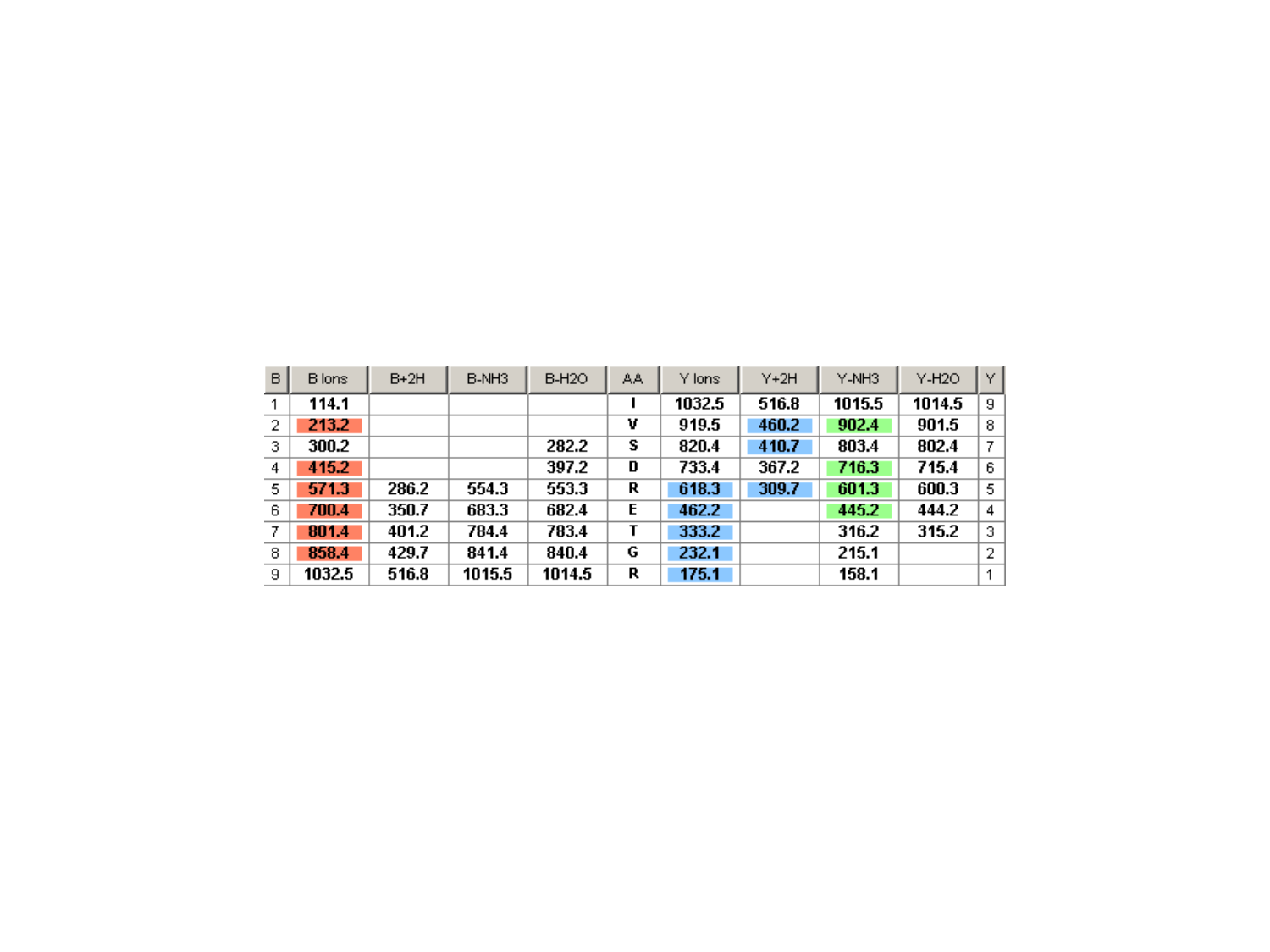

## Slide 37
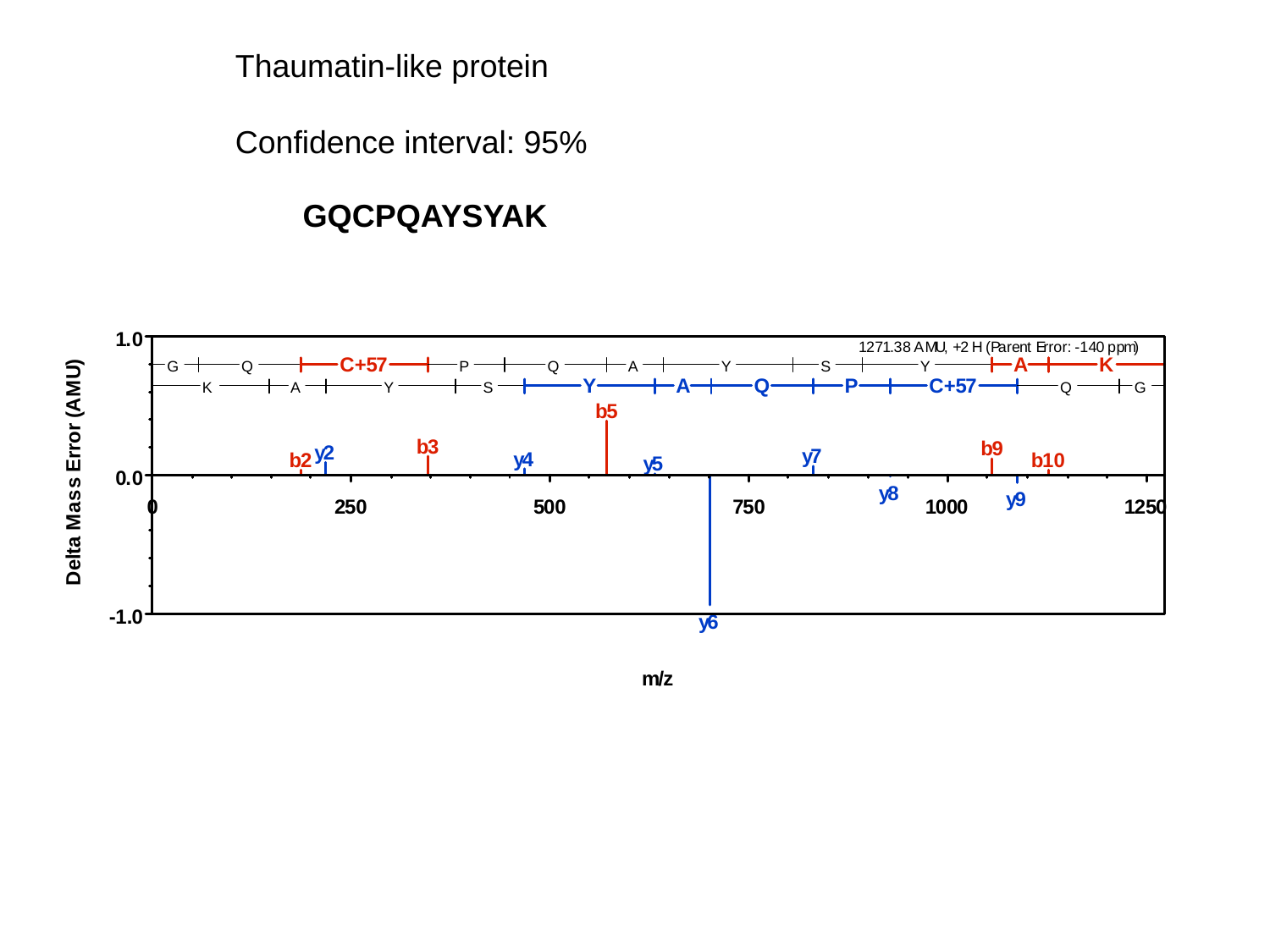

Thaumatin-like protein
Confidence interval: 95%
GQCPQAYSYAK

## Slide 38
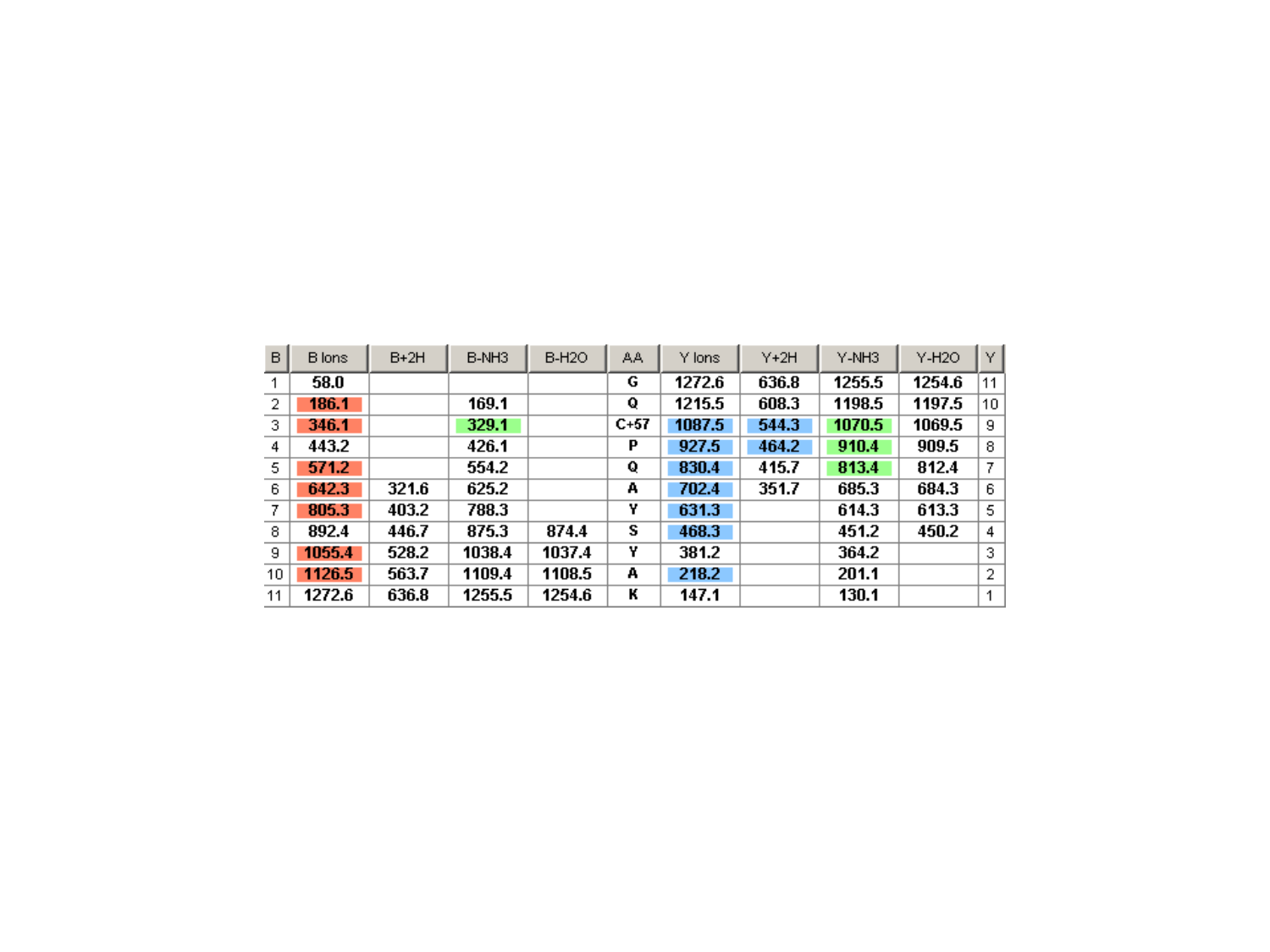

## Slide 39
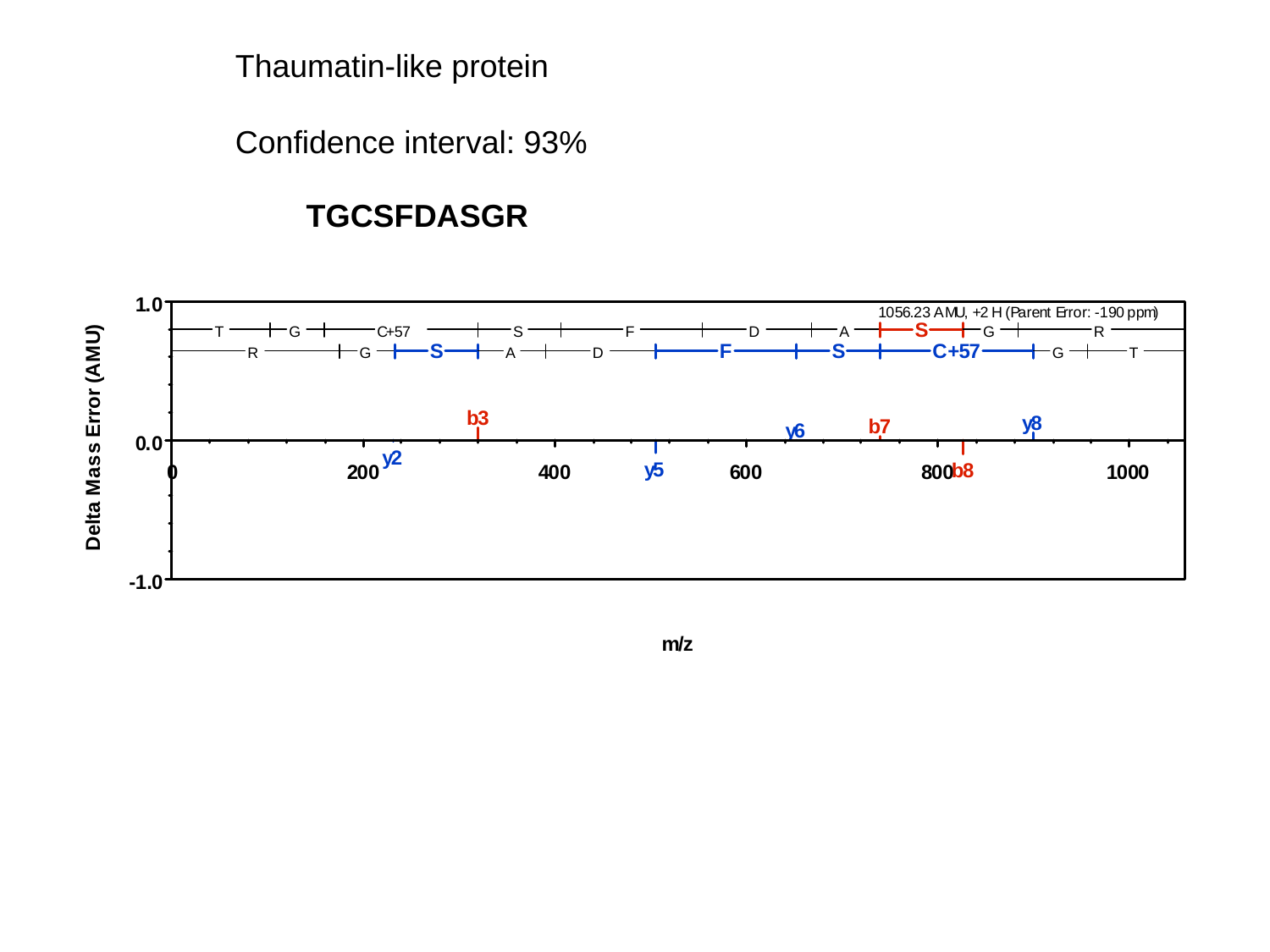

Thaumatin-like protein
Confidence interval: 93%
TGCSFDASGR

## Slide 40
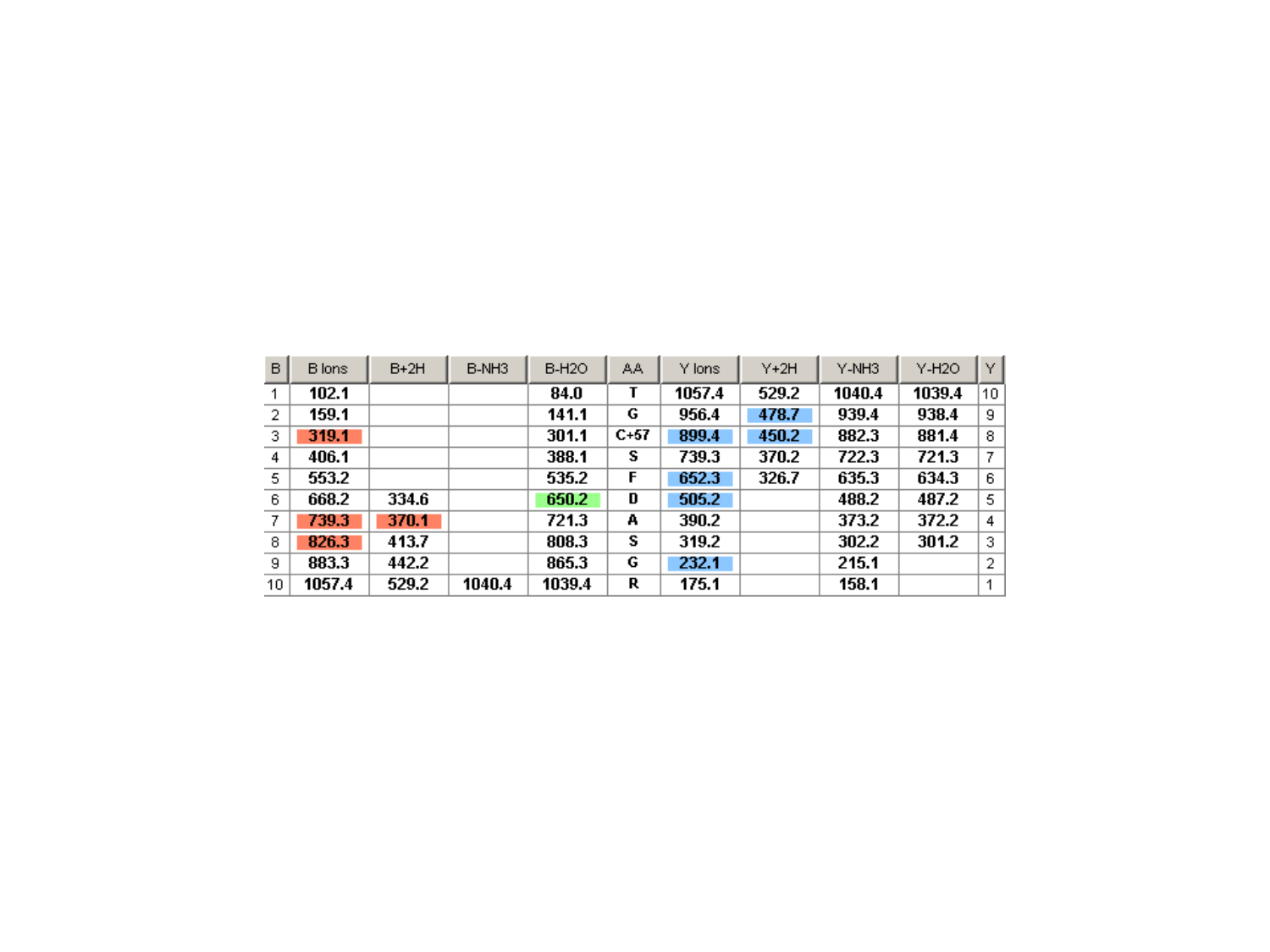

## Slide 41
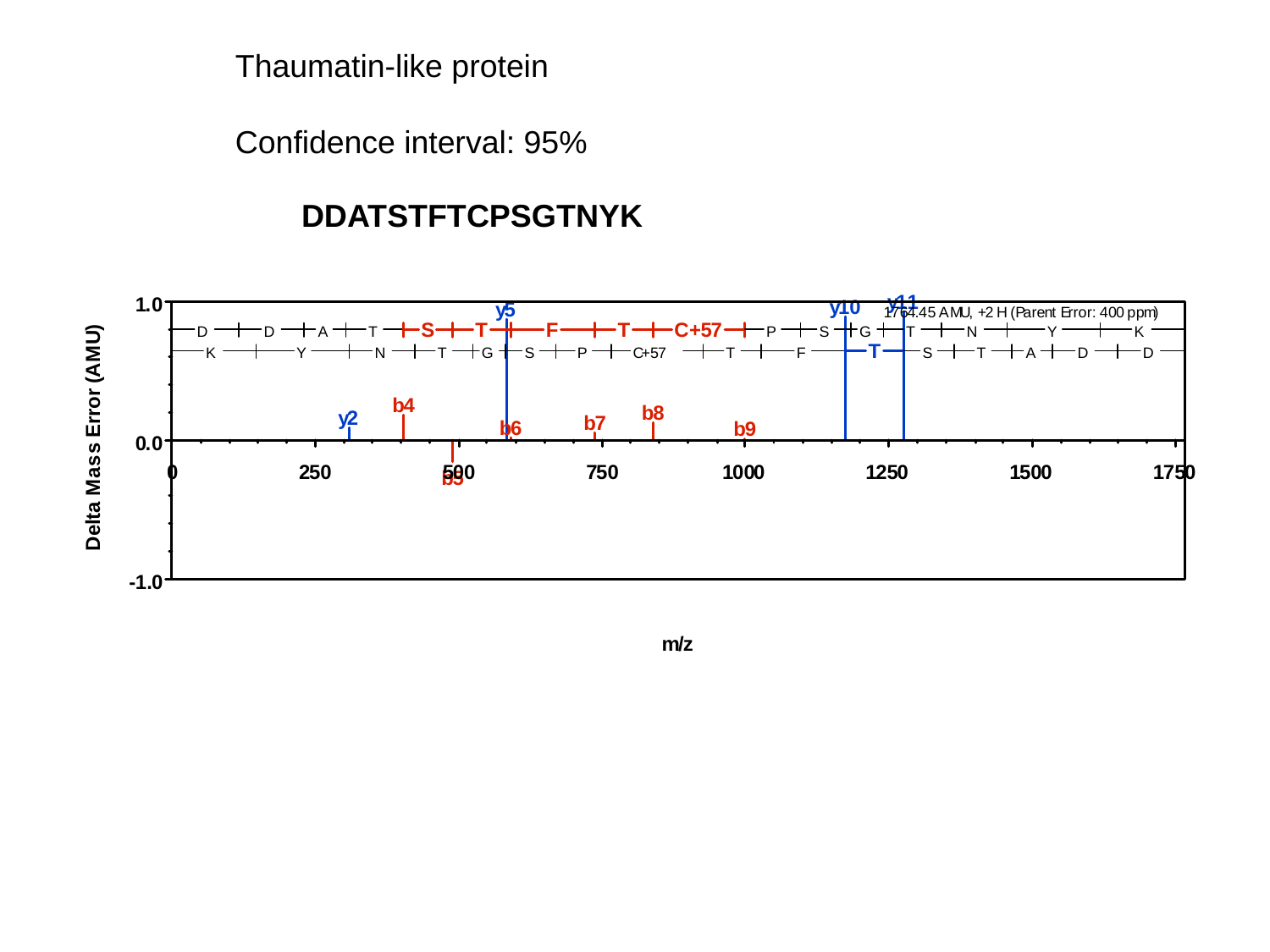

Thaumatin-like protein
Confidence interval: 95%
DDATSTFTCPSGTNYK

## Slide 42
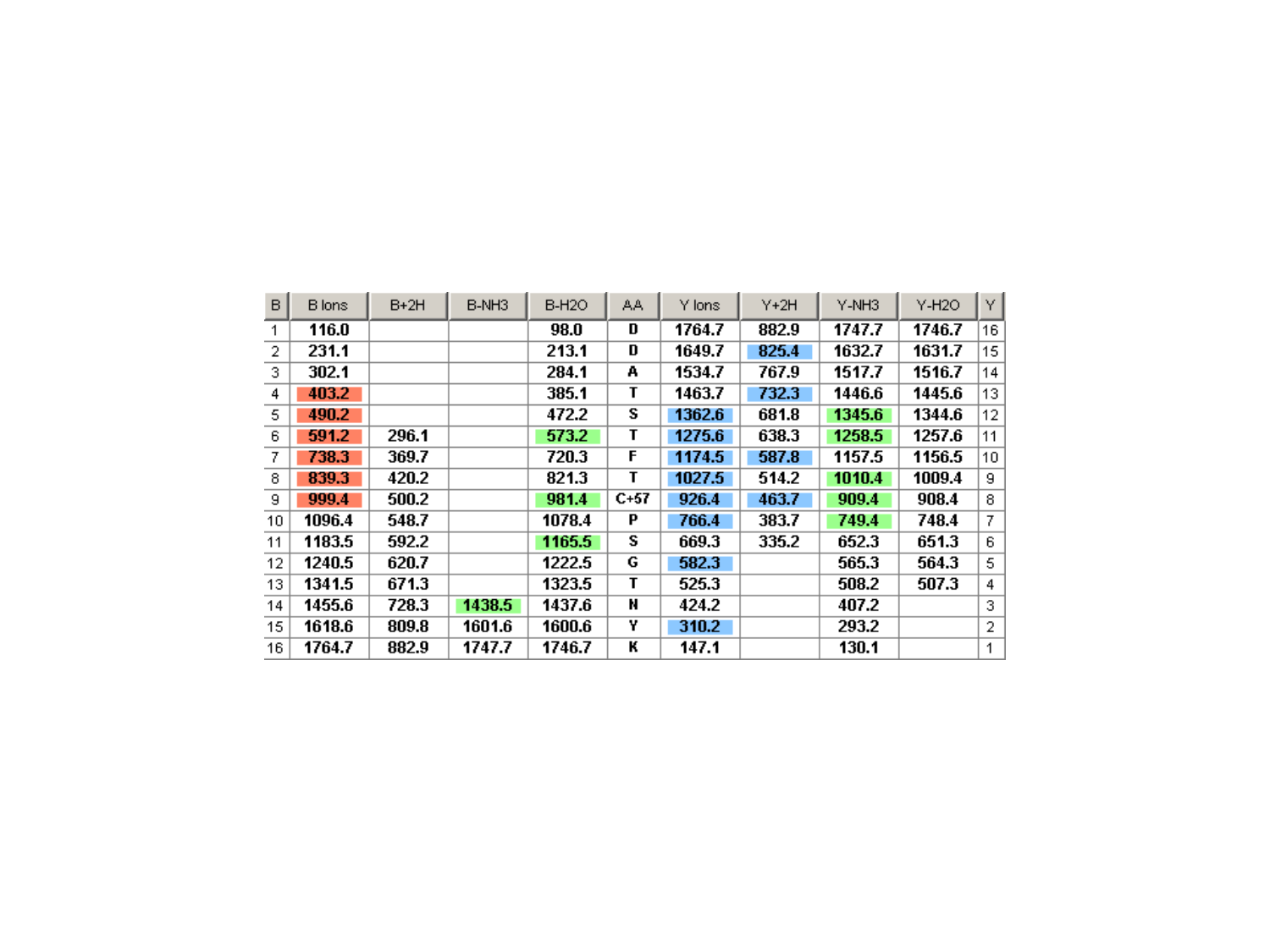

## Slide 43
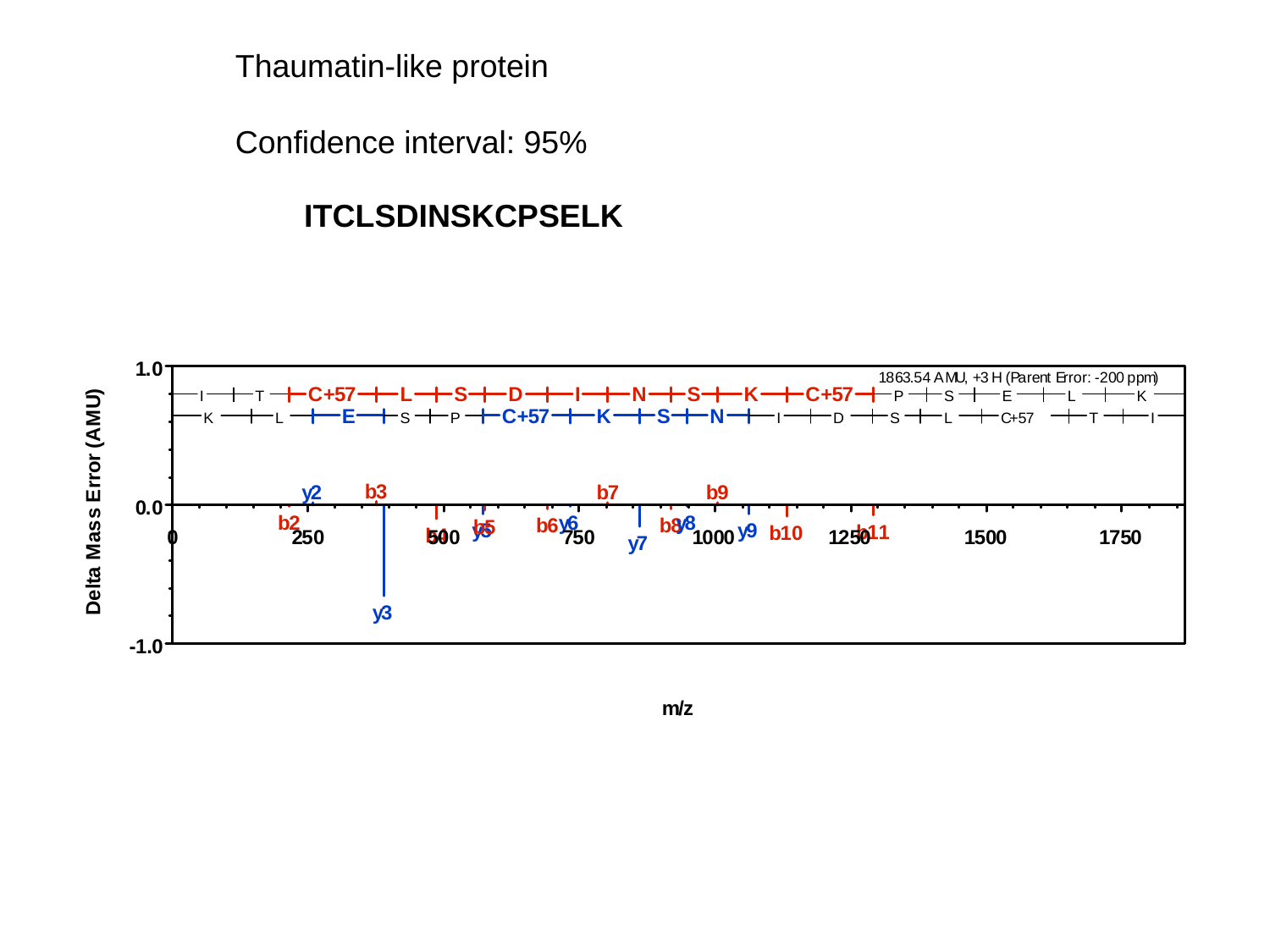

Thaumatin-like protein
Confidence interval: 95%
ITCLSDINSKCPSELK

## Slide 44
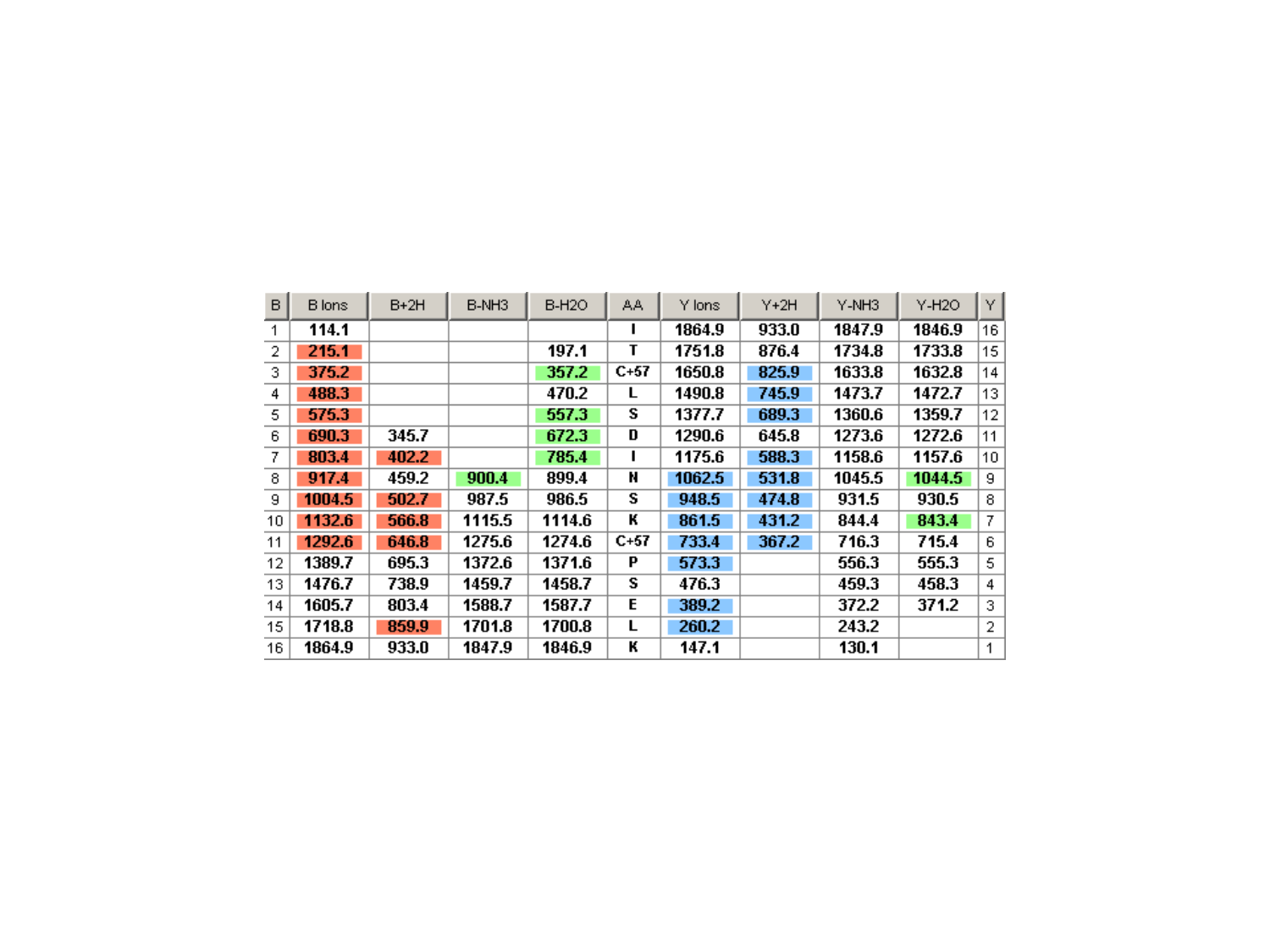

Supplement: Additional file 1 — Validation of protein identification using Scaffold software. This file provided the confidence evidence of the identification of each protein using Scaffold software. [file 1477-5956-8-64-S1.PPT]
