# Supplementary figures and images for: Proteomic profiling of proteins associated with the rejuvenation of Sequoia sempervirens (D. Don) Endl
Source: Proteome Sci. 2010 Dec 10;8:64. doi: 10.1186/1477-5956-8-64 (PMC3022872; doi:10.1186/1477-5956-8-64)

## Slide 1
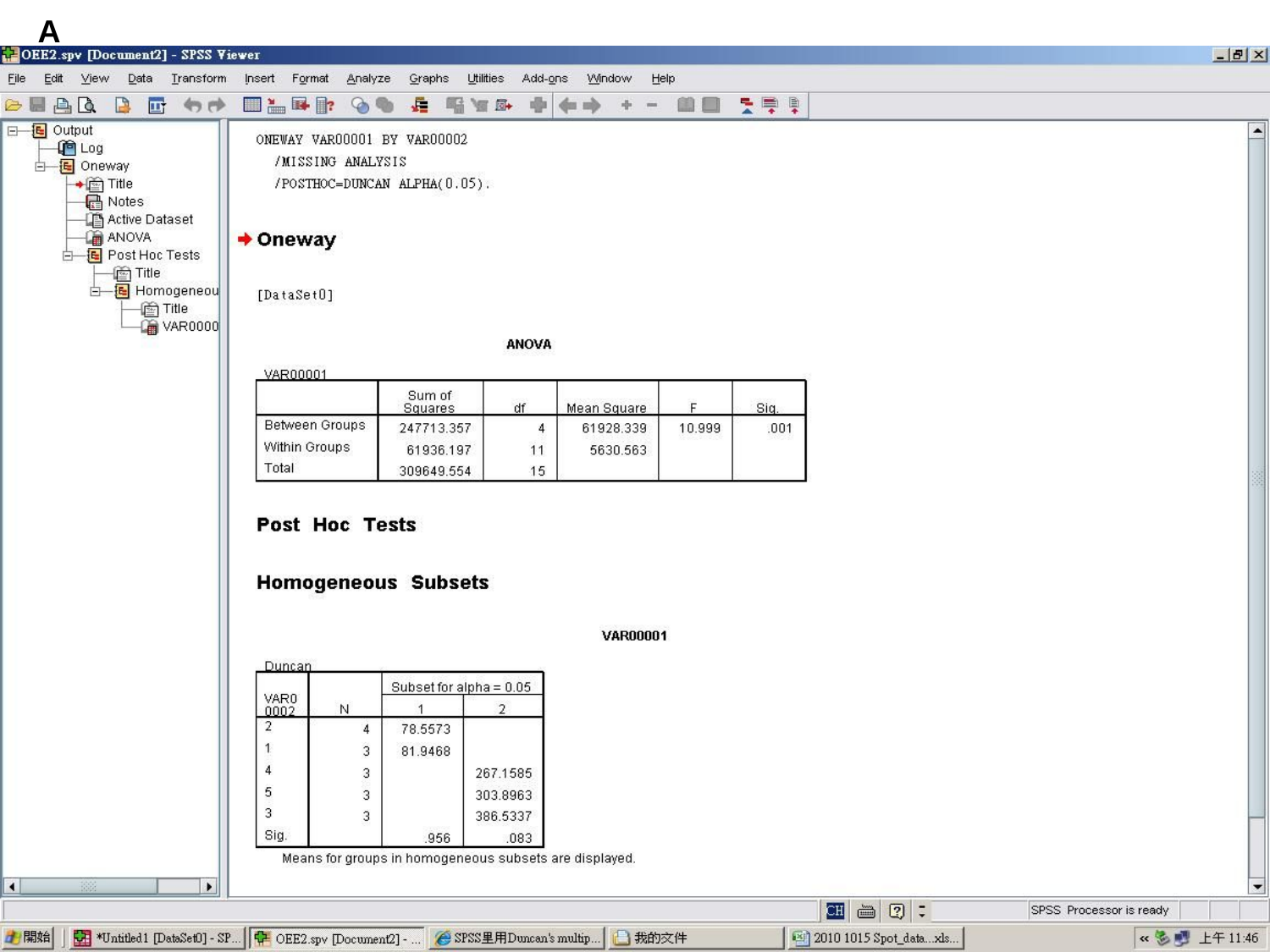

A

## Slide 2
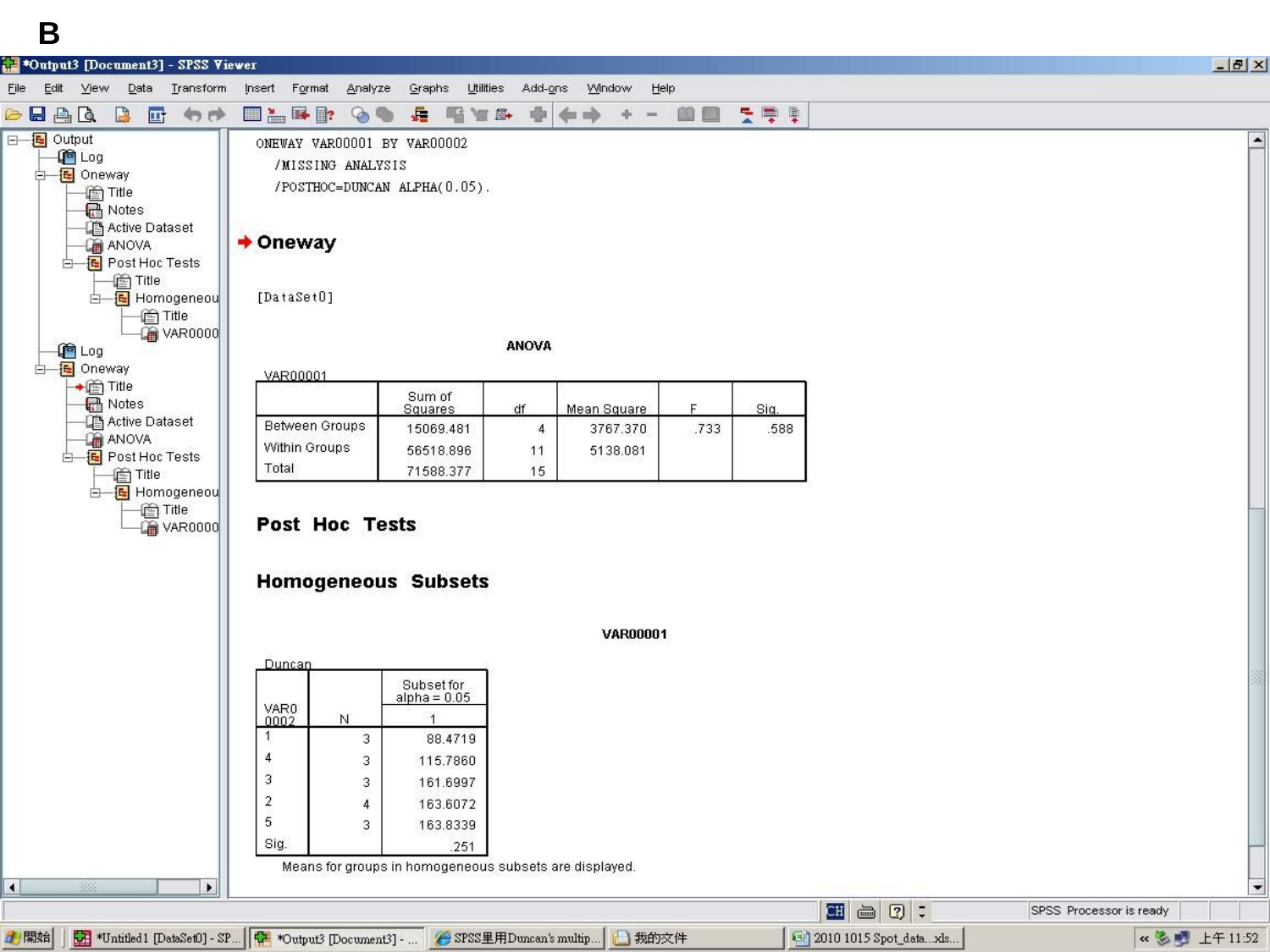

B

## Slide 3
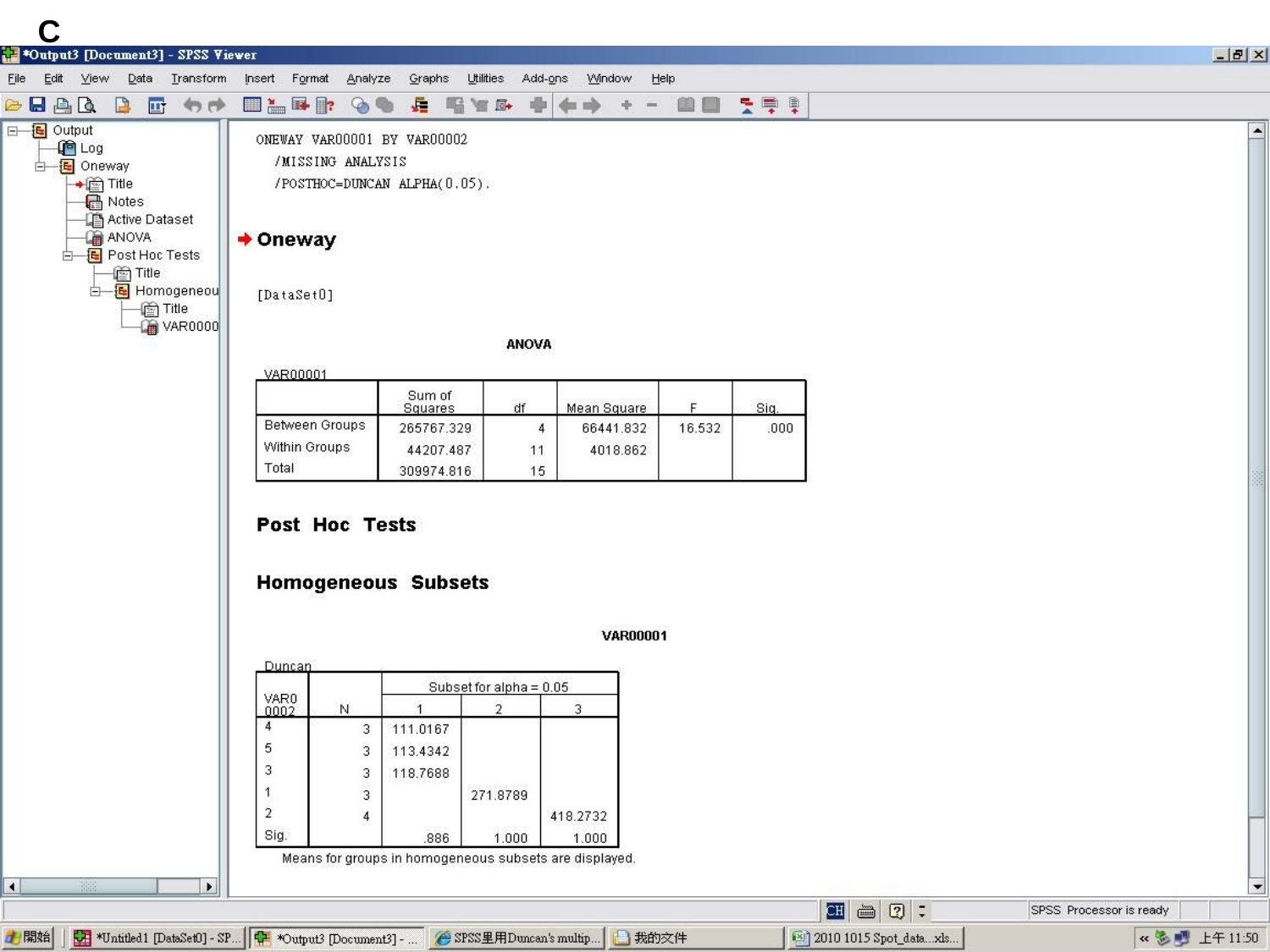

C

Supplement: Additional file 4 — ANOVA and Duncan's multiple range test for the protein abundance of spots on the 2 D gel. RAW data of ANOVA and Duncan's MRT analyses carried out using SPSS software (Ver. 16.0). A: OEE2; B:RNP; C:Thaumatin-like protein. 1:Adult76; 2:Adult94; 3:Juvenile; 4:Rejuvenated76; 5:Rejuvenated94. [file 1477-5956-8-64-S4.PPT]
